# Supplementary material for: Complex HBB gene editing outcomes revealed by a fluorescent reporter cell model
Source: Mol Ther Nucleic Acids. 2026 Feb 3;37(1):102854. doi: 10.1016/j.omtn.2026.102854 (PMC12926567; doi:10.1016/j.omtn.2026.102854)
Supplement: Document S1. Figures S1–S31 and Table S1 [file mmc1.pdf]

**OMTN, Volume *37***

## Supplemental information

# Complex HBB gene editing outcomes revealed by a fluorescent reporter cell model

**Cecile L. Karsenty, Daniel Betancourth, Mingming Cao, Quoc-Khanh Pham, So Hyun Park, and Gang Bao**

**SUPPLEMENTAL INFORMATION**

## Supplemental Tables

### Table S1. Oligonucleotide Sequences:

| **sgRNA protospacer sequences** | | **Relevant figures** |
|---|---|---|
| R-66S sgRNA | GUAACGGCAGACUUCUCCAC | Figure 1-6, S1, S5, S9, S14, S24, S26, S27, S28, S30, S31 |
| R-02 sgRNA | CUUGCCCCACAGGGCAGUAA | Figure 1-6, S1, S5, S9, S14, S26, S30 |
| R-62 sgRNA | CGUGGAUGAAGUUGGUGGUG | Figure 2, S9, S10 |
| MET-55 sgRNA | UCCACUCCUGAUGCUGUUAU | Figure 2, S9, S10 |
| HBB_c terminus sgRNA | AGCGAGCUUAGUGAUACUUG | Figure 2 |
| GFP sgRNA | GCUGAAGCACUGCACGCCGU | Figure 2 |
| **Donors** | | |
| GFP | CAGGAAACAGCTATGACAAGCTTTTAATATACTTTTTTGTTTATCTTATTTCTAATACTTTCCCTAATCTCTTTCTTTCAGGGCAATAATGATACAATGTATCATGCCTCTTTGCACCATTCTAAAGAATAACAGTGATAATTTCTGGGTTAAGGCAATAGCAATATCTCTGCATATAAATATTTCTGCATATAAATTGTAACTGATGTAAGAGGTTTCATATTGCTAATAGCAGCTACAATCCAGCTACCATTCTGCTTTTATTTTATGGTTGGGATAAGGCTGGATTATTCTGAGTCCAAGCTAGGCCCTTTTGCTAATCATGTTCATACCTCTTATCTTCCTCCCACAGCTCCTGGGCAACGTGCTGGTCTGTGTGCTGGCCCATCACTTTGGCAAAGAATTCACCCCACCAGTGCAGGCTGCCTATCAGAAAGTGGTGGCTGGTGTGGCTAATGCCCTGGCCCACAAGTATCACGGATCCGGCGCAACAAACTTCTCTCTGCTGAAACAAGCCGGAGATGTCGAAGAGAATCCTGGACCGATGGTGTCTAAGGGCGAGGAGCTGTTCACCGGGGTGGTGCCCATCCTGGTCGAGCTGGACGGCGACGTAAACGGCCACAAGTTCAGCGTGTCCGGCGAGGGCGAGGGCGATGCCACCTACGGCAAGCTGACCCTGAAGTTCATCTGCACCACCGGCAAGCTGCCCGTGCCCTGGCCTACCCTGGTGACCACCCTGACCTACGGCGTGCAGTGCTTCAGCCGCTACCCCGACCACATGAAGCAGCACGACTTCTTCAAGTCCGCCATGCCCGAAGGCTACGTCCAGGAGCGCACCATCTTCTTCAAGGACGACGGCAACTACAAGACCCGCGCCGAGGTGAAGTTCGAGGGCGACACCCTGGTGAACCGCATCGAGCTGAAGGGCATCGACTTCAAGGAGGACGGCAACATCCTGGGGCACAAGCTGGAGTACAACTACAACAGCCACAACGTCTATATCATGGCCGACAAGCAGAAGAACGGCATCAAGGTGAACTTCAAGATCCGCCACAACATCGAGGACGGCAGCGTGCAGCTCGCCGACCACTACCAGCAGAACACCCCCATCGGCGACGGCCCCGTGCTGCTGCCCGACAACCACTACCTGAGCACCCAGTCCGCCCTGAGCAAAGACCCCAACGAGAAGCGCGATCACATGGTCCTGCTGGAGTTCGTGACCGCCGCCGGGATCACTCTCGGCATGGACGAGCTGTACAAGTAACCTGAGGATCCGATCTTTTTCCCTCTGCCAAAAATTATGGGGACATCATGAAGCCCCTTGAGCATCTGACTTCTGGCTAATAAAGGAAATTTATTTTCATTGCAATAGTGTGTTGGAATTTTTTGTGTCTCTCAGTATCACTAAGCTCGCTTTCTTGCTGTCCAATTTCTATTAAAGGTTCCTTTGTTCCCTAAGTCCAACTACTAAACTGGGGGATATTATGAAGGGCCTTGAGCATCTGGATTCTGCCTAATAAAAAACATTTATTTTCATTGCAATGATGTATTTAAATGGTACCACTGGCCGTCGTTTTACA | Figure 2 |
| GFP>BFP ssODN | AAGCTGCCCGTGCCCTGGCCTACCCTGGTGACCACCCTGAGCCATGGGGTGCAGTGCTTCAGCCGCTACCCCGACCACATGAAGCAGC | Figure 2 |
| R-66S ssODN (for SCD HSPCs) | TCTGACACAACTGTGTTCACTAGCAACCTCAAACAGACACCATGGTGCACCTGACTCCTGAAGAGAAGTCTGCCGTTACTGCCCTGTGGGGCAAGGTGAACGTGGATGAAGTTGGTGGTGA | Figure 1,6, S5, S26 |
| R-66S ssODN (for S-HUDEP2) | TCTGACACAACTGTGTTCACTAGCAACCTCAAACAGACACCATGGTGCATCTGACTCCTGAAGAGAAGTCTGCCGTTACTGCCCTGTGGGGCAAGGTGAACGTGGATGAAGTTGGTGGTGA | Figure 2,3,5,6, S14, S24, S30 |
| R-02 ssODN (for SCD HSPCs) | TCAGGGCAGAGCCATCTATTGCTTACATTTGCTTCTGACACAACTGTGTTCACTAGCAACCTCAAACAGACACCATGGTGCACCTGACTCCTGAAGAGAAGTCTGCGGTTACTGCCCTGTGGGGCAAGGTGAACGTGGATGAAGTTGGTGGTGAGGCCCTGGGCAGGT | Figure 1,6, S5, S26 |
| R-02 ssODN (for S-HUDEP2) | TCAGGGCAGAGCCATCTATTGCTTACATTTGCTTCTGACACAACTGTGTTCACTAGCAACCTCAAACAGACACCATGGTGCATCTGACTCCTGAAGAGAAGTCTGCGGTTACTGCCCTGTGGGGCAAGGTGAACGTGGATGAAGTTGGTGGTGAGGCCCTGGGCAGGT | Figure 2,3,5,6, S14, S30 |

| **Primers and probes** | | |
|---|---|---|
| NGS HBB | F- TGGCCAATCTACTCCCAGGA<br>R- CTGTCTCCACATGCCCAGTT | Figure 1, 3, 6 S7, S26, S27, S31 |
| NGS Met 55 | F- TGGGCATGTGGAGACAGAGA<br>R- AGCTCACTCAGTGTGGCAAA | Figure S7 |
| ddPCR | F- CTCCACATGCCCAGTTTCTA<br>R- GCAACCTCAAACAGACACCA<br>Probe sequences<br>HBB ref (ROX)- ACGTGGATGAAGTTGGTGGTGAGG<br>RPP30 reference (VIC)- CTGACCTGAAGGCTCT | Figure 1, 3, 6, S22 |
| qPCR | F (GFP/BFP)- ATGGTGTCTAAGGGCGAGGA<br>R (GFP)- GAAGCACTGCACCCCGTAGG<br>R (BFP)- GAAGCACTGCACCCCATGGC<br>F (HBB)- GTCCACTCCTGATGCTGTTATG<br>R (HBB)- GGCACCGAGCACTTTCTTG | Figure S19 |
| cDNA NGS | F (HBB)- GGACCCAGAGGTTCTTTG<br>R (HBB)- GGGGTGAATTCTTTGCC<br>F (GFP/BFP)- CGTAAACGGCCACAAGTTCA<br>R (GFP/BFP)- CTTGTAGTTGCCGTCGTCCT | Figure S19 |
| SMRT-seq PCR1 | F- CAAGCAGAAGACGGCATACGAGATCACGTGTGCCTAGATCCTCA<br>R- AATGATACGGCGACCACCGAGATCCTGATTCTCCCACCCCCAAC | Figure 4, S16, S17 |

## Supplemental Figures

**A.** R-66S RNP

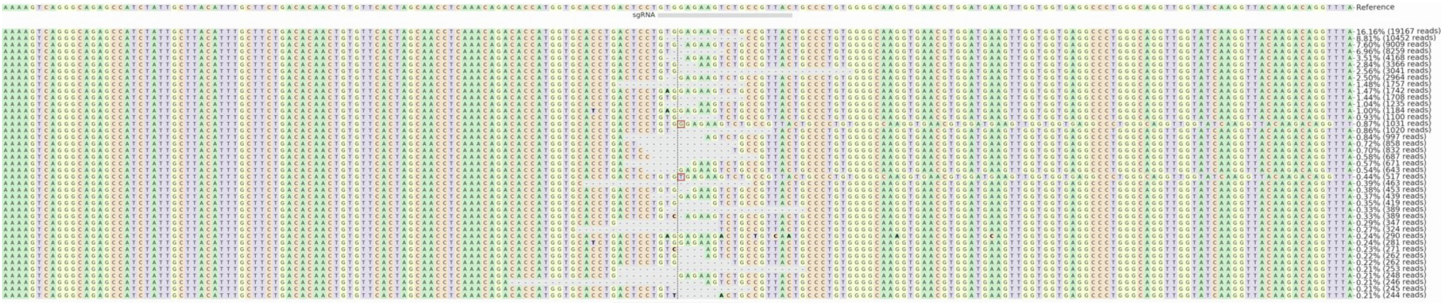

**B.** R-02 RNP

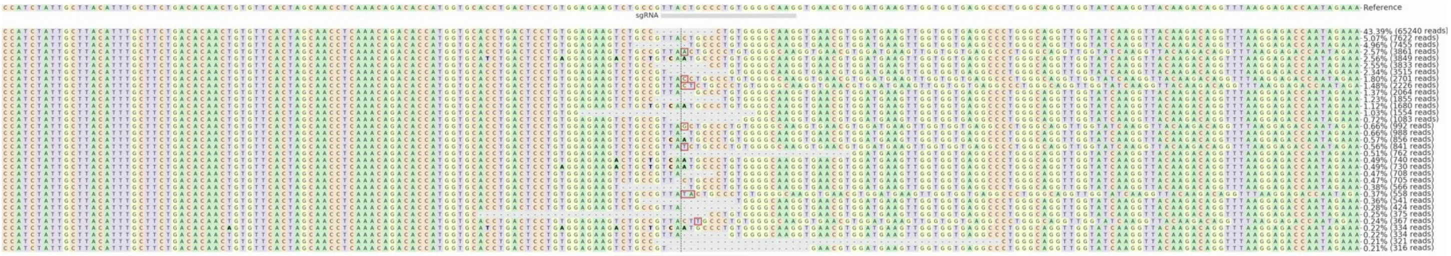

**Figure S1. gRNA specific indel profiles seen after R-66S RNP and R-02 RNP electroporation of patient derived HSPCs.** Summary of major indels generated by RNP electroporation of donor 1 HSPCs generated by NGS and CRISPResso2 analysis. gRNA specific indel profile seen when comparing (**A**) R-66S gRNA and (**B**) R-02 gRNA.

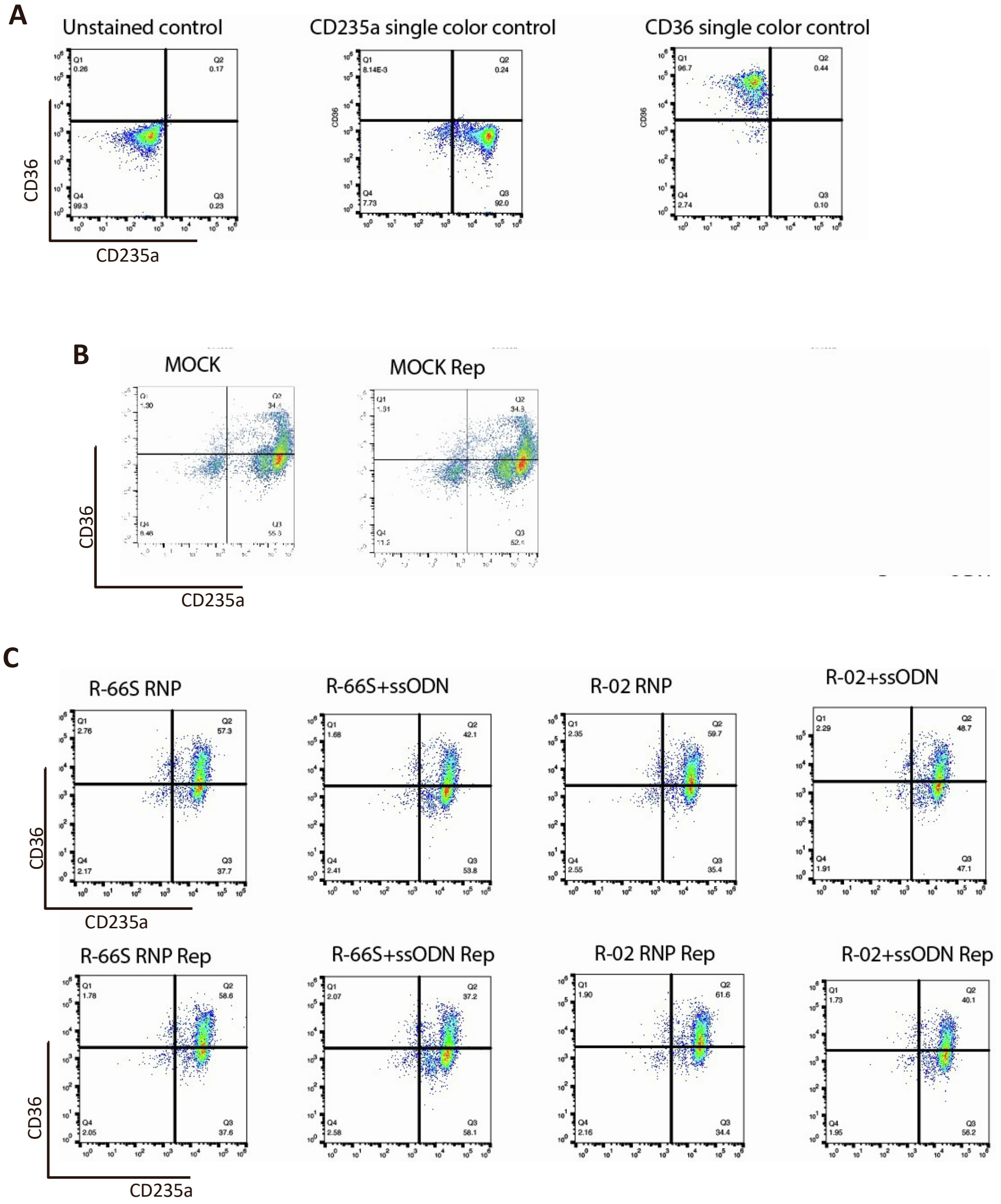


**Figure S2. Therapeutic gene editing targeting the sickle mutation in vitro affects erythroid differentiation.** Using SCD HSPCs from donor 1, R-66S RNP, R-02 RNP, R-66S+ssODN, R-02+ssODN, R-62 RNP, and Met-55 RNP were delivered via electroporation and followed by in vitro erythroid differentiation with n=2 biological replicates. On day 15 of differentiation, the impact of gene editing on maturation was assessed using erythroid-specific markers (CD36 and CD235a) and flow cytometry. Results demonstrate delayed erythropoiesis after RNP treatment based on increased percentage of double positive (CD36+/CD345a+) cells. (**A**) A gating strategy using unstained control and single-color controls for CD36 and CD235a staining on the Sony MA900 flow cytometer. (**B**) Day15, CD36 and CD235a staining for mock edited control (**C**) CD36 and CD235a positivity on day 15 of differentiation for R-66S RNP, R-66S RNP + ssODN, R-02 RNP and R-02 RNP + ssODN. The RNP-only-treated group showed delayed maturation compared with the mock- and RNP + ssODN-treated groups, with a higher proportion of immature ($CD36^+$/$CD235a^+$) cells. The addition of a corrective ssODN donor restores maturation.

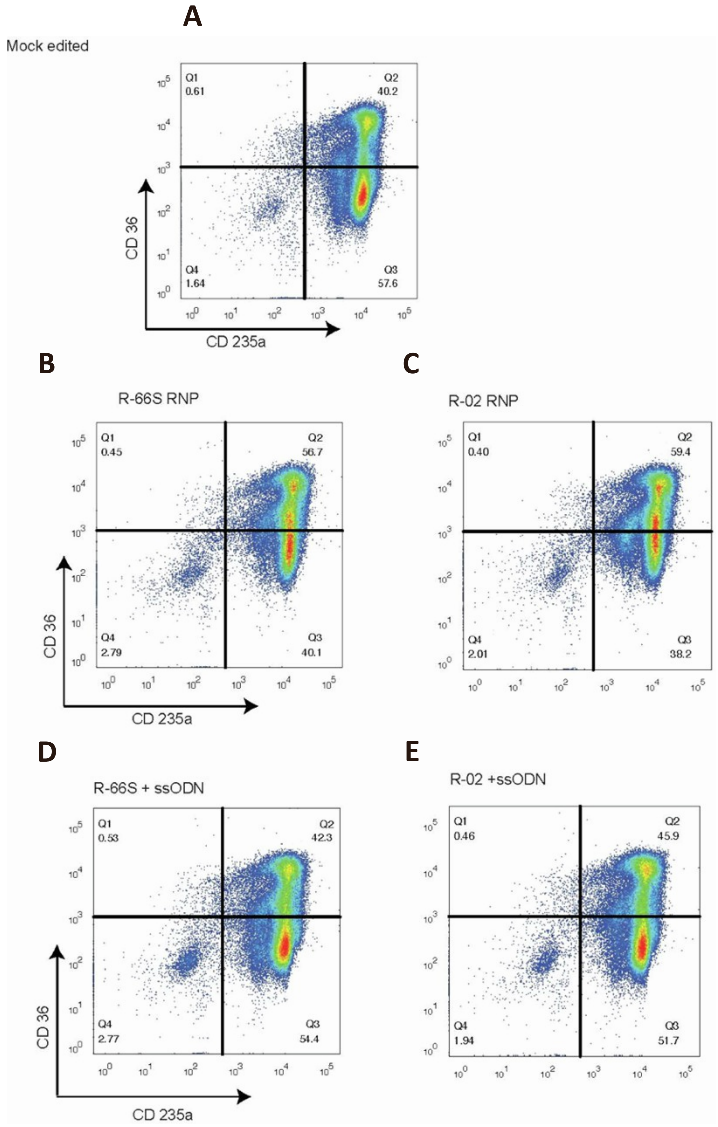


**Figure S3. Therapeutic gene editing targeting the sickle mutation in vitro affects erythroid differentiation.** *In vitro* erythroid maturation (13-day) of donor 2 SCD HSPCs showed delayed erythropoiesis after RNP treatment, with a higher proportion of immature (CD36+/CD235a+) cells compared to mature (CD36-/CD235a+). **(A)** Day 13, CD36 and CD235a staining for mock-edited control. CD36 and CD235a positivity on day 13 of differentiation for **(B)** R-66S RNP treated and **(C)** R-02 RNP treated groups, demonstrating a delay in maturation when compared to mock. and CD36 and CD235a positivity on day 13 of erythroid differentiation for **(D)** R-66S RNP + ssODN and **(E)** R-02 RNP + ssODN treated samples. Addition of corrective ssODN donor rescues maturation.

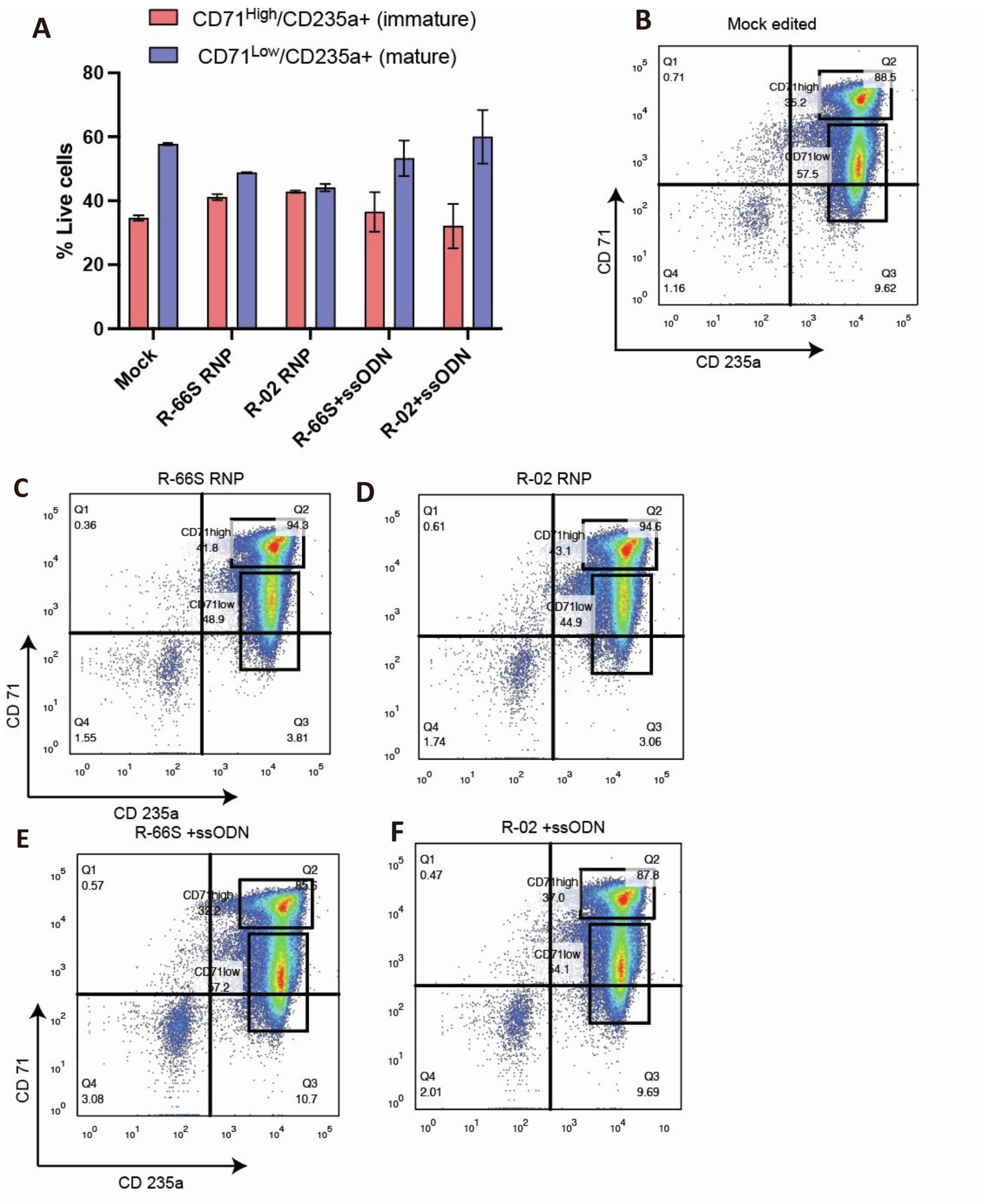


**Figure S4. Therapeutic gene editing targeting SCD mutation *in vitro* impacts erythroid differentiation, as assessed by CD71 and CD235a expression.** (**A**) *In vitro* erythroid maturation (13-day) of donor 2 SCD HSPCs showed delayed erythropoiesis after RNP treatment, with a higher proportion of immature (CD71+/CD235a+) cells compared to mature (CD71-/CD235a+); n=2 biological replicates. (**B-F**) Flow cytometry plots assessing CD71 and CD235a positivity after *in vitro* differentiation (13 days) of (**B**) mock treated control. (**C**) R-66S RNP treated cells, (**D**) R-02 RNP treated cells. (**E**) R-66S RNP + ssODN treated cells. (**F**) R-02 RNP + ssODN treated cells. Delayed maturation is observed with RNP treatment and is rescued by the addition of a corrective ssODN donor, compared with mock-treated cells.

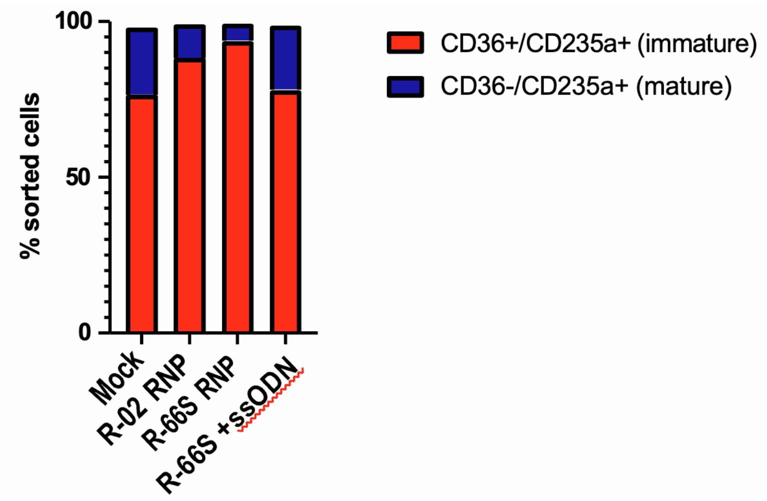


**Figure S5. RNP delivery and efficient CRISPR editing result in a higher percentage of cells with an immature phenotype, as assessed by flow cytometry, due to the underlying genotypes.** In HSPCs derived from donor 3, RNP and RNP+ssODN were delivered via electroporation using R-66s and R-02 targeting the sickle mutation. After 10 days of *in vitro* erythroid differentiation, cells were stained for CD36 and CD235a. As in donors 1 and 2, there is a clear increase in the percentage of cells with an immature phenotype in the RNP-treated groups compared to the mock-edited control. Using FACS, immature ($CD36^+$/$CD235a^{+)}$ and mature ($CD36^-$/$CD235a^+$) SCD HSPCs from donor 3 were bulk sorted for LongAmp-seq analysis.

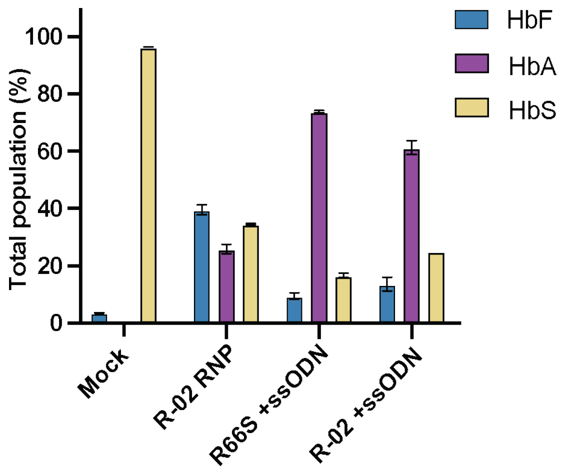


**Figure S6. HPLC analysis and quantification of HbF, HbA, and HbS after therapeutic gene editing in patient-derived HSPCs.** HPLC analysis and quantification of measurable HbF, HbA, and HbS demonstrate near-100 % HbS production in mock-treated controls. Loss of HbS is seen with R-02 RNP delivery, with apparent mild HbA induction due to HBD conversion occurring with RNP delivery alone. HbF induction is seen with RNP delivery alone. Additionally, RNP delivery resulted in several nonspecific peaks within the HPLC window, which are not shown here. Addition of corrective donor results in a significant increase in HbA with minimal residual HbS. N=2 technical replicates.

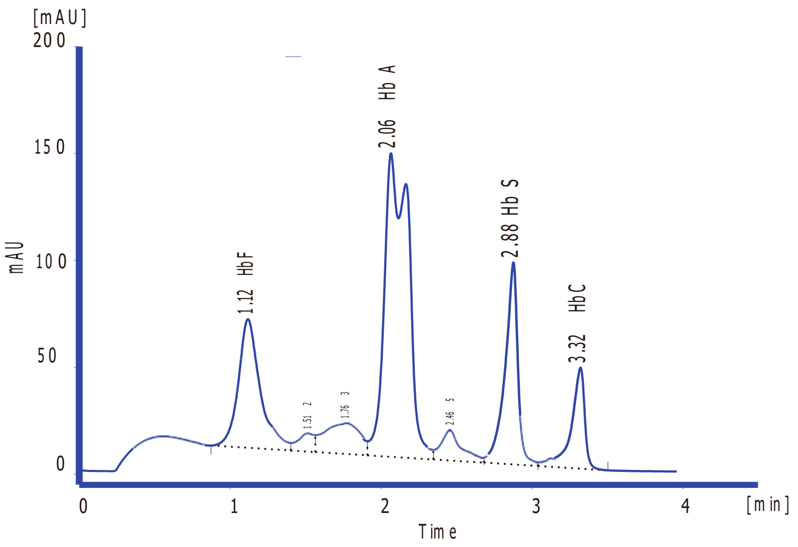


**Figure S7. HPLC reference control demonstrating reference migration patterns and retention times for hemoglobin F, A, S, and C.**

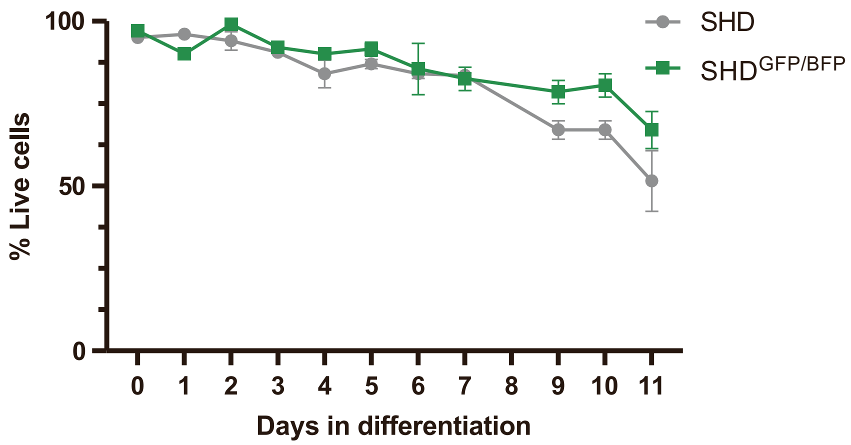


**Figure S8. SHD gene tagging does not affect viability or the ability to undergo in vitro erythroid differentiation.** The viability of parental S-HUDEP2 and SHD$^{GFP/BFP}$ models is compared throughout in vitro differentiation using trypan blue staining and shows a similar trend. No significant differences were observed; SHD$^{GFP/BFP}$ maintained viability compared to parental S-HUDEP2.

## A. R-02 RNP

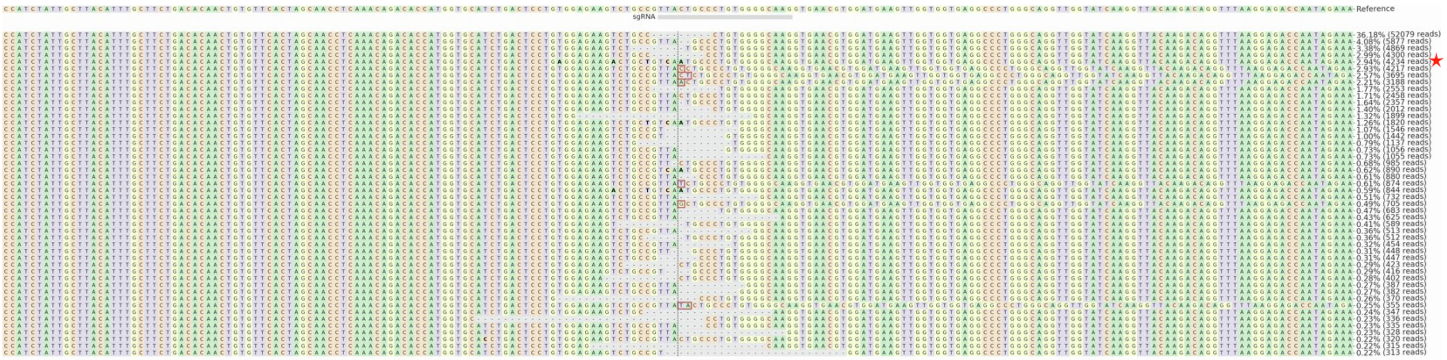


## B. R-66S RNP

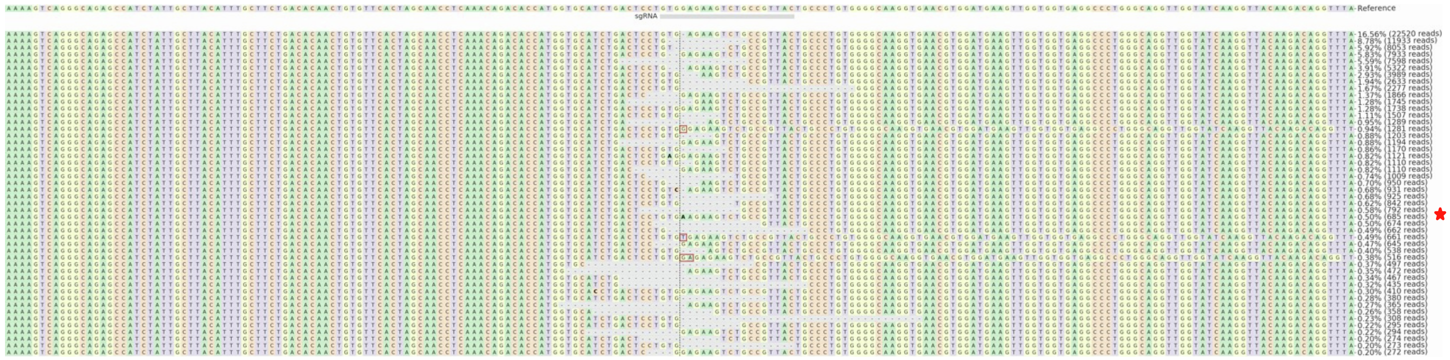


## C. R-62 RNP

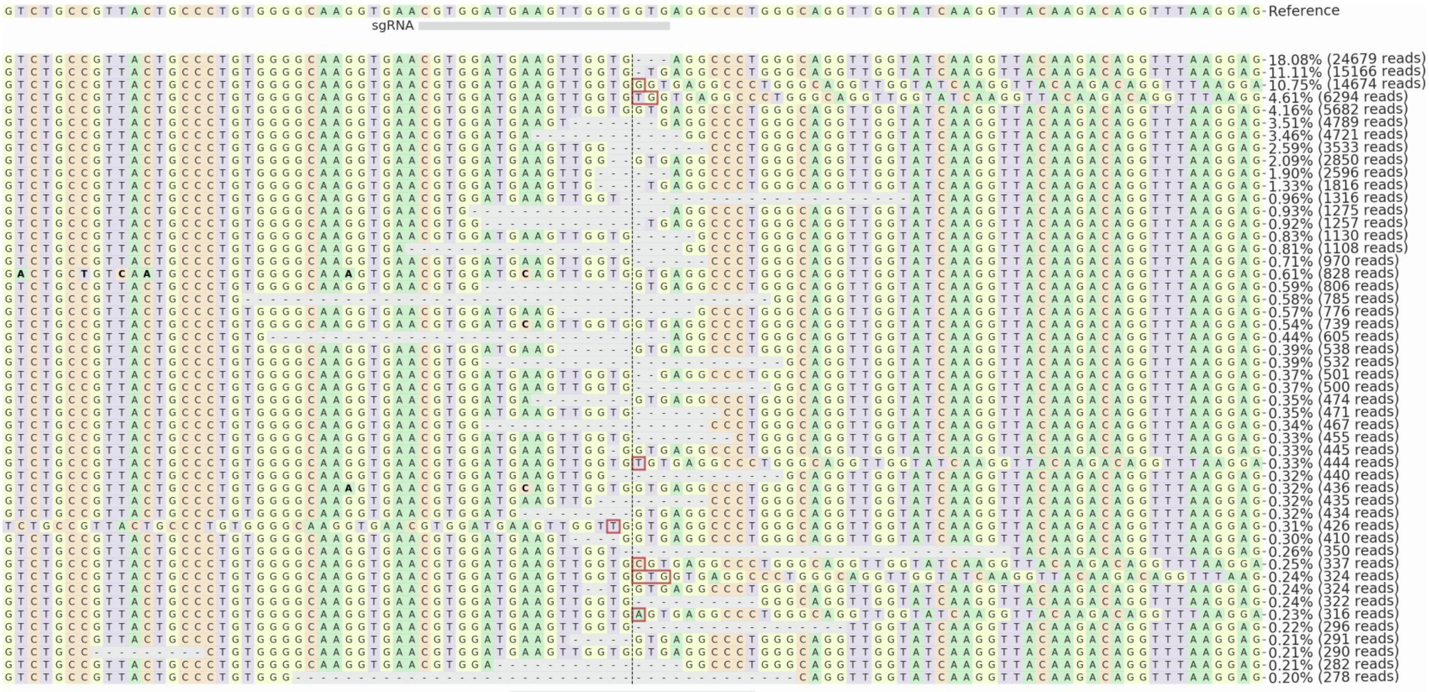

**D.** Met-55 RNP

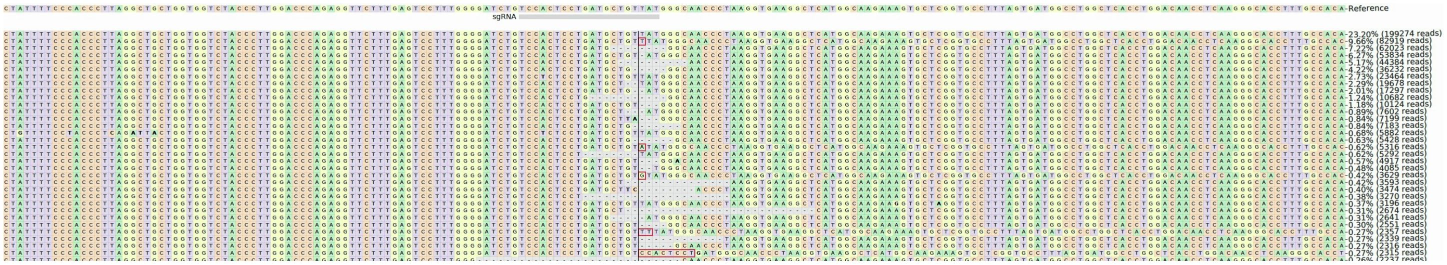

**Figure S9. Unique indel patterns after R-66S RNP, R-02 RNP, R-62 RNP, and Met-55 RNP mediated editing in SHD$^{GFP/BFP}$.** CRISPResso2 NGS analysis for each gRNA demonstrates unique indel patterns at the on-target cut site. Summary of the most frequent indels in the bulk population for each gRNA is shown. Red stars indicate alleles that have undergone HBD conversion due to R-66S and R-02 editing. This results in a T-to-A correction, which can repair the SCD mutation in the chimeric HBB-HBD gene.

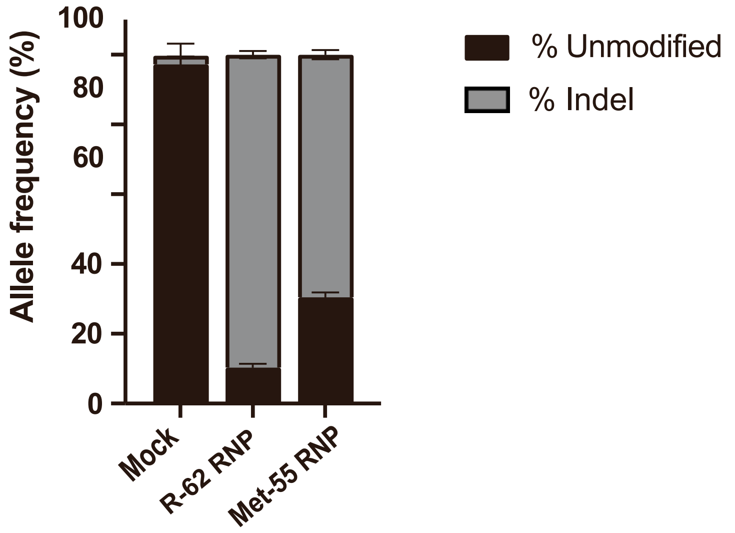


**Figure S10. High bulk editing rates for R-62 and Met-55 gRNA in SHD$^{GFP/BFP}$**. NGS editing quantification demonstrating high editing rates in SHD$^{GFP/BFP}$ for both gRNAs.

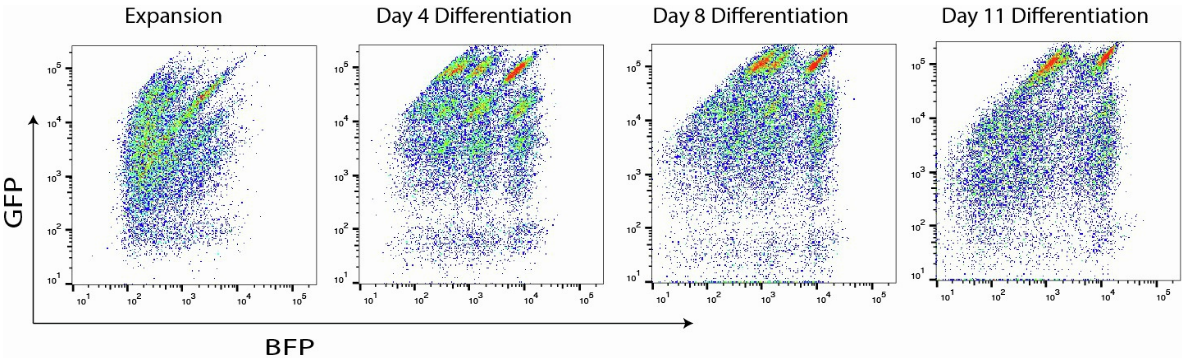


**Figure S11. Beta-globin expression and cellular viability drive changes in GFP and BFP MFI.** GFP and BFP MFI were measured throughout the in vitro culture of the R-66S RNP-treated SHDGFP/BFP model, demonstrating changes in MFI with differentiation. A clear distinction between the unique clusters is apparent only during erythroid differentiation after culture, with induction of β-globin transcription and translation. A clear pattern was observed as early as 2 days after the initiation of differentiation. However, late in differentiation (day 11), clusters based on GFP and BFP MFI are less distinct due to decreased cell viability and health.

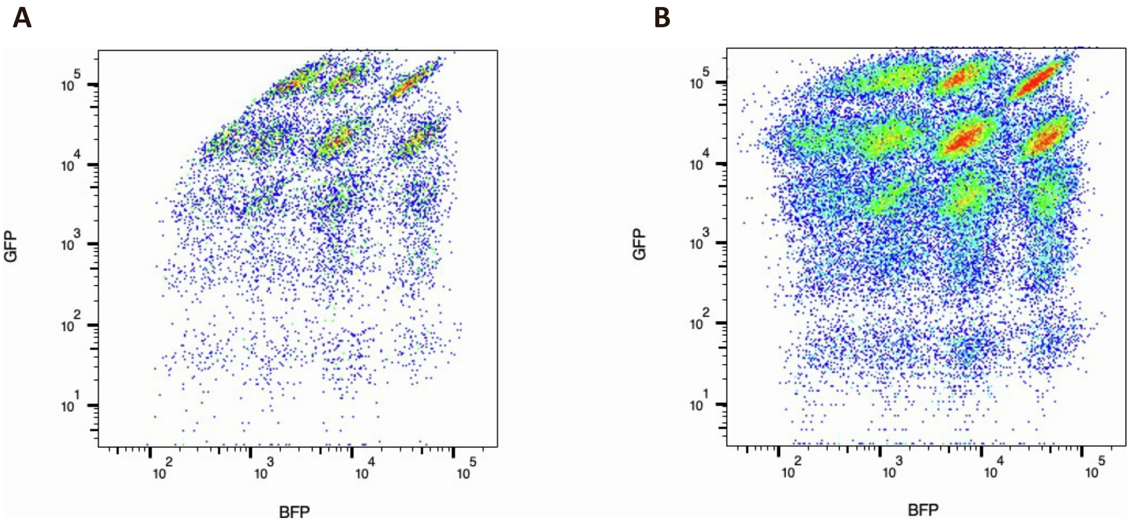


**Figure S12. Improved separation of clusters after R-66S RNP editing based on GFP and BFP MFI.** BFP MFI is lower than GFP MFI, resulting in poor separation, particularly when GFP MFI is high. We improved cluster separation after R-66S editing by generating a clone from the initial $SHD^{GFP/BFP}$ model and updating the experiment-specific compensation on the BD Melody flow cytometer. This resulted in improved separation after R-66S RNP editing. On day 4 of differentiation, we compared separation using standard BD Melody FC bead compensation with experiment-specific compensation using GFP- and BFP-single-fluorophore controls. **(A)** Separation seen after R-66S RNP gene editing on day 4 of erythroid differentiation with standard flow cytometry compensation. **(B)** Separation was observed using experiment-specific, updated compensation, resulting in improved distinction among clusters. Improved separation of GFP and BFP was observed in clusters with high GFP and varying BFP levels, allowing us to distinguish $GFP^{high}BFP^{low}$ from $GFP^{high}BFP^{neg}$, resulting in higher resolution. The resolution was further improved by using the SONY MA900 cell sorter for subsequent experiments, as described in the main text.

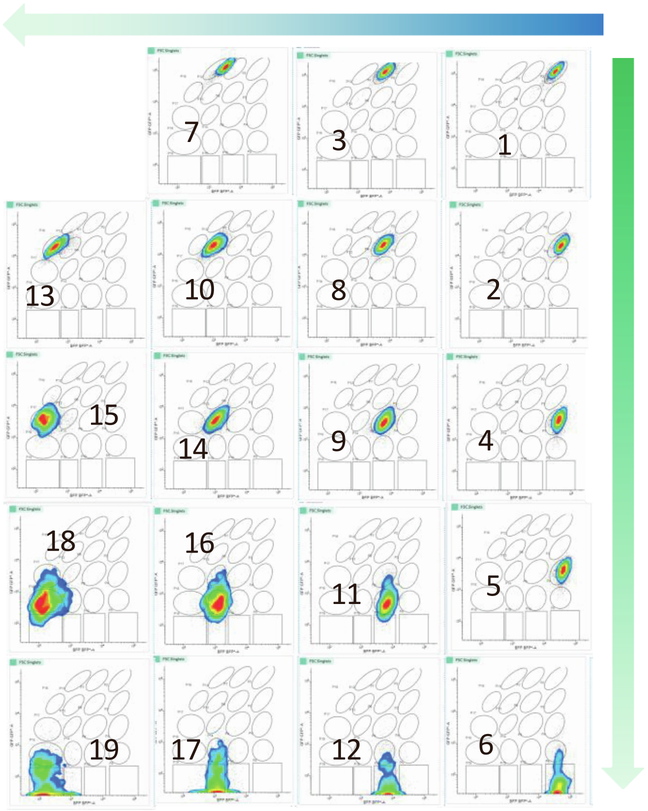


**Figure S13. Unique clusters based on GFP and BFP MFI due to underlying genotypes.** Bulk sorted 19 clusters defined based on GFP+BFP MFI remain within the initial gating used for sorting after further culture *in vitro,* confirming distinct MFI changes are due to unique genotypes in each cluster rather than *HBB* promoter strength and cell status. Clusters were numbered according to the sum of GFP and BFP MFI, ordered from highest (Cluster 1) to lowest (Cluster 19).

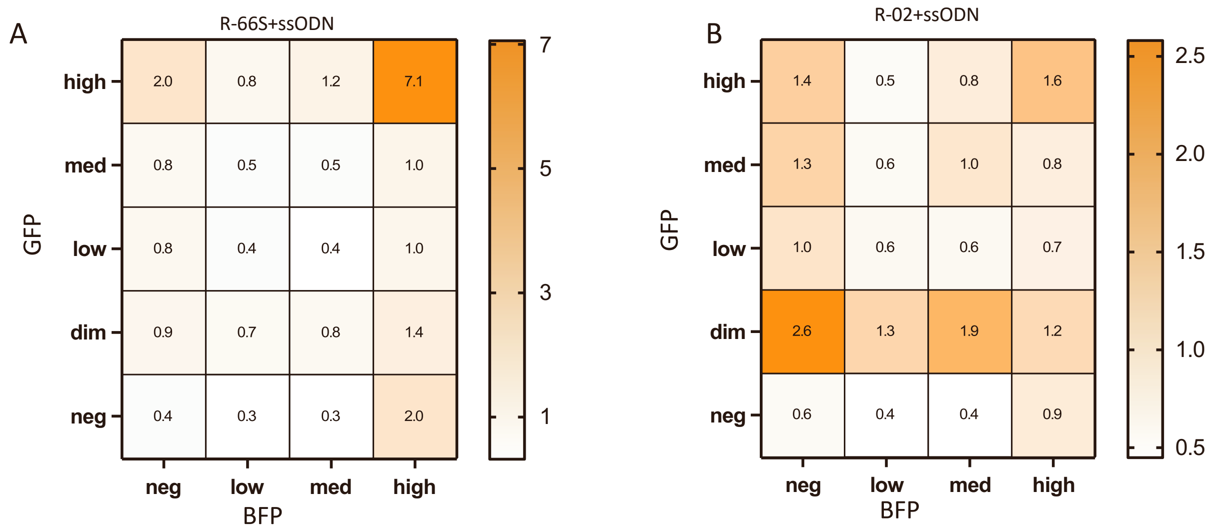


**Figure S14. Addition of an ssODN corrective donor, delivered as an RNP, with both R-02 and R-66S results in a decrease in the percentage of cells with small indels and large deletions.** Fold change in percentage of cells shown comparing **(A)** R-66S+ssODN to R-66S RNP and **(B)** R-02+ssODN and R-02 RNP alone. With both gRNAs, an overall decrease in clusters enriched in small indels and large deletions is seen. Heatmaps display means from n= 2 electroporations.

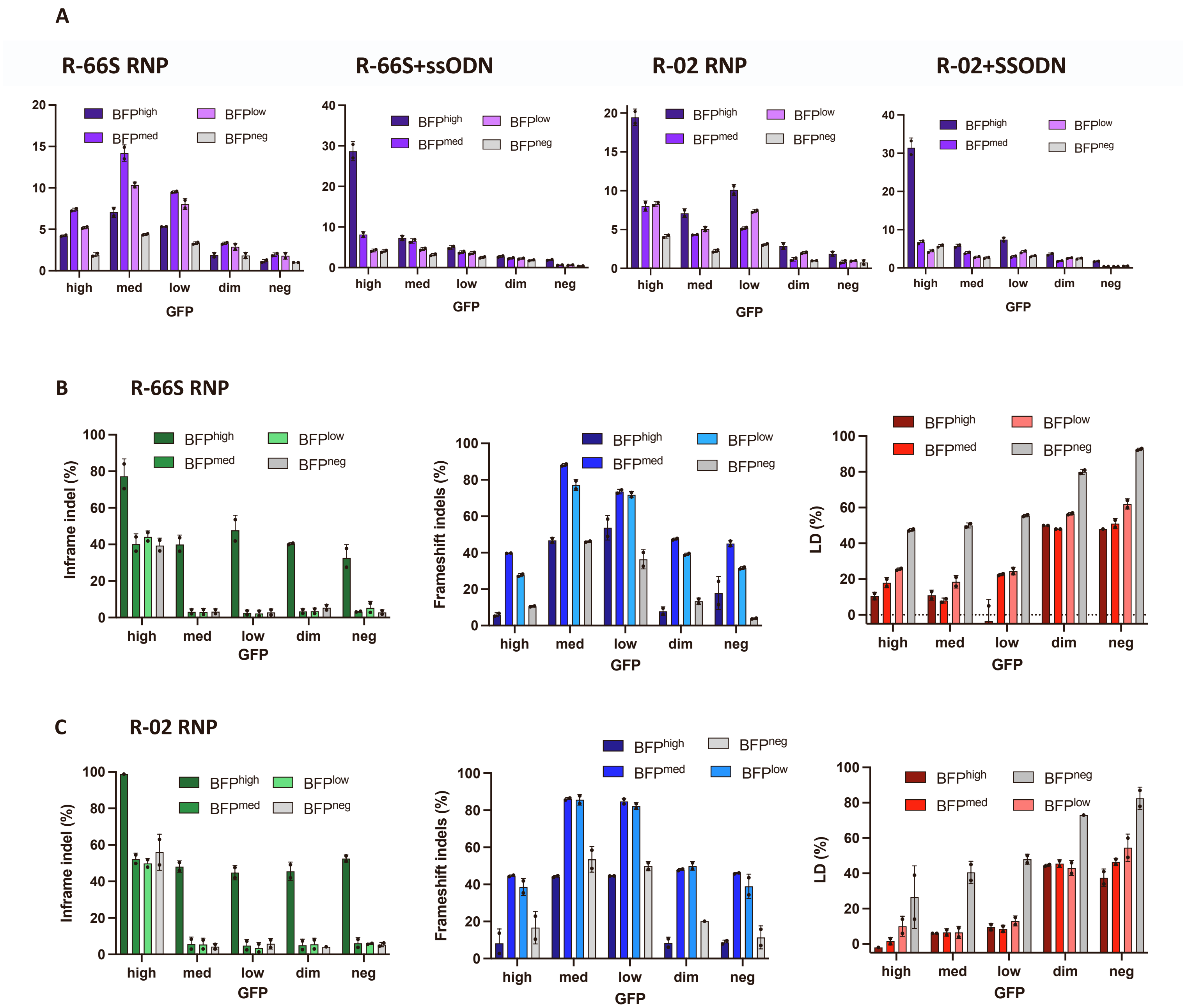


**Figure S15. Cell number quantification and indel profiles across each cluster based on GFP and BFP MFI after R-66S and R-02 gRNA editing. (A)** After efficient editing, the percentage of cells in each cluster reflects gRNA-specific editing outcomes across replicates, with per-replicate values and SD error bars. **(B)** Percentage of in-frame indels, frameshift indels, and LD across two independent replicates after R-66S RNP electroporation, replicate values and SD error bars shown. **(C)** Percentage of in-frame indels, frameshift indels, and LD across two independent replicates after R-02 RNP electroporation, individual replicates and SD error bars shown.

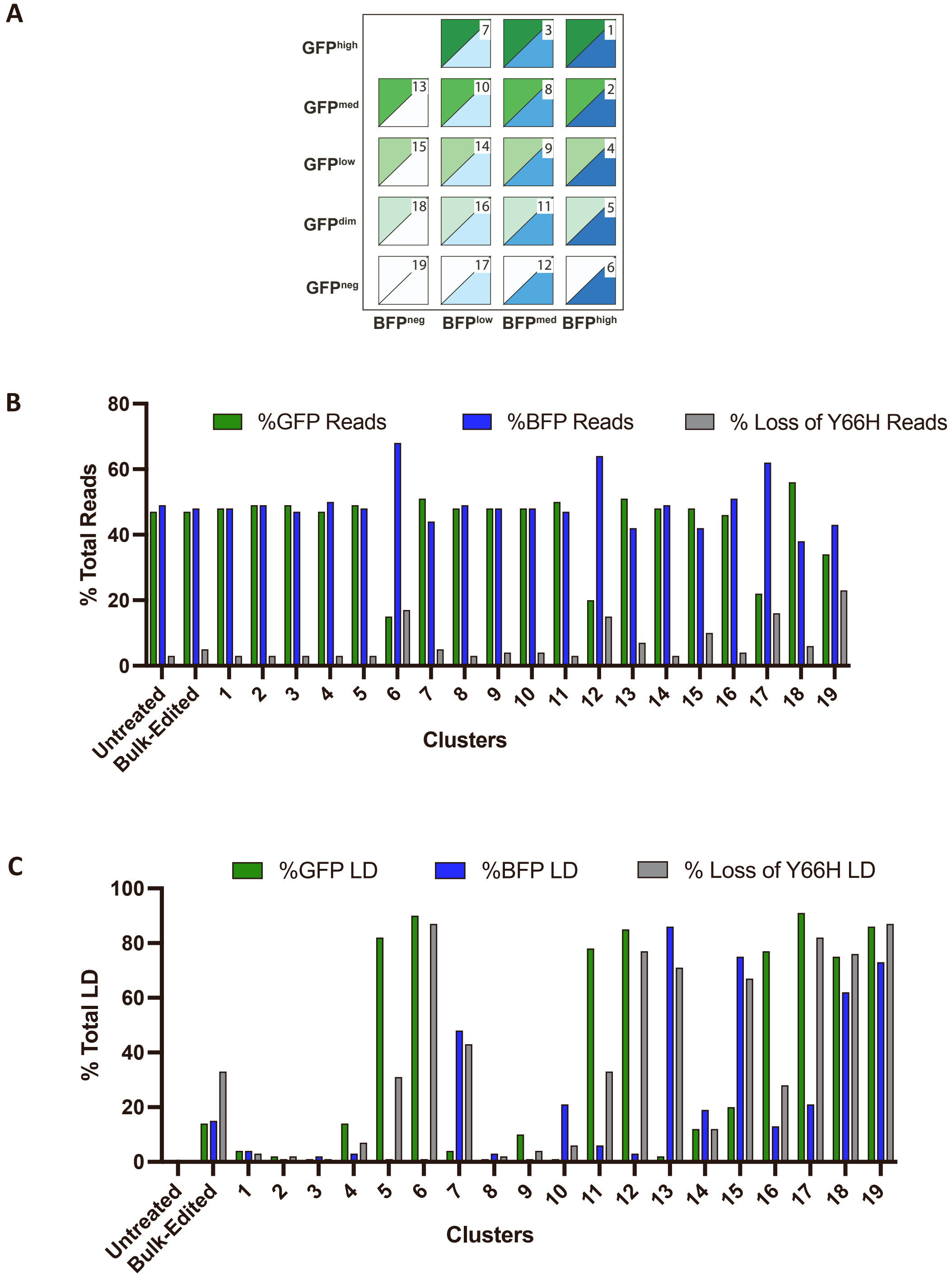


**Figure S16. SMRT-Seq analysis enables accurate quantification of allele-specific editing.** SMRT-seq analysis on R-66S RNP edited SHD$^{GFP/BFP}$ sorted into 19 clusters as shown in Figure S13 and applied to long-range PCR encompassing R-66S cut site, and Y66H mutation site, and reads were mapped to the corresponding GFP and BFP allele. Additionally, "loss of Y66H" alleles were identified, corresponding to reads with large deletions extending beyond the SNP, making it impossible to map the read to a specific GFP or BFP allele. **(A)** Previously defined cluster numbering 1-19 based on MFI **(B)** Allele-specific reads mapped based on GFP and BFP for unedited control, bulk edited and sorted clusters (1-19). **(C)** Large deletion rates across sequenced reads for each cluster demonstrate enrichment of large deletions with loss of GFP or BFP in addition to high rates of large deletions or large gene modification resulting in loss of the Y66H mutation site.

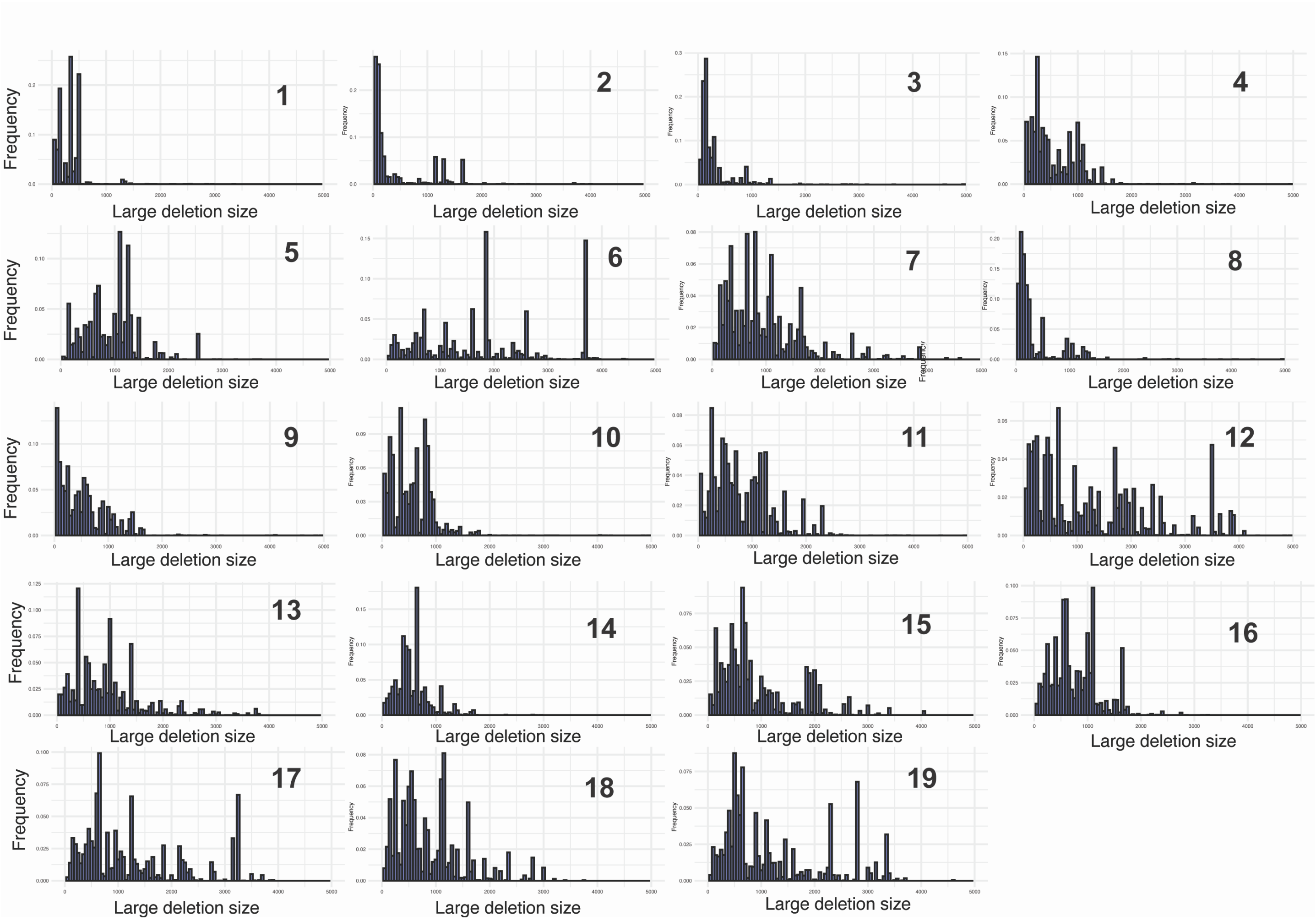


**Figure S17. SMRT-Seq analysis enables the identification of large deletions that are missed in bulk cellular analysis after R-66S RNP editing.** SMRT-seq enables size distribution analysis of large deletions in each of the predefined 19 clusters after R-66S RNP editing. High rates of very large deletions are seen in $GFP^{neg}$ and/or $BFP^{neg}$ clusters.

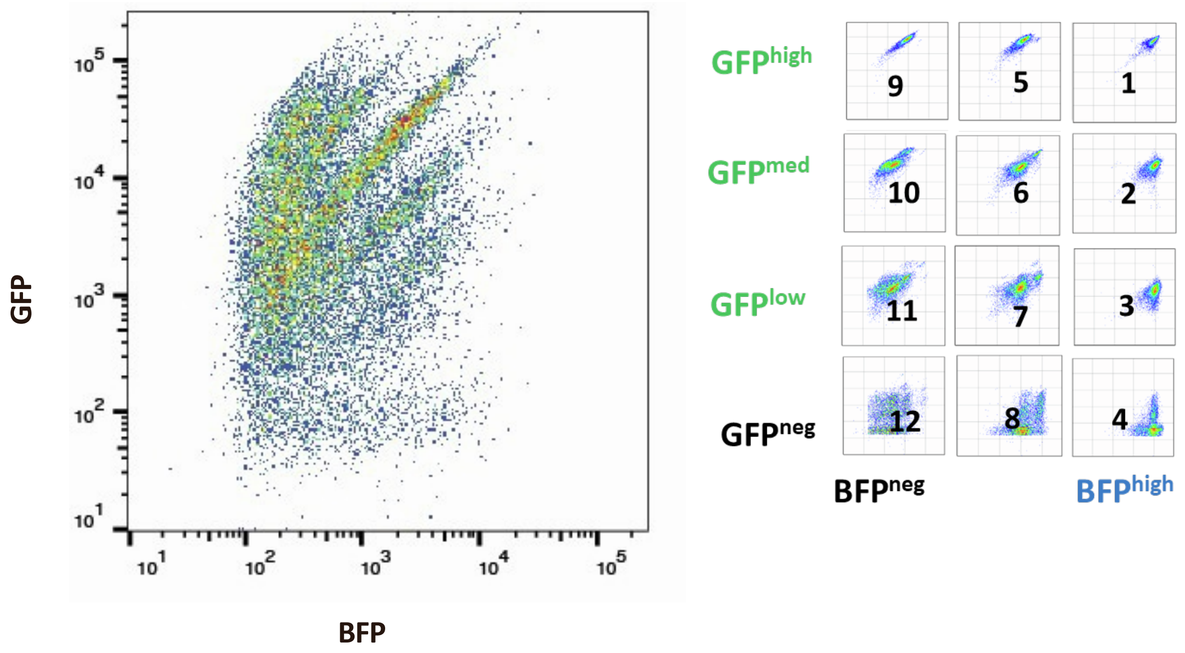


**Figure S18. Unique clusters can be identified based on GFP and BFP MFI in bulk R-66S RNP *in vitro* expansion culture.** GFP and BFP MFI changes were observed in expansion culture after R-66S RNP delivery, indicating that 12 unique clusters were used for mRNA and protein analyses due to a short lifespan after differentiation and poor expansion. Despite less distinct separation due to lower beta-globin promoter activity during expansion culture, 12 sorted clusters remained within the initial gate after *in vitro* expansion.

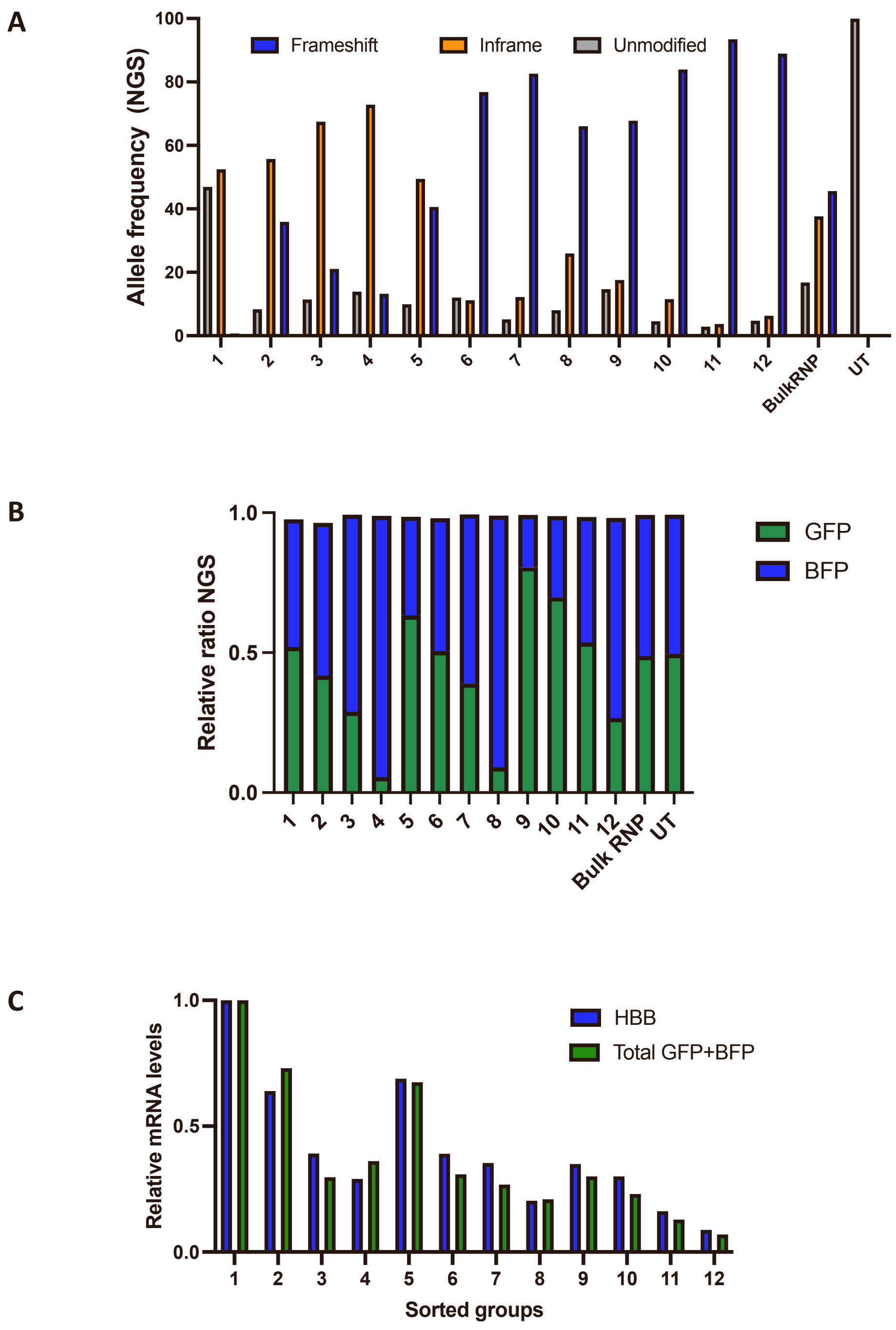


**Figure S19. GFP and BFP expressions correlate clearly with HBB expressions across 12 sorted clusters in expansion culture after R-66S RNP delivery based on cDNA and mRNA levels. (A)** cDNA NGS performed on clusters 1-12, demonstrating similar incidence in frameshift indels as seen in gDNA NGS, confirming that certain frameshift indels allow for transcription. **(B)** cDNA NGS showing relative ratio of GFP to BFP in clusters 1-12, bulk RNP treated and UT samples, a clear 1:1 ratio of GFP to BFP seen in untreated control, and changes in clusters 1-12 correlate with changes seen in MFI. **(C)** qPCR showing relative mRNA levels of HBB and GFP+BFP demonstrating a clear correlation of GFP/BFP expression with HBB expression.

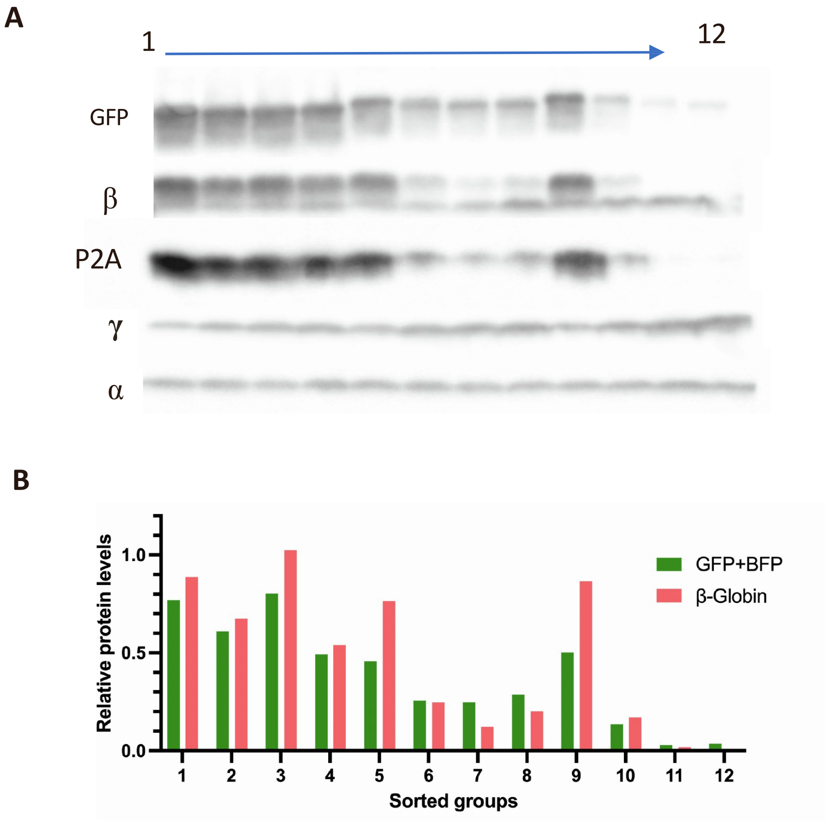


**Figure S20. Protein analysis demonstrates a clear correlation between GFP/BFP translation and HBB translation (A)** Western blot imaging of each 1-12 sorted cluster in expansion culture for GFP+BFP, β-globin, P2A, γ-globin, and $\alpha$-globin. A clear correlation between loss of GFP+BFP protein production and β-globin production, with stable γ- and α-globin production, was observed. **(B)** Western blot relative quantification analysis using ImageJ of GFP+BFP and β-globin normalized to $\alpha$-globin for each sorted cluster, demonstrating a strong correlation between GFP/BFP expression and β-globin production.

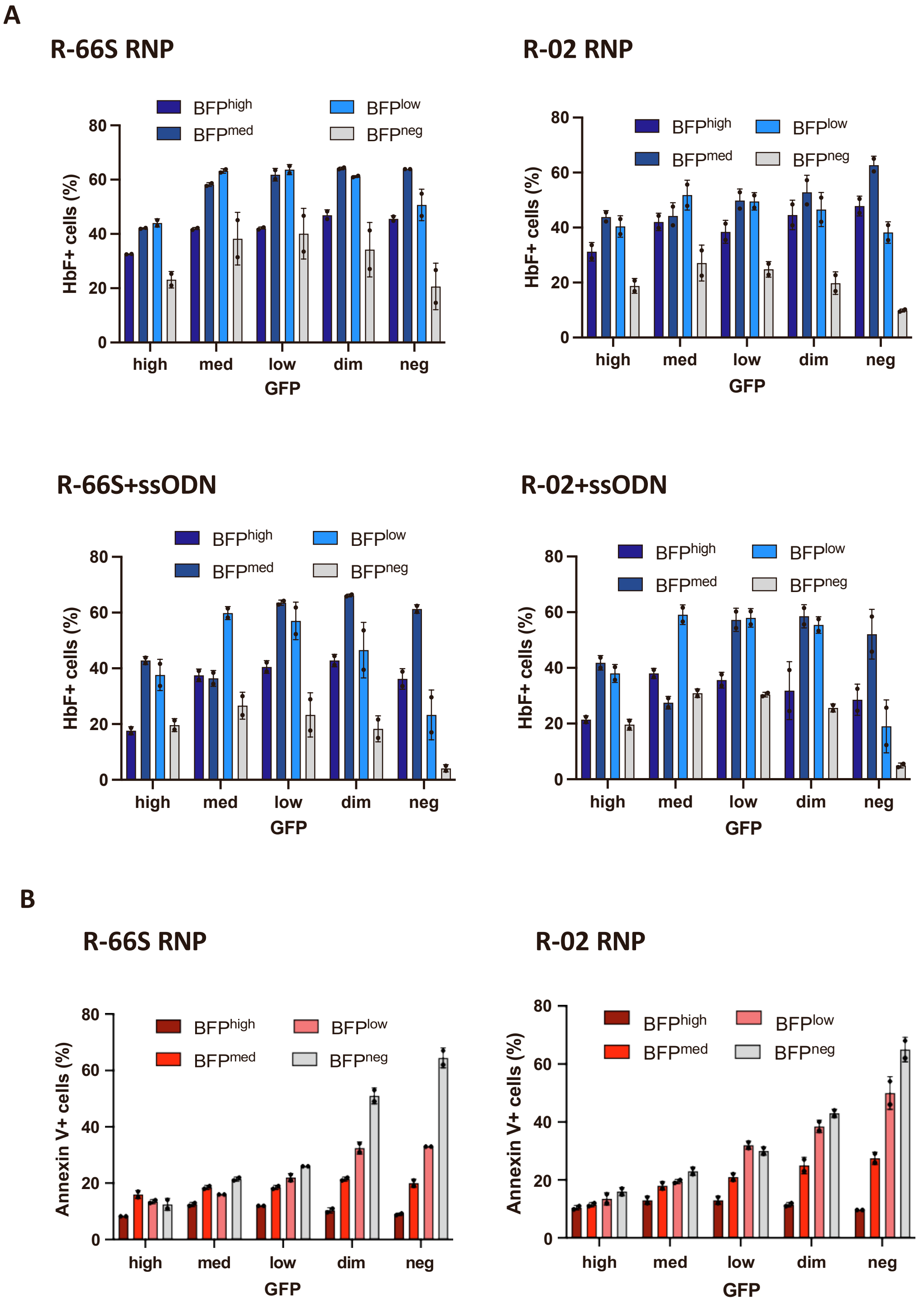


**Figure S21. HbF induction and Annexin V+ vary significantly across clusters after R-66S and R-02 therapeutic gene editing, attributable to distinct genotypes. (A)** After efficient editing, the percentage of HbF+ cells in each cluster reflects gRNA-specific editing outcomes with and without ssODN correction, shown across replicates with per-replicate values and SD error bars. **(B)** Percentage Annexin V+ cells in each cluster across two independent replicates after R-66S RNP and R-02 RNP electroporation, replicate values and SD error bars shown.

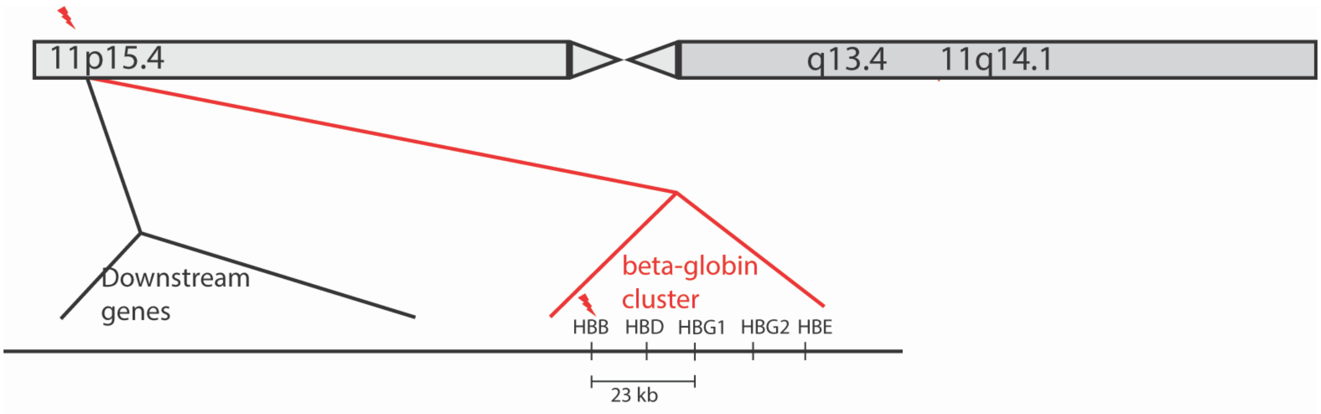


**Figure S22. Schematic of the globin locus.** HBG is located ~21 kb upstream of HBB, and large deletions extending beyond HBB could disrupt the HBG region.

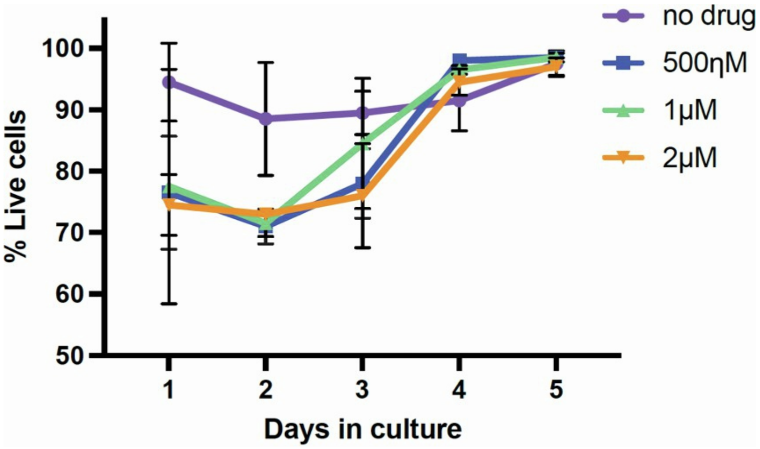


**Figure S23. M3814 was well-tolerated by SHD$^{GFP/BFP}$.** M3814 dose curve well tolerated by SHD$^{GFP/BFP}$ based on cell viability measured via trypan blue. Initial toxicity was seen with drug incubation across all doses; however, recovery was seen within 4 days of editing based on viability. N=2, technical replicates.

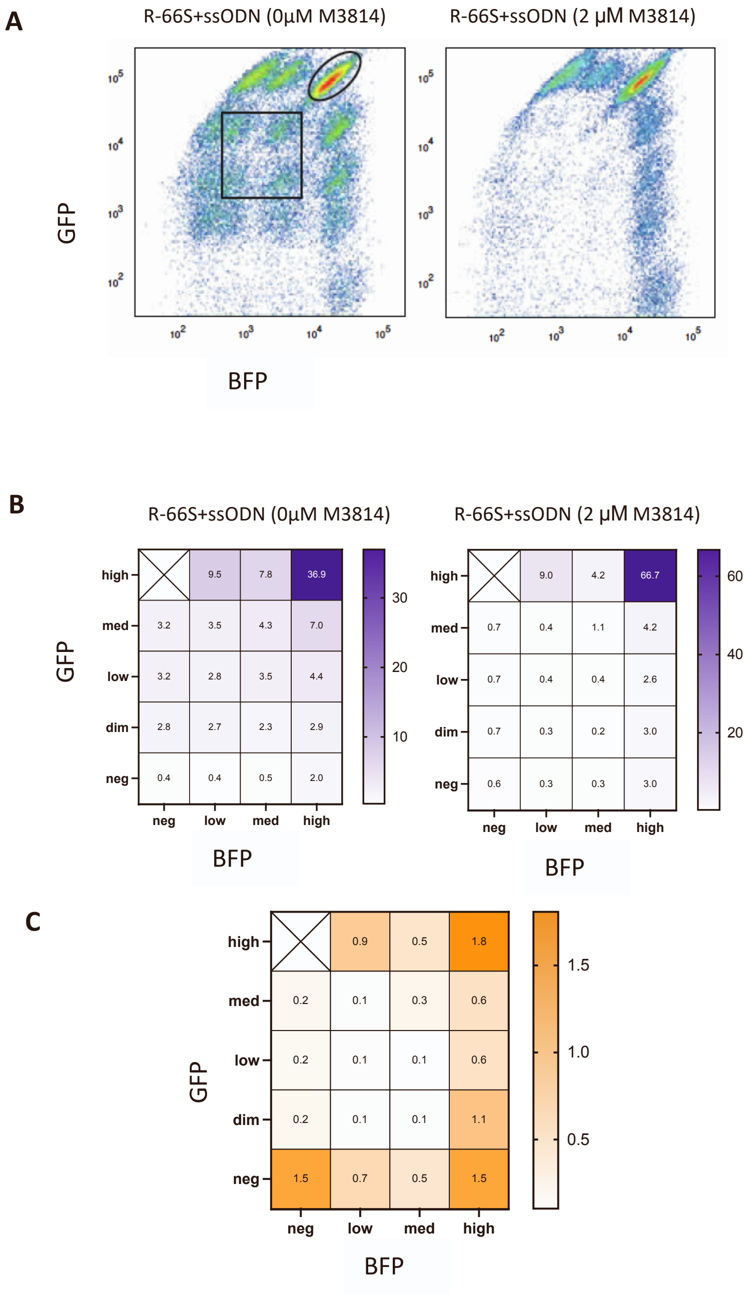


**Figure S24. Validated platform for evaluating the impact of HDR enhancer M3814 on HDR rates and DSB cellular repair mechanisms.** R-66S RNP and ssODN delivered via electroporation to $SHD^{GFP/BFP}$ followed by treatment with M3814, a potent DNA-dependent protein kinase (DNA-PK) inhibitor. Dose curve from 0.5 to 2 μM; M3814 was added immediately post-editing, and cells were incubated with M3814 for 24 hours, after which M3814 was removed. **(A)** Inhibition of NHEJ resulted in a dose-dependent increase in $GFP^{high}BFP^{high}$ cells on day 4 of erythroid differentiation, indicating enhanced HDR. **(B)** The percentage of cells across 20 previously defined clusters was quantified after R-66S+ssODN editing, with and without M3814 incubation. Again, demonstrating an increase in the percentage of $GFP^{high}BFP^{high}$ cells with the addition of M3814. Additionally, blocking NHEJ with M3814 reduces the percentage of cells enriched for bi-allelic frameshift indels, as evidenced by fewer cells in clusters with intermediate GFP and BFP. However, an increase in $GFP^{neg}BFP^{neg}$ and $GFP^{neg}BFP^{high}$ clusters raises concern about higher rates of large deletions and LOA with NHEJ inhibition, although this is not statistically significant. N=2, technical replicates**.** **(C)** Fold change in percentage of cells in each cluster after R-66S+ssODN editing with and without M3814 incubation.

**R-66S**

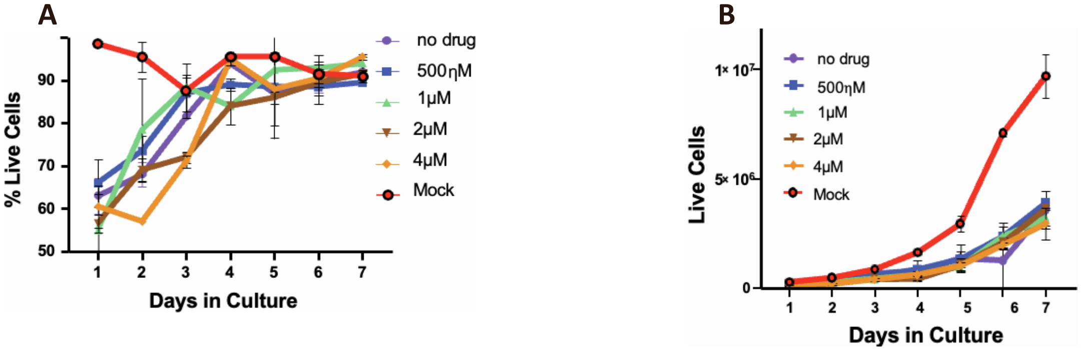


**R02**

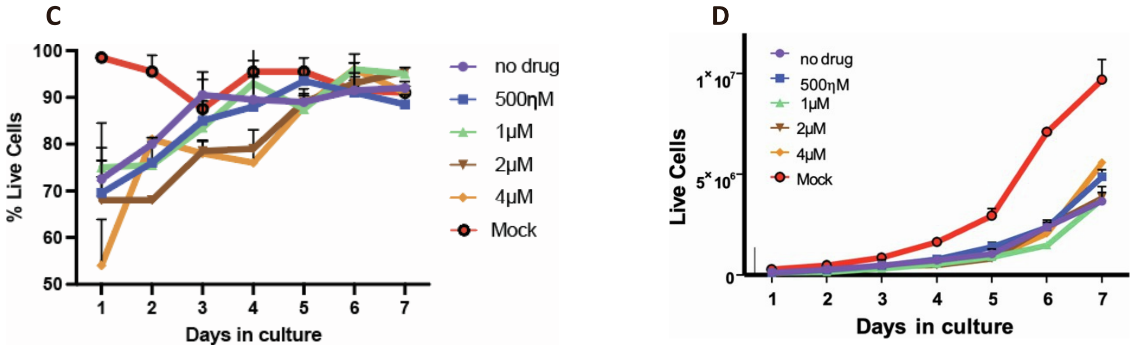


**Figure S25. M3814 was well tolerated by patient-derived HSPCs after R-66S+ ssODN and R-02 ssODN gene editing *in vitro*.** Cell viability was measured by trypan blue staining, and expansion was assessed daily for 7 days after electroporation and M3814 drug treatment. **(A)** Impact of M3814 on cell viability following R-66S+ssODN *in vitro* editing based on trypan blue staining. Initial toxicity was seen with electroporation and drug treatment, with the most significant toxicity seen in the highest drug concentration; however, recovery was seen across all conditions. **(B)** Preserved cellular expansion with M3814 treatment and R-66S +ssODN electroporation. Among edited samples, no significant difference in expansion was observed across groups, regardless of M3814 dose. Slow expansion, as expected, was seen within the initial 72-96 hours of electroporation, followed by a more rapid expansion rate. Mock-treated control showed expected robust expansion throughout. **(C)** Impact of M3814 on cell viability following R-02 + ssODN *in vitro* editing based on trypan blue staining. Dose-dependent toxicity seen with initial M3814 incubation followed by complete recovery by day 7 of *in vitro* culture. **(D)** Preserved cellular expansion with M3814 treatment after electroporation with R-02+ssODN. Slowed expansion with electroporation and drug treatment, like results seen with R-66S +ssODN. No significant difference was observed in the M3814 dose curve. N=2, technical replicates.

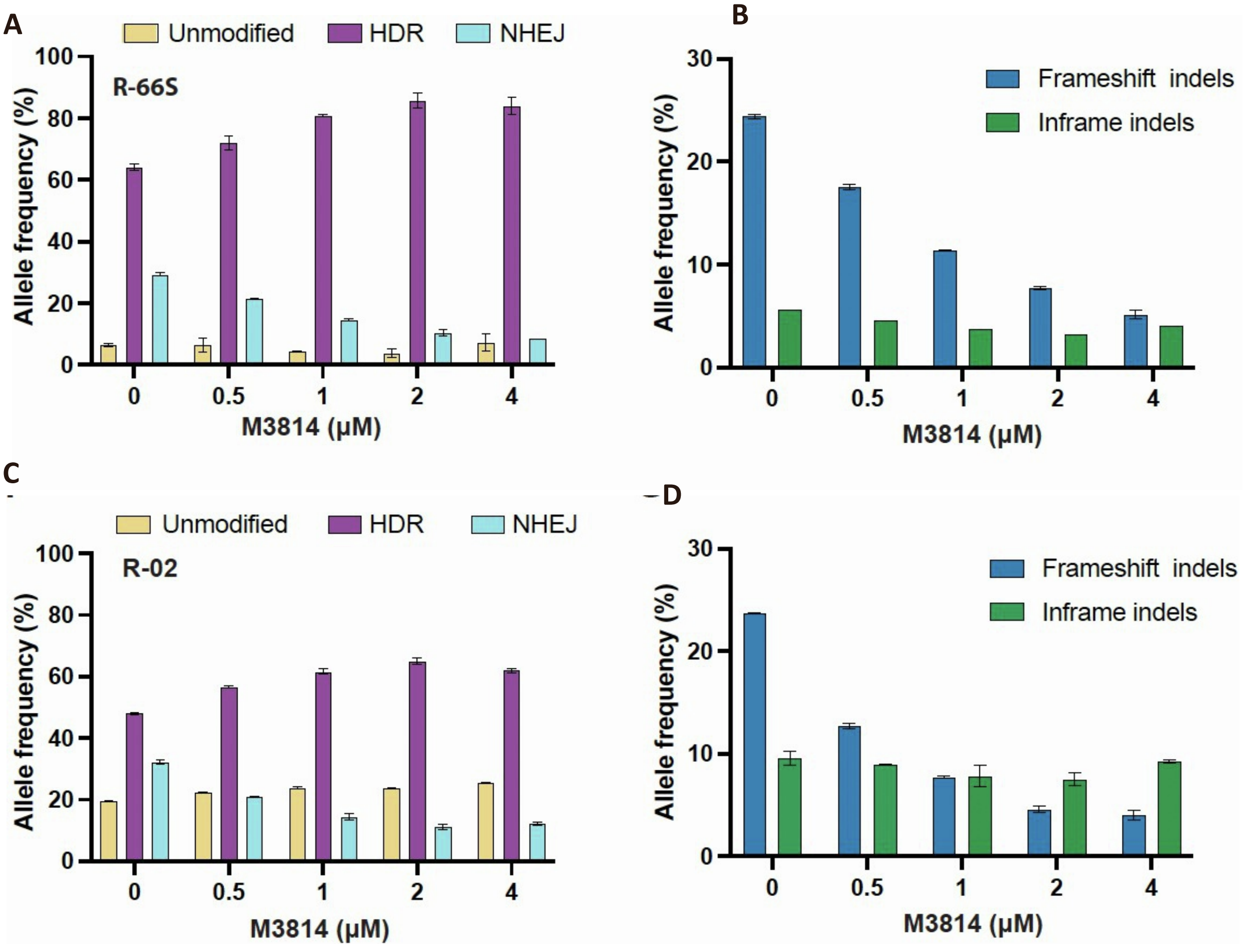


**Figure S26. M3814 results in a dose-dependent increase in HDR in patient-derived HSPCs. (A)** NGS analysis of SCD HSPCs edited with R-66S RNP + ssODN and treated with M3814 confirmed a dose-dependent increase in HDR and a decrease in NHEJ. After delivering R-66S + ssODN and incubating the cells with M3814 for 24 hours, HDR increased dose-dependently from 64.2 ± 1% without M3814 to 84 ± 2.9% with 4 µM M3814. However, no significant difference in HDR was observed between 2 and 4 µM, likely because initial toxicity at the higher dose affected cell cycle and repair. **(B)** A dose-dependent decrease in frameshift indels was observed in SCD HSPCs after R-66S+ssODN editing. The frequency of in-frame indels remained relatively steady, likely due to MMEJ-mediated repair of major in-frame indels, such as the 12bp deletion. **(C, D)** A similar dose-dependent increase in HDR and a reduction in frameshift indels were observed with R-02 + ssODN in SCD HSPCs. The R-02 gRNA generates MMEJ-mediated 9bp deletion as a major indel and a higher percentage of in-frame indels compared to R-66S.

**A**

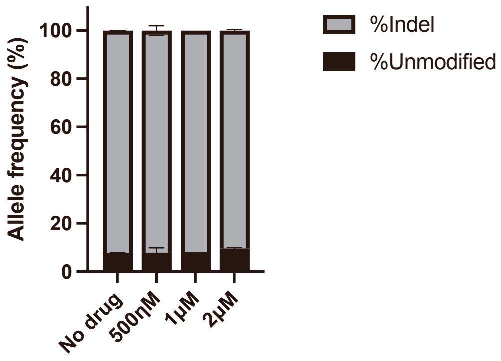


**B**

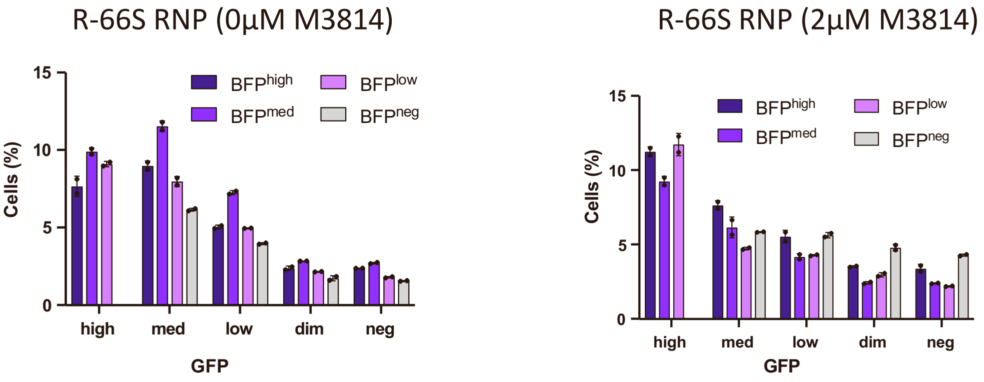


**Figure S27. Percentage of cells in each cluster based on GFP and BFP MFI after R-66S RNP editing with and without M3814 incubation for each independent replicate. (A)** Efficient editing seen SHD$^{GFP/BFP.}$ Cells were electroporated with R-66S RNP and immediately placed in media containing varying concentrations of M3814. After 24 hours of exposure, the drug was removed, and the cells were cultured for an additional 72 hours until editing was complete. **(B)** Cell distribution for two replicates is shown (independent electroporation) with SD error bars for cells treated with or without 2μM of M3814 after R-66S RNP editing.

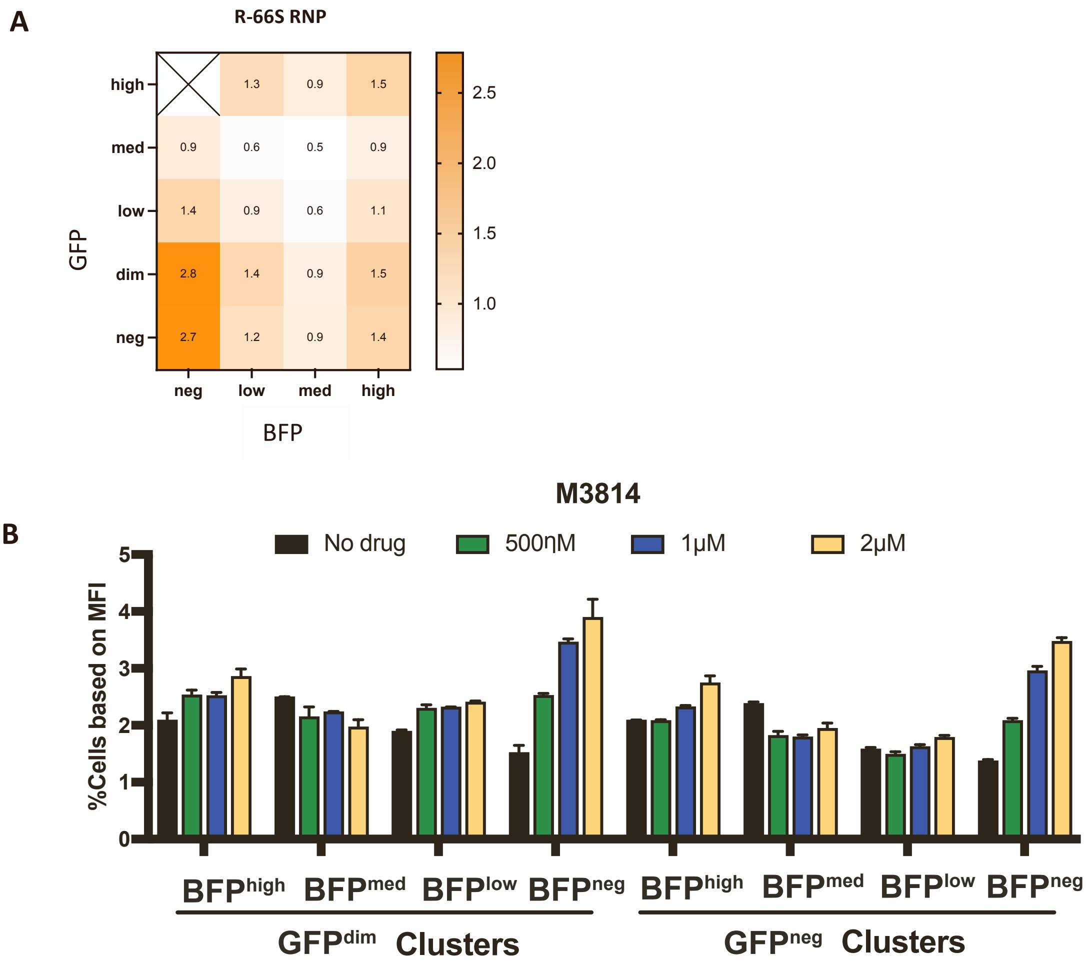


**Figure S28 M3814 HDR enhancer results in a dose-dependent increase in large deletions in RNP-treated SHD$^{GFP/BFP}$. (A)** The percentage of cells in each previously defined cluster based on GFP and BFP MFI was determined, and the fold difference between RNP-only edited cells incubated with or without 2μM of M3814 was calculated. M3814 resulted in a dose-dependent decrease in intermediate clusters, enriched in frameshift alleles mediated by NHEJ, similar to results seen in R-66S+ssODN conditions. However, a significant increase in GFP$^{dim}$/BFP$^{neg}$ (p=0.0062) and GFP$^{neg}$BFP$^{neg}$ cells (p=0.0049) was seen. **(B)** M3814 treatment also resulted in a dose-dependent relative increase in clusters enriched for large deletions, mediated via MMEJ and larger gene modifications or LOA, as evidenced by a significant dose-dependent increase in the percentage of GFP$^{dim}$/BFP$^{neg}$ and GFP$^{neg}$/BFP$^{neg}$ cells.

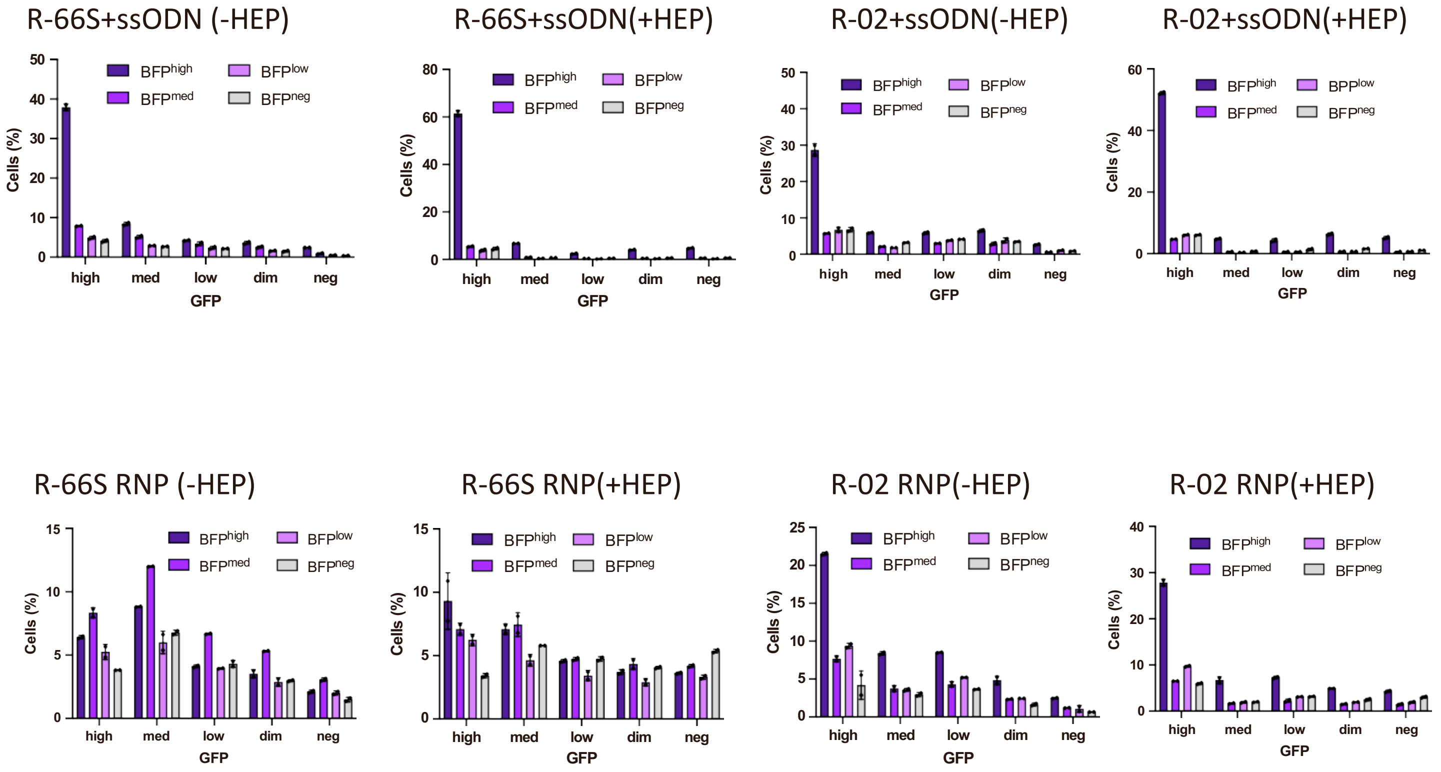


**Figure S29. Percentages of cells in each individual cluster after therapeutic R-02 and R-66S editing in SHD$^{GFP/BFP}$ with or without the addition of 25 µM of HEP.** Percentage of cells in each previously defined 20 clusters determined after 4 days of erythroid differentiation for each condition: n=2 individual replicates shown with SD error bars.

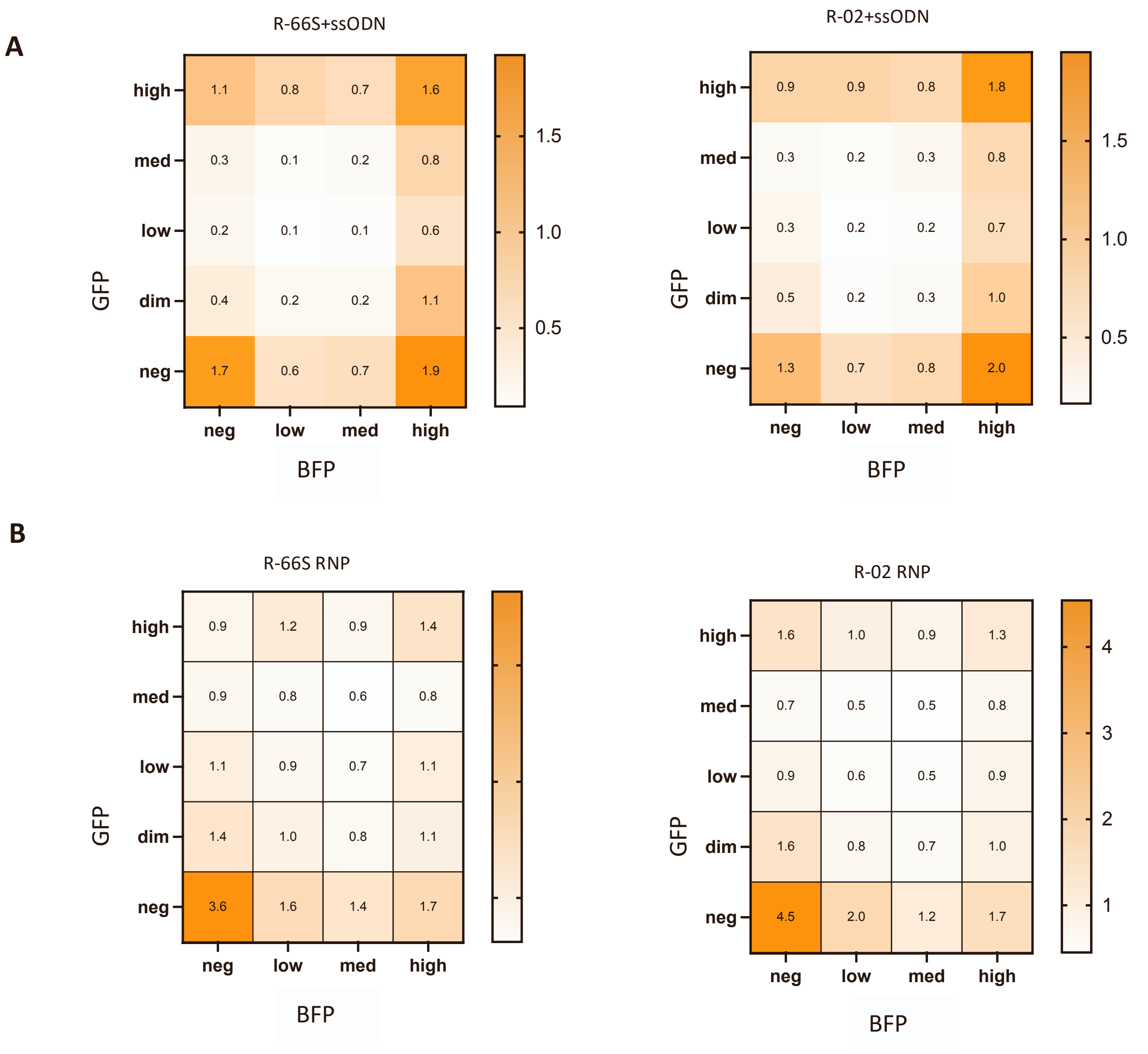


**Figure S30. Promoting end resection with HEP results in an increase in HDR in the presence of a corrective donor, but also a significant increase in LOA rates in RNP only treated SHD$^{GFP/BFP}$. (A)**The percentage of cells in each previously defined cluster based on GFP and BFP MFI was determined, and the fold difference between RNP+ssODN with or without the presence of 25 µM of HEP was calculated. An increase in the percentage of GFP$^{high}$BFP$^{high}$ cells was seen with HEP treatment, consistent with an increase in HDR rates. However, although there was no significant increase in the percentages of GFP$^{neg}$BFP$^{neg}$ and GFP$^{neg}$BFP$^{high}$ in RNP+ssODN-edited cells, this raises concern that an increase in LD and LOA events is observed alongside HDR enhancement with HEP treatment. **(B)** The percentage of cells in each cluster with and without HEP treatment after RNP-only editing was then examined. Without the addition of the ssODN corrective donor, a significant fold increase in GFP$^{neg}$BFP$^{neg}$ cells was observed for both R-66S and R-02 RNP (p=0.002, p=0.04, respectively).

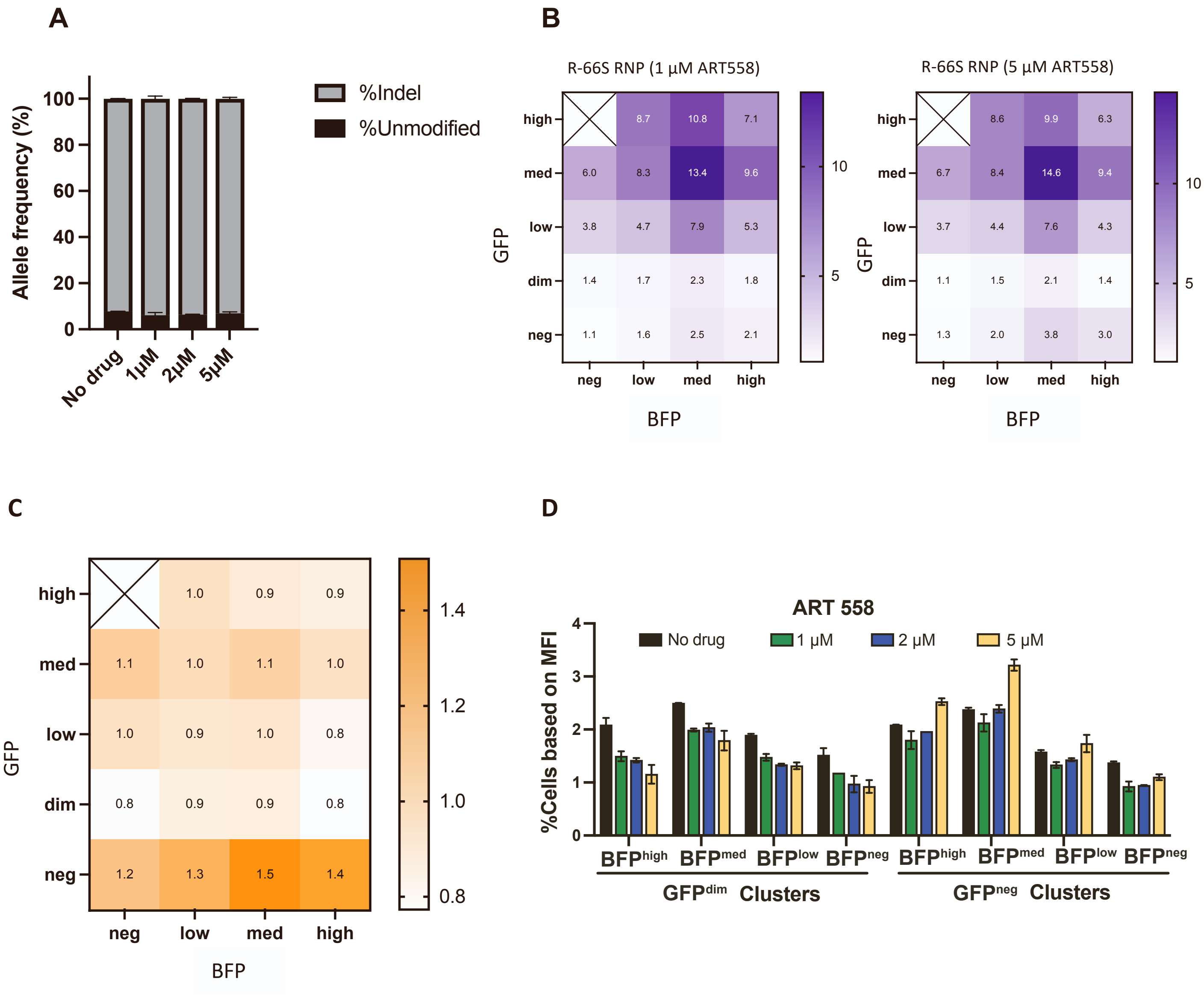


**Figure S31. ART558, a polymerase theta inhibitor, inhibits MMEJ and reduces large deletions in RNP-treated SHDGFP/BFP, but increases the rate of LOA. (A)** Efficient editing seen $SHD^{GFP/BFP.}$ Cells were electroporated with R-66S RNP and immediately placed in media containing varying concentrations of ART558. After 24 hours of exposure, the drug was removed, and the cells were cultured for an additional 72 hours until editing was complete. **(B)** The percentage of cells in the previously defined 20 clusters was analyzed by flow cytometry, using changes in GFP and BFP MFI. Treatment with ART558 results in a dose-dependent reduction in $GFP^{dim}$ clusters, where large deletions are repaired via MMEJ. Conversely, a dose-dependent increase in $GFP^{neg}$ clusters indicates that MMEJ does not mediate LOA or large gene modification events. **(C)** Relative fold change in % of cells in cells treated with 1 µM of vs 5µM of ART is shown, and a clear increase is seen in the percentage of $GFP^{neg}$ cells. **(D)** MMEJ inhibition (ART 558) increases the percentage of cells carrying larger gene modifications. Treatment with ART558 results in a dose-dependent reduction in $GFP^{dim}$ clusters, where large deletions are repaired via MMEJ. Conversely, a dose-dependent increase in $GFP^{neg}$ clusters indicates MMEJ does not mediate LOA events.
