# Supplementary material for: Complex HBB gene editing outcomes revealed by a fluorescent reporter cell model
Source: Mol Ther Nucleic Acids. 2026 Feb 3;37(1):102854. doi: 10.1016/j.omtn.2026.102854 (PMC12926567; doi:10.1016/j.omtn.2026.102854)
Supplement: Document S2. Article plus supplemental information [file mmc2.pdf]

Molecular Therapy
Nucleic Acids
Original Article

Complex HBB gene editing outcomes revealed by a fluorescent reporter cell model

Cecile L. Karsenty,[1] Daniel Betancourth,[2] Mingming Cao,[2] Quoc-Khanh Pham,[2] So Hyun Park,[2] and Gang Bao[2]

[1]Division Hematology/Oncology, Department of Pediatrics, Baylor College of Medicine, Cancer and Hematology Center, Texas Children's Hospital, Houston, TX 77030, USA; [2]Department of Bioengineering, Rice University, Houston, TX 77030, USA

ABSTRACT

CRISPR-Cas9 gene editing offers the potential to transform the treatment of sickle cell disease by correcting the sickle mutation in β-globin gene (*HBB*). However, in addition to alleles with homology-directed repair (HDR), Cas9 editing at *HBB* generates a diverse spectrum of outcomes, including small insertions and deletions (indels), large deletions (LDs), and loss of allele (LOA) events, that can compromise genomic integrity and raise significant safety concerns. While new pharmacological modulators have been developed to increase the HDR rates, they may also elevate the risk of large gene modifications. To better understand the complex *HBB* gene editing outcomes, we engineered a live-cell, dual-fluorescent reporter cell model enabling allele-specific monitoring of *HBB* expression via GFP and blue fluorescent protein (BFP) tagging. Based on fluorescence intensities, this model can discriminate in-frame alleles, frameshift indels, LDs, and LOA, supporting high-throughput genotype-phenotype mapping. By applying HDR-enhancing agents, we further show that the reporter sensitively captures shifts in LOA outcomes that are missed by other bulk assays. This cell model provides a valuable tool for dissecting gene editing outcomes due to different DNA repair pathways and quantitatively linking editing genotypes to erythroid phenotypes and thus can be used to evaluate the safety of CRISPR/Cas9-based therapies.

INTRODUCTION

Sickle cell disease (SCD) is one of the most prevalent and severe inherited monogenic disorders, caused by a point mutation in the β-globin gene (*HBB*) that results in hemoglobin S (HbS). SCD affects over 7 million individuals globally, causing significant morbidity, premature mortality, and healthcare burden.[1] While matched-donor allogeneic hematopoietic stem cell transplant (HSCT) offers a curative treatment, this option is available only to less than 15% of individuals with SCD.[2,3] Thus, there has been significant momentum in recent years toward the development of autologous gene therapies. In late 2023, the Food and Drug Administration (FDA) approved Casgevy, the first CRISPR/Cas9-based autologous gene editing therapy for SCD.[4] This therapy aims to reverse the SCD phenotype by inducing fetal hemoglobin (HbF) through disruption of the *BCL11A* erythroid enhancer.[4–6] Alternatively, CRISPR/Cas9-based gene editing can correct the sickle mutation in patient-derived hematopoietic stem and progenitor cells (HSPCs).[7] In this approach, ribonucleoproteins (RNPs) formed by Cas9 nuclease and CRISPR guide RNA (gRNA) targeting the *HBB* gene are delivered into SCD HSPCs together with a corrective donor template, either packaged in adeno-associated virus vector 6 (AAV6)[8] or as a single-stranded oligonucleotide (ssODN).[9–11] Clinical trials using the gene correction approach targeting a locus near the sickle mutation with gRNA R-02[12] are currently underway (NCT04774536/NCT04819841).

Cas9-induced double-strand breaks (DSBs) generate a wide spectrum of non-HDR repair outcomes, including small insertions and deletions (indels), large deletions (LDs), and complete loss of allele (LOA). However, analyses of genome editing outcomes in SCD HSPCs have largely centered on quantifying small NHEJ-mediated indels and HDR events, whereas larger structural modifications often arising through microhomology-mediated end joining (MMEJ)[13,14] have not been carefully analyzed, and their phenotypic consequences remain poorly understood. Our prior long-read single-molecule real-time sequencing with dual unique molecular identifiers (SMRT-seq with dual UMI) revealed a high frequency and broad spectrum of LDs induced by R-02 and R-66S gRNA targeting the sickle mutation. These LD events decreased but remained appreciable when an ssODN donor was added.[15] Although enrichment of HDR-repaired alleles with concomitant depletion of indel-bearing cells has been reported during erythroid maturation in a xenograft mouse,[9] depletion of indels in peripheral blood does not equate to safety. If a substantial fraction of edited bone marrow progenitors fail to mature into red blood cells (RBCs) and instead undergo ineffective erythropoiesis, this could impose marrow stress and hyperplasia,[16] while leaving behind long-lived HSCs harboring diverse indels and large structural modifications, the risks of which are not fully understood. A recent clinical trial using R-02 RNP and AAV6

Received 19 June 2025; accepted 30 January 2026;
https://doi.org/10.1016/j.omtn.2026.102854.

Correspondence: So Hyun Park, Department of Bioengineering, Rice University, Houston, TX 77030, USA.
E-mail: sp44@rice.edu
Correspondence: Gang Bao, Department of Bioengineering, Rice University, Houston, TX 77030, USA.
E-mail: gang.bao@rice.edu

© 2026 The Author(s).
Published by Elsevier Inc. on behalf of The American Society of Gene and Cell Therapy.
This is an open access article under the CC BY license (http://creativecommons.org/licenses/by/4.0/).

donor for sickle mutation correction further illustrates this complexity. The treated patient exhibited a striking post-infusion shift in editing outcomes: indel frequency increased from 21.4% to 92.6%, while HDR-corrected alleles fell from 33.1% to 1.3% in peripheral blood CD15$^+$ cells 1 year after infusion.[17] Despite this, the patient demonstrated a favorable hemoglobin profile (<5% HbS and >78% HbF), suggesting that therapeutic benefit arose not from direct correction of the sickle mutation, but from HbF induction associated with indel alleles. The mechanism underlying this unexpected HbF upregulation and the apparent selective expansion of indel-bearing cells remains unresolved. Collectively, these observations highlight the complexity of *HBB* repair and the need to define how specific non-HDR genotypes influence *HBB* expression, HbF induction, and cell fitness throughout erythroid differentiation, as well as their impact on long-term persistence and the potential risk of clonal expansion.

To better understand the complex *HBB* editing outcomes, we developed a dual-fluorescent *HBB* reporter in sickle human-umbilical-cord-derived progenitor erythroid 2 (HUDEP-2) cells[18–20] that enables high-resolution genotyping and phenotyping of Cas9 edits. By converting fluorescence intensity states into ∼20 genotype classes, this model distinguishes allele-specific in-frame edits, frameshift indels that introduce early versus late nonsense (NS) mutations, and LD versus LOA, defined as larger aberrations that exceed several kilobases and fail to amplify by 6-kb long-range PCR. These genotypes are simultaneously linked to *HBB* expression, HbF induction, cell fitness, and erythroid differentiation. Because cells remain viable, the platform supports prospective isolation, time course analysis, and testing of HDR-enhancing interventions, providing an integrated readout of both benefit (HDR gain) and risk (genome-altering byproducts). We show that our reporter cell model (1) maps fine-scale *HBB* genotypes, including rare classes such as LD and LOA that are difficult to resolve by next-generation sequencing (NGS), long-read sequencing, or droplet digital PCR (ddPCR) alone; (2) quantifies HbF induction and apoptosis associated with distinct editing outcomes; (3) provides a practical, scalable testbed to evaluate emerging HDR-enhancement strategies for both efficacy and safety; and (4) enables mechanistic dissection of repair pathways leading to LOA, informing strategies to minimize these events. Thus, this model offers a valuable tool for dissecting DNA repair outcomes at *HBB*, quantitatively linking editing genotypes to erythroid phenotypes and evaluating the safety of CRISPR/Cas9-based therapies.

RESULTS

On-target gene editing at *HBB* results in ineffective erythropoiesis in SCD HSPCs

Because HDR is inefficient in HSCs,[21,22] Cas9 editing at the sickle locus produces a mixture of repair outcomes, including indels and larger disruptive events such as LDs[23] and LOA.[24] Prior studies have shown that these non-HDR alleles are common and can differentially persist during erythropoiesis.[10] Together with recent clinical observations suggesting unexpected expansion of certain indel-bearing clones,[17] these findings underscore the need to define how individual *HBB* genotypes influence β-globin expression, HbF induction, and erythroid maturation. To investigate these effects, CD34$^+$ HSPCs from three SCD donors (donors 1–3) were electroporated with HiFi SpCas9 RNPs targeting *HBB* exon 1 (R-66S or R-02),[10,12] with or without an ssODN donor correcting the sickle mutation[10,25,26] (Figure 1A). We quantified large gene modification events using a ddPCR allelic drop-off assay, which measures the fraction of *HBB* alleles that are amplifiable by short-range ddPCR. Alleles that fail to amplify are inferred to harbor CRISPR-induced large gene modifications, including LDs, large insertions, chromosomal rearrangements, chromosomal truncations, or chromosomal loss. Because LD is the predominant contributor to allelic drop-off, these events are referred to as "LD" in ddPCR figures throughout the paper. NGS detected HDR and small indels in the remaining intact alleles, and NGS data were normalized to ddPCR-derived total allele counts to report true allelic fractions across all *HBB* copies.[15] Both gRNAs achieved high overall editing efficiencies. Without ssODN, R-66S generated a higher proportion of frameshift indels than in-frame indels, whereas R-02 predominantly produced a 9-bp in-frame deletion via MMEJ (Figure S1), resulting in more in-frame than frameshift indels (Figure 1B). R-66S also induced more LDs, 23.8% (5.2%), than R-02, 17.4% (2.2%). Adding ssODN shifted repair toward HDR, reaching 46% (0.97%) for R-66S and 30.8% (0.05%) for R-02,[10,21,25] and correspondingly reduced both in-frame indels and LDs, with a smaller decrease in frameshift indels (Figure 1B).[15,27–30]

Erythroid differentiation assays revealed a pronounced maturation delay following RNP-only editing with either gRNA (Figure 1C). This delay was evident as an increased proportion of CD36$^+$/CD235a$^+$ immature cells on day 15 (donor 1) or day 13 (donor 2) of differentiation[31] (Figures S2 and S3), with a similar pattern observed using CD71$^+$/CD235a$^+$ markers (Figure S4). Addition of ssODN restored differentiation to near-mock levels. In a third donor, long amplicon sequencing (LongAmp-seq)[16] was performed on sorted immature and mature fractions after 10 days of differentiation, showing frameshift indels were enriched in the immature fraction, whereas in-frame indels and unmodified alleles were enriched in the mature fraction, implicating frameshift indels in the observed maturation delay (Figure 1D; Figure S5). HPLC analysis, which reports the relative fractions of each hemoglobin species, showed that R-02 RNP editing reduced HbS from 96.5% in mock to 34.5% and produced 26% HbA via HBD conversion,[10] with 40% HbF. With ssODN, HbA increased to an average of 61% (R-66S) and 53% (R-02), and HbF, although lower than in RNP-only samples, remained above mock (Figures S6 and S7). While HPLC does not quantify absolute hemoglobin per cell, the changes in relative hemoglobin composition align with the maturation phenotypes. RNP-only editing yields HbS knockout with limited HbA restoration and only partial HbF compensation, and these samples show impaired erythroid maturation compared with mock. In contrast, donor-mediated HDR produces a more favorable hemoglobin profile and restores maturation toward mock levels (Figure 1C).

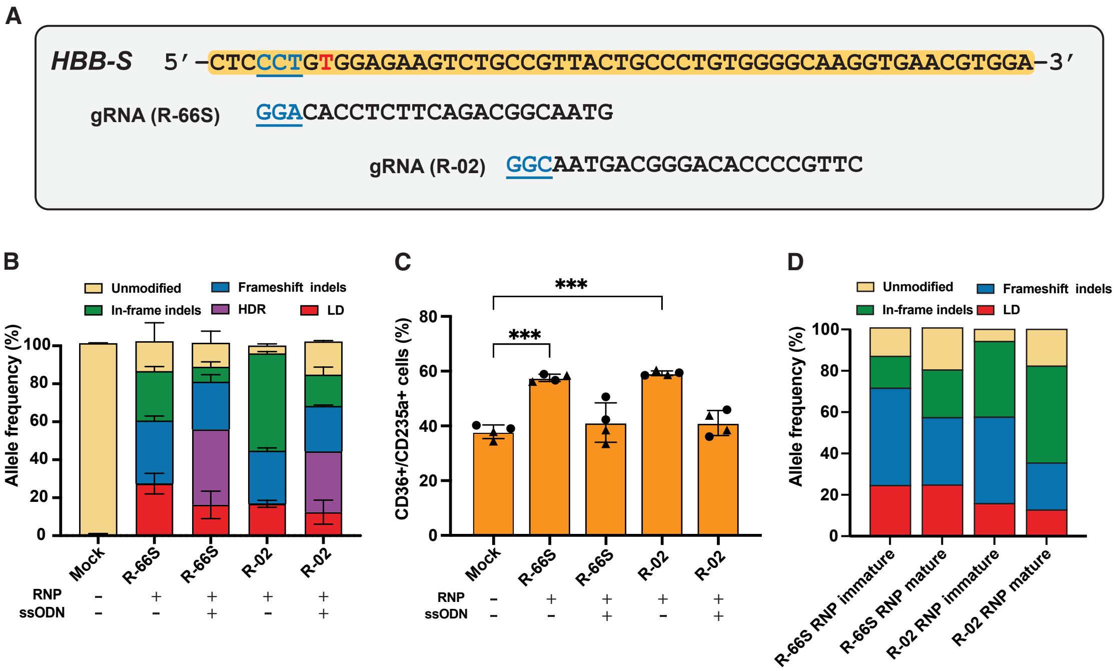

Figure 1. CRISPR editing of *HBB* delays erythroid maturation in HSPCs from SCD patients

Sickle-patient-derived CD34+ HSPCs from two donors were edited at *HBB* using HiFi SpCas9 RNPs with sgRNAs R-66S or R-02, delivered by electroporation ± ssODN donor. (A) *HBB* exon 1 sequence showing the sickle mutation (red). Protospacers for R-66S and R-02 are shown with PAMs underlined (blue). (B) Allele frequency quantified by NGS and ddPCR. ddPCR measured allelic-drop off as a surrogate for large deletion (LD) resulting from loss of the primer/probe binding site, while NGS quantified HDR and small indels on intact alleles. NGS values were normalized to ddPCR to true allelic fractions across all *HBB* alleles (intact and drop-off). Efficient editing was achieved with R-66S and R-02 in $n = 2$ biological replicates (independent donors), each with two technical replicates (independent electroporation). (C) Erythroid maturation assays showed a significant delay after R-66S or R-02 RNP versus mock ($p < 0.0001$ and $p = 0.0001$, respecitvely using a 2-sided Welch t test, evidenced by increased CD36+/CD235a+ immature cells in donor 1 (15-day differentiation) and donor 2 (13-day differentiation). Adding the ssODN donor restored maturation to near-mock levels. (D) In a third donor, edited cells were differentiated for 10 days, sorted into immature and mature fractions, and allele frequencies were measured by LongAmp-seq. Across both gRNAs, frameshift indels were enriched in the immature fraction, whereas in-frame indels and unmodified alleles were enriched in the mature fraction, implicating frameshift indels in the observed maturation delay ($n = 1$ experiment).

Despite maturation patterns resembling mock (Figure 1C), bulk RNP+ssODN-edited cultures still contain a substantial fraction of cells with disruptive genotypes that are not sensitively captured by the population-level maturation assay. In our prior clonal genotyping, ∼20% of R-66S RNP+ssODN-edited colonies were complete *HBB* knockouts resulting from frameshift indels and/or LDs on both alleles.[15] Mechanistic dissection of such heterogeneous genotypes is difficult in primary SCD HSPCs, which have a finite *ex vivo* lifespan after CD34+ isolation and rapidly lose self-renewal capacity, limiting longitudinal tracking, multiple perturbations, and genotype-phenotype correlation within the same culture. To address these constraints, we developed a fluorescent reporter model that enables controlled, allele-resolved analysis of editing outcomes and their functional consequences in a stable, scalable cell system.

Establishment of S-HUDEP2$^{GFP/BFP}$ dual-fluorescent model for allele-specific monitoring of *HBB* editing

We selected HUDEP-2 cells to introduce the *HBB* reporter because they are a well-characterized human erythroid model that recapitulates early and intermediate erythropoiesis and adult β-globin expression, while remaining highly amenable to precise genome editing.[18–20] HUDEP-2 cells are broadly used as a first-line platform to benchmark CRISPR strategies before validation in primary HSPCs,[32,33] dissect mechanisms of HbF induction,[34] and identify new HbF regulators.[35–37] Their robust expansion, uniform differentiation, and reproducible β-globin output provide an ideal setting for a *HBB*-allele-specific fluorescent reporter, enabling mechanistic genotype-phenotype studies that are impractical in primary HSPCs.

We engineered a dual-fluorescent sickle HUDEP2 cell model (SHD$^{GFP/BFP}$) from the parental sickle HUDEP2 (SHD) line,[18–20] which carries a homozygous sickle mutation, by introducing a P2A-GFP-pA cassette at the *HBB* C-terminus via HDR, generating a biallelic SHD$^{GFP/GFP}$ clone. One GFP allele was subsequently converted to BFP through a Y66H substitution (TAC→CAT) using Cas9 RNP and ssODN,[38] and dual-positive cells were sorted to establish the stable SHD$^{GFP/BFP}$ line. Long-read sequencing confirmed allele-specific tagging of *HBB* with GFP and BFP, enabling direct visualization of expression from each allele (Figure 2A). HPLC, following 7 days of erythroid differentiation, detected a single HbS

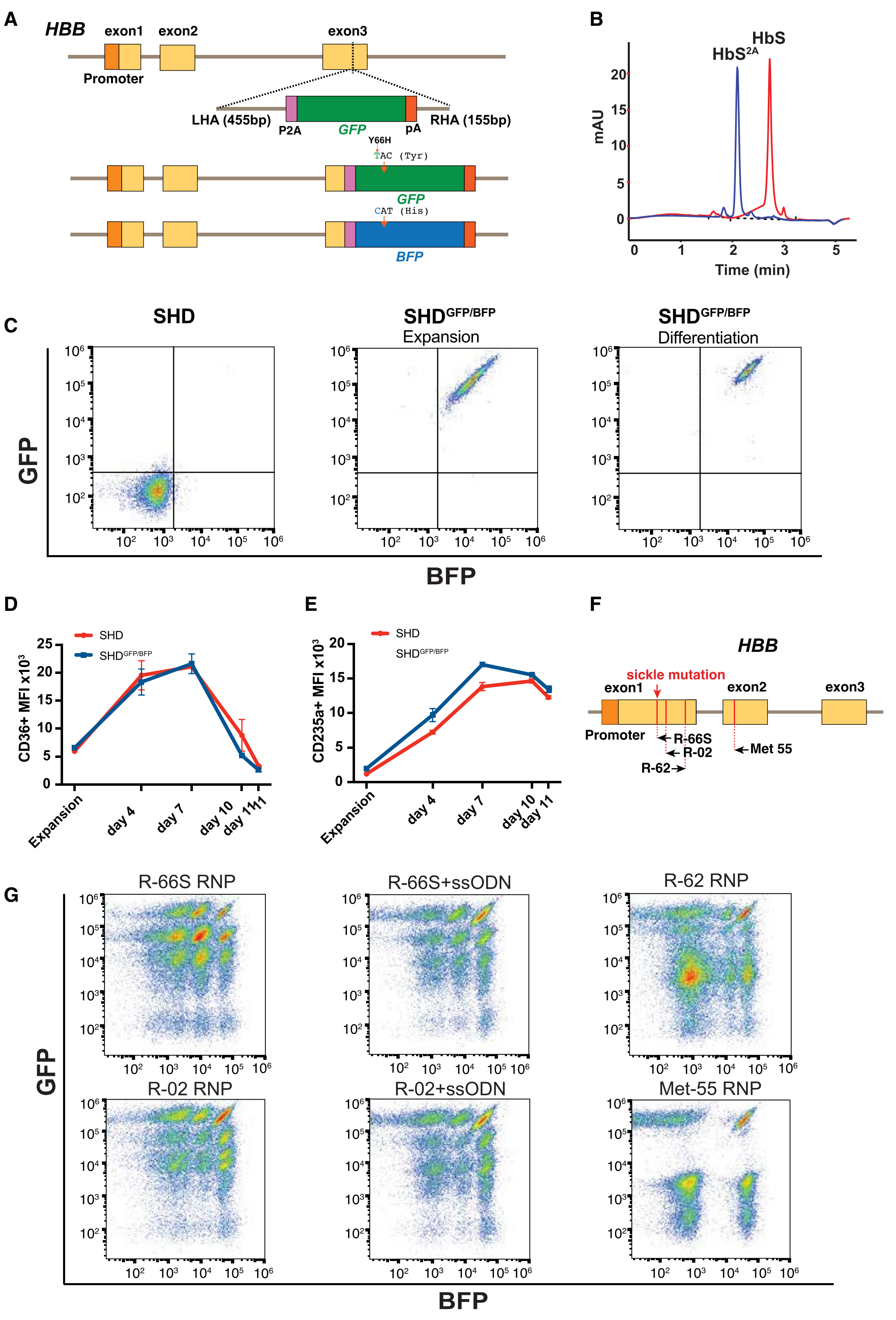

(legend on next page)

peak in the parental SHD line, whereas $SHD^{GFP/BFP}$ showed a single earlier-eluting peak corresponding to HbS^{2A} (Figure 2B). Flow cytometry demonstrated stable co-expression of GFP and BFP during expansion and a strong increase in fluorescence intensity during erythroid differentiation, consistent with activation of the endogenous *HBB* promoter (Figure 2C). $SHD^{GFP/BFP}$ cells displayed maturation kinetics, as measured by CD36 and CD235a expression (Figures 2D and 2E) and maintained viability comparable to the parental SHD line (Figure S8), confirming that reporter tagging did not impair erythroid differentiation capacity.

$SHD^{GFP/BFP}$ reports gRNA-specific editing outcomes for gRNAs targeting *HBB* exons 1 and 2

We evaluated four gRNAs spanning *HBB* exons 1–2 to probe how target position and indel spectra shape $SHD^{GFP/BFP}$ fluorescence. Two guides (R-66S and R-02) are the clinically relevant early exon-1-targeting gRNAs used for sickle-mutation correction; we also tested R-62 targeting distal exon 1 and Met-55 targeting the alternative start codon in exon 2 (Figure 2F).[39] All gRNAs edited efficiently and produced distinct indel profiles (Figures S9 and S10). Prior studies show that a positional boundary bisects *HBB* exon 1: nonsense mutations (NSs) in the 5′ portion largely escape nonsense-mediated decay (NMD), whereas more distal NS trigger efficient NMD, with mRNA levels declining gradually up to NS23 and dropping sharply by NS26.[40,41] We therefore reasoned that gRNAs targeting early exon 1 and creating an NS across this boundary would generate a range of *HBB* mRNA abundances and, thus, a spectrum of GFP/BFP mean fluorescent intensities (MFIs) in $SHD^{GFP/BFP}$. Consistent with this, R-66S and R-02 produced the broadest range of GFP/BFP clusters, including intermediate MFIs indicative of partial NMD escape, whereas R-62 yielded fewer intermediate clusters, and Met-55 largely eliminated them. For R-66S and R-02, addition of an ssODN increased the $GFP^{high}BFP^{high}$ fraction, consistent with HDR enrichment (Figure 2G).

Optimization of $SHD^{GFP/BFP}$ for cluster-resolved *HBB* editing analysis

We validated and refined the $SHD^{GFP/BFP}$ reporter cell model by longitudinally profiling R-66S-RNP-edited cells during expansion and on differentiation days 4, 8, and 11. Cluster separation was stable on days 4 and 8 but blurred by day 11, establishing days 4–8 as the optimal window for sorting and genotype assignment (Figure S11). Using single-color SHD controls (SHD^{GFP} and SHD^{BFP}) for compensation, we defined a 4 × 5 grid of GFP/BFP clusters based on MFI (Figure S12). Although we anticipated symmetric behavior of the two alleles, we resolved five GFP-defined clusters but only four for BFP. This asymmetry likely reflects GFP's higher brightness and spectral spillover,[42] which collapses BFP^{dim} and BFP^{neg}. Future iterations replacing BFP with a brighter, spectrally distinct fluorophore (e.g., mCherry) should further improve cluster separation and genotype calling. Post-sorting, clusters retained their characteristic GFP/BFP MFIs after additional differentiation, confirming fluorescence stability and sorting fidelity (Figure S13).

Throughout the manuscript, we summarized % cells, allele frequencies, and cluster-level phenotypes as 4 × 5 heatmaps that preserve the spatial layout of the GFP-BFP flow cytometry plots. This format enables rapid visual mapping of cluster identity (GFP/BFP MFI), genotype class (in-frame, frameshift, LD, and LOA), and phenotype (%HbF, %Annexin V^+), with proportions encoded by color. Main-text heatmaps display means from $n = 2$ replicates; companion supplemental figures provide per-replicate values with SD error bars.

$SHD^{GFP/BFP}$ reports allele-specific *HBB* genotypes and expression states in live cells

Using R-66S and R-02 gRNAs, we isolated each fluorescence-defined cluster to identify the genotypes underlying its characteristic GFP/BFP MFI profile. In $SHD^{GFP/BFP}$ cells edited with R-66S or R-02 ± ssODN, NGS and ddPCR confirmed efficient editing for both guides (Figure 3A), following the same trends observed in SCD HSPCs (Figure 1A) and prior reports.[10,11] After 4 days of erythroid differentiation, GFP/BFP flow cytometry resolved 20 clusters per gRNA, with the percentage of cells in each cluster shown as heatmaps (Figures 3B and 3C). With RNP alone, R-66S, whose edits are more heterogeneous and frameshift skewed, yielded fewer $GFP^{high}BFP^{high}$ cells (4.2%) (Figure 3B) than R-02 (19.5%) (Figure 3C), reflecting R-02's predominant 9-bp MMEJ deletion.

Figure 2. Generation and validation of sickle HUDEP2 dual fluorescent model for allele-specific monitoring of *HBB* expression

(A) The $SHD^{GFP/BFP}$ model was generated from S-HUDEP2 cells by delivering Cas9 RNPs together with a double-stranded DNA donor targeting the *HBB* C-terminus. The donor contained left and right homology arms, a P2A-GFP-poly(A) tail, labeling each *HBB* allele via HDR. A biallelic $SHD^{GFP/GFP}$ clone was then converted to a $SHD^{GFP/BFP}$ hetero-allelic line by introducing a Y66H substitution via RNP + ssODN to convert one GFP allele to BFP. Dual-positive cells were sorted to establish the clonal $SHD^{GFP/BFP}$ line. Long-read sequencing confirmed allele-specific labeling of *HBB* with GFP and BFP. (B) Hemoglobin HPLC after 7 days of differentiation showed a single HbS peak in parental SHD, whereas $SHD^{GFP/BFP}$ showed a single peak at a shorter retention time, consistent with addition of the 21-amino acid 2A tail to HbS after P2A cleavage (HbS^{2A}), which increases the protein's negative charge. (C) The left panel shows parental SHD used for negative gating. The middle panel shows $SHD^{GFP/BFP}$ cells with stable co-expression during expansion, and the right panel shows increased GFP and BFP mean fluorescent intensities (MFIs) by day 4 of erythroid differentiation, consistent with regulation by the endogenous *HBB* promoter, whose activity rises during erythropoiesis. (D and E) SHD $^{GFP/BFP}$ maintained erythroid maturation patterns comparable to the SHD, as measured by CD36 (D) and CD235a (E) expression over time. Flow cytometry values are the median MFI within CD36+ or CD235a+ gates (mean ± SD; $n = 2$ replicates). (F) Schematic of *HBB* exons 1–3 showing the positions of four gRNAs tested in the $SHD^{GFP/BFP}$: R-66S and R-02 gRNAs targeting early exon 1, R-62 targeting distal exon 1, and Met-55 targeting the alternative start codon in early exon 2. (G) $SHD^{GFP/BFP}$ was edited with four gRNAs; for R-66S and R-02, edits were also performed with ssODN. Efficient editing was confirmed in bulk culture for all four gRNAs. Flow cytometry at day 4 of erythroid differentiation showed distinct fluorescent clusters for each gRNA. R-66S and R-02 generated the greatest cluster diversity. With ssODN, the $GFP^{high}BFP^{high}$ cluster encompassing in-frame HDR alleles increased, while non-HDR clusters decreased. R-62 produced fewer intermediate clusters and Met-55 eliminated intermediate populations.

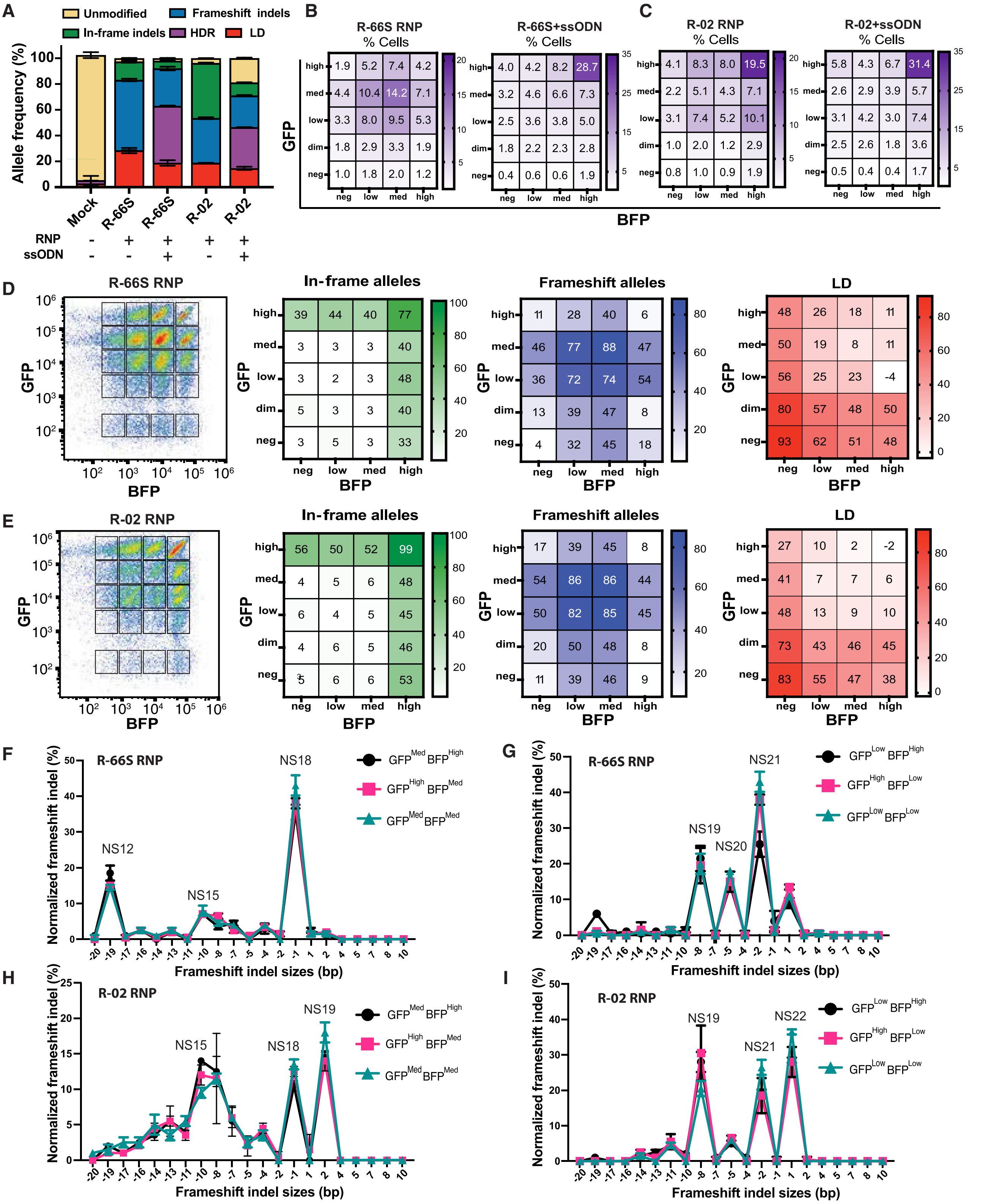

(legend on next page)

Adding ssODN decreased intermediate- and negative-clusters, and increased GFP^{high}BFP^{high}, consistent with enrichment for HDR/in-frame alleles. Fold changes in cluster percentages between RNP-only and RNP+ssODN are summarized in Figure S14. Next, we sorted all 20 fluorescence-defined clusters on days 4–5 of differentiation from each R-66S- or R-02-RNP-edited cells with replicates and profiled genotype composition by NGS and ddPCR (Figures 3D and 3E; Figure S15). The GFP^{high}BFP^{high} was composed largely of in-frame alleles that preserve *HBB* expression. Clusters with high GFP or BFP on one allele (top row/right column) contained ~50% in-frame alleles. Medium and low-MFI clusters were enriched for frameshift indels. GFP$^{low, mid}$BFP^{low,mid} clusters were dominated by biallelic frameshift genotypes, and clusters with one allele at medium or low MFI contained ~50% frameshift alleles. Dim/negative clusters were dominated by LDs, with GFP^{neg}BFP^{neg} cluster showing 93% LD for R-66S and 83% LD for R-02 and clusters with one dim or negative allele (GFP^{dim,neg} or BFP^{neg}) containing ~50% LD alleles.

Together, these data indicate that GFP/BFP fluorescence is a reliable proxy for the underlying *HBB* genotypes generated by editing, even though sorted clusters are not completely genotype-pure populations. Residual impurity likely stems from both the close spacing of adjacent GFP/BFP populations during sorting and the resolution limits of our genotyping bins. For example, although GFP^{high}BFP^{high} is expected to be enriched for in-frame alleles, the R-66S GFP^{high}BFP^{high} population contained 77% in-frame and 11% LDs. Long-read SMRT-seq showed that these LDs were predominantly <500 bp and 76% were in-frame, explaining the high MFI despite their classification as LDs. Similarly, the GFP^{low}BFP^{high} cluster in R-02-edited cells contains two visible subclusters (higher vs. lower GFP) that we analyzed together; resolving them separately would likely reveal distinct frameshift genotypes. We therefore suspect that the true underlying genotype composition of each cluster is cleaner than our current sorting and genotyping can fully resolve. Future refinements, such as tighter, cluster-centered gating and replacing BFP with a brighter, more spectrally distinct fluorophore, should further improve separation and purity.

Medium versus low MFI reflects frameshift-induced nonsense mutation position in *HBB* exon 1

Frameshift indels were enriched in both medium- and low-MFI clusters. Given the established relationship between NS position in *HBB* exon 1 and NMD efficiency,[40,41] we mapped each unique frameshift indel to its resulting NS codon position to determine whether differences in NS placement within *HBB* exon 1 explain the medium versus low MFI phenotypes. In Figures 3F–3I, the *x* axis represents the frameshift indel sizes. The "NS" labels shown above each peak indicate the codon position of the premature nonsense mutation generated by each specific indel size. For each indel, we reconstructed the *HBB* coding sequence and identified the premature stop codon introduced by the frameshift, thereby assigning an NS codon position to each indel. For R-66S gRNA, frameshift indels introducing NS before codon 19 (NS < 19) showed partial expression with medium MFI (Figure 3F), consistent with NMD bypass, whereas indels introducing NS at or after codon 19 (NS $\geq$ 19) triggered efficient NMD and resulted in low MFI (Figure 3G). R-02 showed the same overall pattern, but NS19 alleles were found in both the medium (Figure 3H) and low MFI (Figure 3I) clusters, arising from distinct indel sizes (a 2-bp insertion in the medium cluster and an 8-bp deletion in the low cluster). This likely reflects context-dependent effects: different indels generate distinct amino acid sequences despite sharing the same stop codon position, leading to differences in MFI.

Allele-resolved long-read SMRT-seq reveals extensive LOA in GFP-negative clusters

To resolve structural events flagged as LD by the ddPCR drop-off assay, we performed long-read SMRT-seq analysis on 19 sorted clusters (all except GFP^{high}BFP^{neg}) from R-66S-RNP-edited SHD$^{GFP/BFP}$(Figure S13). A ~6 kb region spanning the R-66S cut site and the Y66H marker was PCR amplified and analyzed on PacBio SMRT-seq.[43] This approach distinguished GFP vs. BFP alleles and quantified allele-specific LD or LOA (Figure 4A). Deletions extending past Y66H yet remained amplifiable were counted as "Loss of Y66H reads," whereas non-amplifying alleles were classified as "LOA." Untreated cells (UT) showed a 1:1 GFP:BFP read

Figure 3. SHD$^{GFP/BFP}$ model reports on allele-specific *HBB* genotypes and expression in live cells
(A) SHD$^{GFP/BFP}$ were edited with R-66S and R-02, delivered as RNP or RNP + ssODN. Efficient editing was achieved for both guides. NGS quantified HDR, in-frame, and frameshift indels; ddPCR quantified LDs. R-66S yielded higher frameshift and LD rates than R-02. (B and C) After 4 days of erythroid differentiation, identical GFP/BFP-MFI gating resolved 20 clusters for R-66S (B) and R-02 (C). The percentage of cells in each cluster is displayed as a 4 × 5 heatmap, with the spatial arrangement matching that of the flow cytometry plots. Cluster distributions reflected gRNA-specific outcomes. Addition of ssODN reduced the proportion of intermediate/negative clusters in most cases and increased the GFP^{high}BFP^{high} population, corresponding to HDR or in-frame alleles. R-66S (D) or R-02 (E) RNP-treated cells were bulk sorted into the 20 clusters defined by the gating shown in the left flow cytometry panel. The three heatmaps display the percentage of in-frame alleles (green), frameshift alleles (blue), and LD (red) for each cluster. Each cluster showed a distinct indel profile, confirming that changes in GFP/BFP intensity reflect underlying genotype. Unmodified and in-frame alleles predominated in high MFI clusters; frameshift indels enriched in medium/low MFI clusters; LD was most frequent in dim/negative clusters. A similar cluster-specific editing pattern was observed for both gRNAs. The values shown within each heatmap cluster represent the average of two replicates. (F) To pinpoint which frameshift indels drive medium vs. low MFI clusters, we analyzed frameshift indels and the resulting nonsense (NS) codon position in the *HBB* exon. The *x* axis represents the frameshift indel sizes, and the "NS" labels shown above each peak indicate the codon position of the premature nonsense mutation generated by each specific indel size. β-globin codon numbering is used (sickle = codon 6). For R-66S, medium-MFI clusters (GFP^{med}BFP^{high}, GFP^{high}BFP^{med}, and GFP^{med}BFP^{med}) were enriched for NS12, NS15, and NS18, indicating that NS < 19 permits NMD bypass and maintains *HBB*$^{GFP/BFP}$ expression. (G) Low-MFI clusters (GFP^{low}BFP^{high}, GFP^{high}BFP^{low}, and GFP^{low}BFP^{low}) mapped to NS19–21, showing that NS $\geq$ 19 triggers robust NMD, reducing *HBB*$^{GFP/BFP}$ expression. R-02 showed a similar trend: (H) earlier NS codon is associated with medium MFI, whereas (I) later NS is associated with low MFI. For all heatmaps, individual data points and standard deviations are shown in Figure S15.

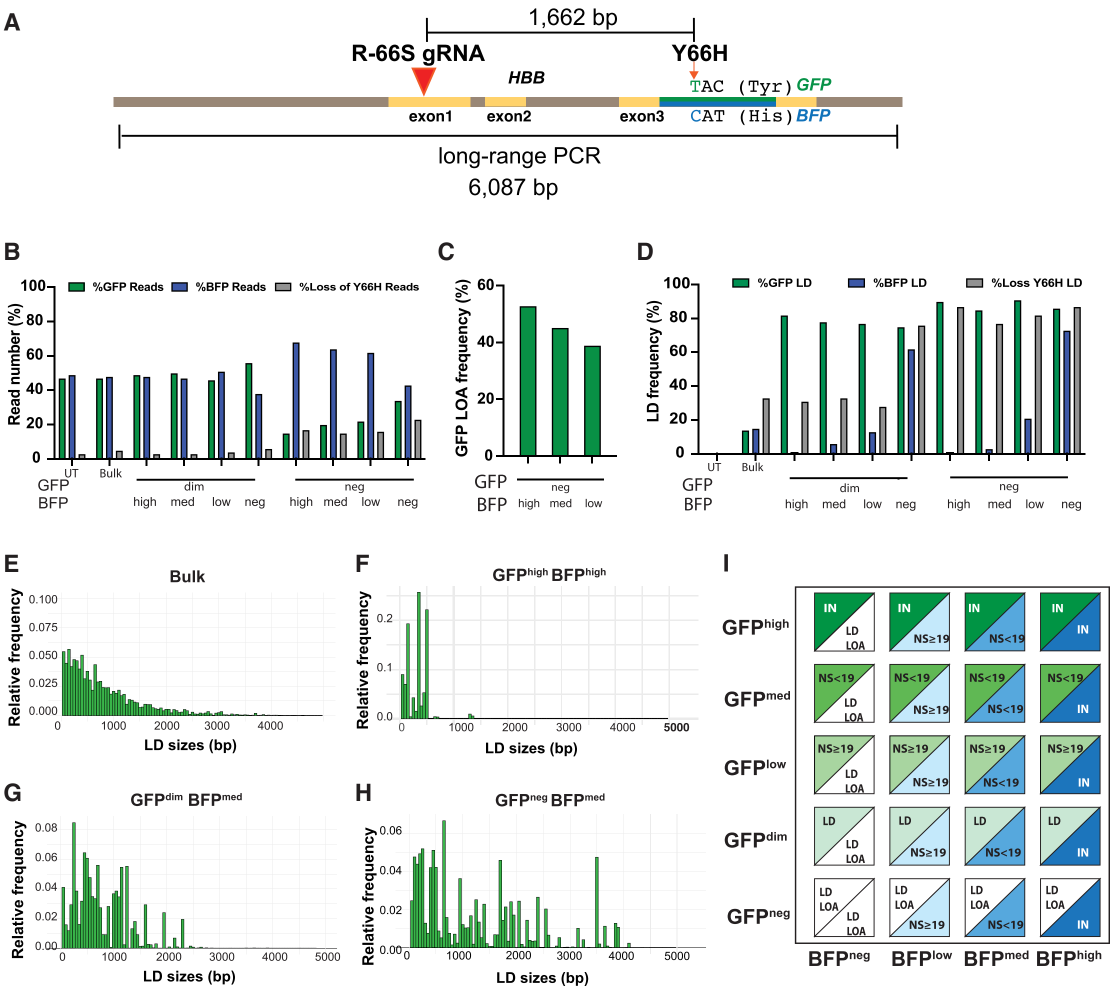

Figure 4. Allele-specific large deletions and loss of allele revealed by long-read sequencing

(A) Schematic of the 6 kb long-range PCR amplicon spanning the R-66S cut-site and the Y66H mutation site, located 1,662 bp downstream. The Y66H mutation converts GFP (TAC, Tyr) to BFP (CAT, His) and was used to distinguish alleles during SMRT long-read sequencing to quantify allele-specific LDs induced by Cas9-mediated DSBs. LDs extending beyond the Y66H site but still amplifiable were defined as "Loss of Y66H reads." Events preventing PCR amplification, consistent with gross chromosomal modifications, were defined as "LOA." (B) Proportions of GFP reads, BFP reads, and Loss of Y66H reads were analyzed. Untreated sample (UT) showed a 1:1 GFP to BFP reads ratio. In GFP^{dim} clusters, most GFP alleles retained the Y666H site, resulting in few Loss of Y66H reads and an approximately 1:1 GFP to BFP read ratio. In contrast, GFP^{neg} clusters with detectable BFP expression showed reduced GFP reads, with an increase in BFP reads and Loss of Y66H reads. A higher proportion of BFP reads than of combined GFP and Loss of Y66H reads in GFP^{neg} clusters indicates frequent GFP LOA undetectable by long-read sequencing. (C) The percentage of GFP LOA in GFP-negative clusters was calculated from the altered GFP:BFP read ratio, confirming that LOA contributes to loss of GFP fluorescence. (D) The proportions of LDs among GFP, BFP, and Loss of Y66H reads demonstrate that loss of GFP or BFP expression correlates with allele-specific LDs. Both GFP^{dim} and GFP^{neg} clusters predominantly contain GFP alleles with LDs, but the higher frequency of Loss of Y66H reads in GFP^{neg} clusters suggests larger LDs in this group. (E–H) Size and distribution of LDs vary across clusters based on MFI, confirming that complete GFP loss corresponds to larger LDs. (E) In the bulk-edited population, LD frequency decreases with size, with most LDs <2,000 bp. (F) In GFP^{high}BFP^{high} cells, LDs are rare and mostly <500 bp, with ~76% of indels in-frame. Comparison of GFP^{neg}BFP^{med} (G) versus GFP^{dim}BFP^{med} (H) shows a significant enrichment of LDs >2,000 bp in the GFP^{neg} cluster. (I) Summary of GFP- and BFP-allele-specific genotype based on NGS and long-read sequencing across 20 clusters in R-66S edited samples. Alleles with no frame changes (IN), including unmodified, HDR, and small in-frame indels, show high MFI. Alleles with NS mutation before codon 19 (NS < 19) show medium MFI, while those with NS $\geq$ 19 show low MFI. LDs result in dim MFI, and LOA due to extensive deletions or chromosomal alterations leads to complete loss of expression.

ratio. GFP^{dim} clusters with detectable BFP fluorescence showed few Loss-of-Y66H reads and retained the ~1:1 ratio, indicating mainly sub-Y66H deletions on GFP (Figure 4B). By contrast, GFP^{neg} clusters with detectible BFP showed depleted GFP reads together with increased BFP and Loss-of-Y66H reads, consistent with frequent GFP-allele LOA that prevents PCR amplification (Figure 4B). Because detectable BFP implies an intact BFP allele (no BFP-side LOA), BFP read counts serve as an internal reference; thus, deviations from 1:1 reflect LOA on GFP. For example, in GFP^{neg}BFP^{high}, SMRT-seq reported 15% GFP, 68% BFP, and 17%

Loss-of-Y66H reads (Figure 4B). Assigning all Loss-of-Y66H to the GFP allele, the shifted ratio solves $(50-x)/(100-x) = 0.32$, yielding $x = 52.9\%$ GFP LOA events invisible by SMRT-seq (Figure 4C). This approach quantifies GFP LOA in GFP-negative clusters and explains sequencing gaps observed for negative-MFI populations. Figures 4B and 4C highlight representative clusters, while Figure S16 summarizes read counts and LD frequencies across the 19 clusters analyzed by SMRT-seq.

Allele-resolved SMRT-seq reveals enrichment of larger LDs in GFP^{neg} compared to GFP^{dim} clusters

The frequency of large deletions (LDs >200 bp) across GFP reads, BFP reads, and Loss-of-Y66H reads demonstrated a tight relationship between fluorescence loss and allele-specific LDs. Both GFP^{dim} and GFP^{neg} clusters were dominated by GFP alleles carrying LDs, but GFP^{neg} clusters showed a higher %GFP LD and a significantly higher %Loss Y66H LD, indicating frequent deletions extending beyond the Y66H site (Figure 4D). LD-size-distribution analysis (Figures 4E–4H; Figure S17) showed that LD frequency declined with increasing size, with most LDs in bulk-edited cells <2 kb (Figure 4E). GFP^{high}BFP^{high} cells contained very few LDs, mostly <500 bp, ~76% of which were in-frame (Figure 4F). In contrast, comparison of GFP^{dim}BFP^{med} (Figure 4G) and GFP^{neg}BFP^{med} (Figure 4H) clusters demonstrated a marked enrichment of LDs >2 kb in GFP^{neg} cells. Overall, GFP^{neg} clusters have higher GFP LOA (Figure 4C), more Loss-of-Y66H LDs (Figure 4D), and larger GFP LD than GFP^{dim} clusters (Figures 4G and 4H). Integrating NGS, ddPCR, and SMRT-seq across sorted clusters establishes a quantitative relationship between MFI and genotype: in-frame alleles maintain high expression; NS < 19 frameshift indels yield medium expression; NS $\geq$ 19 produce low expression; LDs generate dim signals; and LOA from extensive deletions or chromosomal rearrangements leads to complete loss. The allele-specific genotype composition for all 20 clusters is summarized in Figure 4I.

Reporter fluorescence mirrors *HBB* transcript abundance and β-globin production

To confirm that the dual-fluorescent reporter faithfully reflects endogenous *HBB* output, we tested R-66S-RNP-edited SHD$^{GFP/BFP}$ cells on three points: (1) GFP- and BFP-tagged alleles are transcribed evenly in unedited cells, assessed by cDNA NGS (GFP:BFP mRNA ratios); (2) total *HBB* mRNA matches combined GFP+BFP mRNA, and each allele's mRNA level correlates with its corresponding GFP or BFP protein MFI by flow cytometry, assessed by qPCR (total *HBB* and GFP+BFP mRNA); and (3) total reporter fluorescence tracks β-globin production, assessed by western blotting for β-globin and GFP+BFP. Because DNA, RNA, and protein assays require large cell numbers, we first sorted 12 fluorescence-defined clusters during expansion, expanded each population, and then initiated differentiation. These 12 clusters remained separable after differentiation, with some spreading among low/negative MFI groups (Figure S18). Cluster IDs (1–12) shown in Figure S18 are used consistently in Figures S19 and S20 to denote the same fluorescence-defined populations. Genotyping confirmed the expected relationship between MFI and edit class, albeit with slightly lower resolution than the 20-cluster map generated during differentiation (Figure S19A). In UT and bulk RNP-edited cells, GFP:BFP mRNA remained ~1:1, showing similar transcription and stability from both alleles. The relative proportion of GFP and BFP mRNA mirrors their flow cytometry protein MFI (Figure S19B). (GFP+BFP) mRNA matched total *HBB* levels in all sorted clusters, showing that reporter signals track *HBB* transcription (Figure S19C). Western blots showed efficient P2A cleavage, and the summed GFP+BFP signal closely paralleled β-globin abundance across clusters, indicating stoichiometric co-translation from the shared *HBB*-P2A-GFP/BFP transcript (Figure S20).

Diverse *HBB* genotypes differentially modulate HbF induction, while LOA events fail to induce HbF

CRISPR/Cas9 editing at *HBB* generates a broad spectrum of non-HDR outcomes, yet their phenotypic consequences remain poorly defined. This question has gained clinical urgency with the report of expanded frameshift clones accompanied by robust HbF induction via an unclear mechanism.[17] These observations raised the possibility that certain non-HDR alleles may confer a selective or functional advantage, but the specific *HBB* mutations that drive this effect and how remain unknown. To dissect how individual genotypes influence HbF induction, we used SHD$^{GFP/BFP}$, which is well suited for this purpose because it recapitulates adult β-globin dominance with silenced γ-globin,[34,44] enabling sensitive quantification of HbF, with >30%–40% F-cells indicative of a robust response.[32,45,46] Looking ahead, once high-HbF-associated genotypes are defined, the corresponding GFP/BFP clusters could be isolated as live cells, eliminating the fixation required for intracellular HbF staining and preserving DNA and chromatin for downstream mechanistic studies, including analysis of chromatin architecture,[44] transcription factor occupancy, and transcription activity at the globin locus.[47]

First, we quantified HbF induction in bulk edited SHD$^{GFP/BFP}$ cells. At day 8 of differentiation, R-66S RNP increased HbF from 17.5% (0.3%) in mock to 53.7% (0.4%) (p = 0.0001) and R-02 to 40.5% (3.9%) (p = 0.074). Co-delivery of ssODN reduced but did not abolish HbF induction (35.0% [3.5%] for R-66S; 33.8% [1.7%] for R-02), with levels remaining above mock (Figure 5A). HPLC at day 11 corroborated these trends: RNP-only editing produced near-complete loss of HbS with robust HbF induction, whereas ssODN delivery restored HbA and yielded more moderate HbF increases (Figure 5B).

Using matched genotype (NGS and ddPCR; Figures 3D and 3E) and phenotype (%HbF) (Figures 5C and 5D; Figure S21) datasets across all 20 fluorescence-defined clusters, we observed distinct HbF responses associated with specific genotypes. Trends are consistent across gRNAs in both RNP (Figure 5C) and RNP + ssODN (Figure 5D) conditions. ssODN-treated samples show clearer separation, as reduced cluster crowding improved distinction and resolution of cell populations by flow cytometry. In-frame genotypes (GFP^{high}BFP^{high}) showed HbF levels comparable to mock

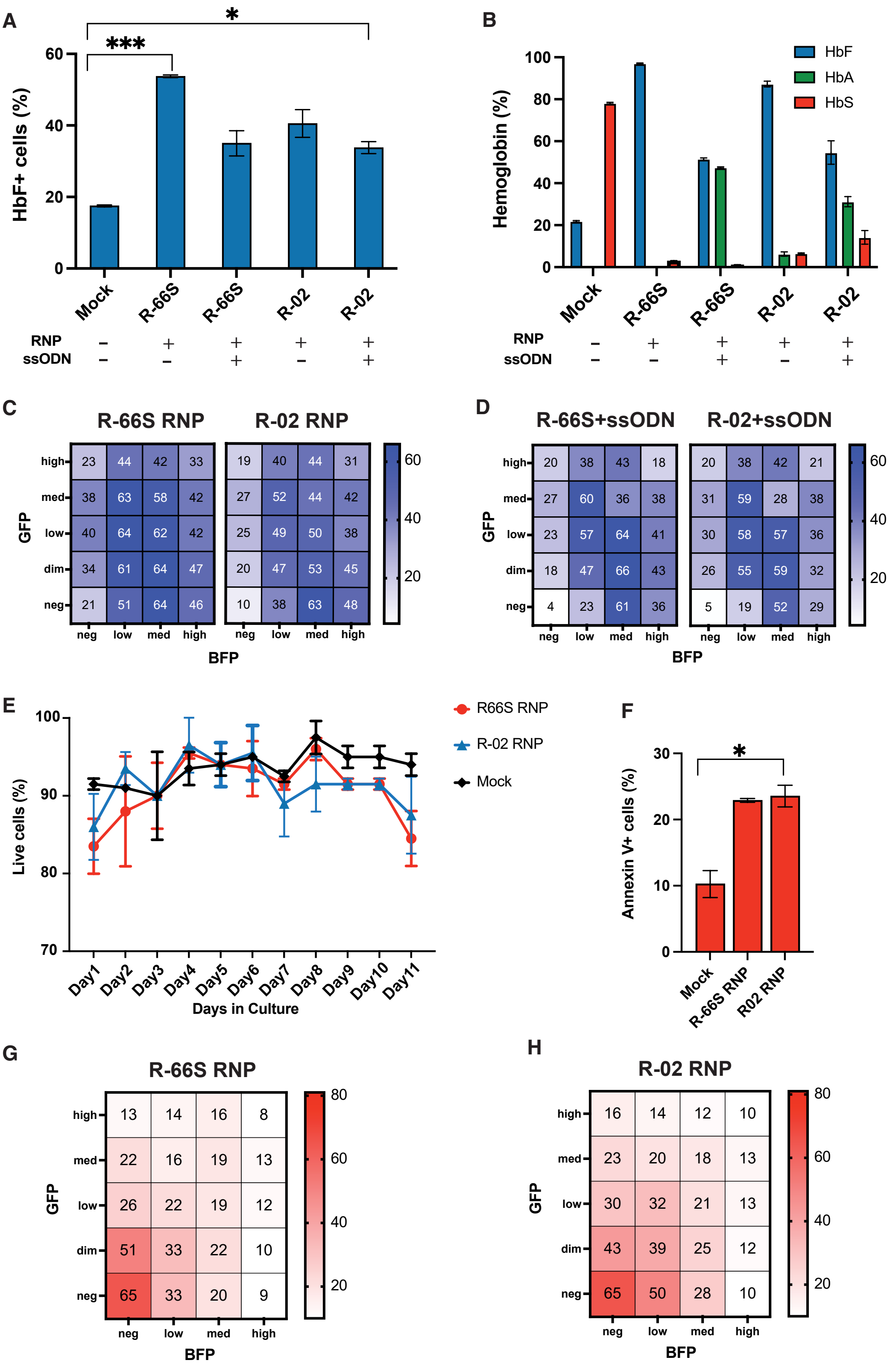

(legend on next page)

(Figures 5C and 5D), confirming that preserved β-globin does not activate γ-globin. In contrast, biallelic frameshift genotypes induced strong HbF, with NS ≥ 19 (GFP^{low}BFP^{low}) producing higher levels (57% HbF^{+} for R-66S; 58% for R-02) and NS < 19 (GFP^{med}BFP^{med}) producing a more modest response (36% for R-66S; 28% for R-02), consistent with more complete β-globin loss driving stronger HbF induction (Figure 5D). These findings align with prior observations that R-02-induced frameshift indels upregulate γ-globin in erythroid colonies[9] and in a treated patient.[17]

LD-containing alleles paired with frameshifts also induced high HbF (GFP^{dim}BFP^{low}: 47% for R-66S, 55% for R-02; GFP^{dim}BFP^{med}: 66% for R-66S, 59% for R-02) (Figure 5D). Because these LDs delete variable segments around the Cas9 cut site, they can remove *HBB* coding sequence and/or promoter. Prior work shows *HBB* promoter deletion can relieve promoter competition and reactivate γ-globin more strongly than loss of coding sequence alone,[48] suggesting a potential mechanism for the robust HbF induction we observe. Future studies sorting HbF^{-}, HbF^{low}, and HbF^{high} subpopulations within LD-containing clusters and assessing *HBB* promoter copy number could determine whether promoter-deleting LDs specifically drive this effect.

In striking contrast, LOA genotypes did not induce HbF, despite being biallelic disruptive events (Figures 5C and 5D). With the exception of a GFP^{neg}BFP^{med}, LOA-enriched clusters showed substantially lower HbF than clusters with the same second allele but enriched for LD or frameshift. The GFP^{neg}BFP^{neg} cluster—most enriched for LOA—showed the lowest HbF (4% for R-66S; 5% for R-02), even below mock (Figure 5D). These findings indicate that LOA lesions are qualitatively distinct from frameshift or LD with respect to globin regulation. One plausible explanation is that LOA events extend beyond *HBB* and disrupt elements required for γ-globin expression—such as the *HBG1/2* gene located ∼21 kb upstream of the cut site or the local chromatin architecture (Figure S22)—thereby abolishing HbF induction even when β-globin is absent. Alternatively, severe β-globin loss may drive ineffective erythropoiesis, preventing cells from reaching maturation stages where HbF is expressed, even if *HBG* remains intact. Together, these observations highlight LOA as a distinct and potentially more deleterious outcome than other disruptive alleles. *HBG* copy number and maturation analyses will further define how LOA suppresses HbF.

Multiple clusters showed non-additive genotype-phenotype behavior. For example, NS < 19 alleles paired with NS ≥ 19 (GFP^{med}BFP^{low} and GFP^{low}BFP^{med}) produced HbF levels comparable to biallelic NS ≥ 19 (GFP^{low}BFP^{low}). Similarly, NS < 19 paired with LD or LOA (GFP^{dim}BFP^{med} or GFP^{neg}BFP^{med}) drove higher HbF than the corresponding NS ≥ 19 paired with LD or LOA (GFP^{dim}BFP^{low} or GFP^{neg}BFP^{low}), even though NS < 19 is a weaker HbF driver than NS ≥ 19 in the biallelic setting. We speculate that these patterns reflect interactions between qualitatively different *HBB* lesions. NS < 19 alleles may bypass NMD and produce truncated, unstable β-globin that adds proteotoxic stress to the profound β-globin deficiency caused by NS ≥ 19, LD, or LOA alleles. The combined burden could push erythroid cells past a threshold for γ-globin activation, consistent with known effects of unstable globin peptides,[49] threshold-like HbF induction seen in deletional HPFH and δβ-thalassemia,[50] and stress-mediated HbF upregulation.[51] A second, non-exclusive explanation is allelic non-independence; because Cas9 cleaves both alleles within a shared repair window, genotype combinations may reflect cell-intrinsic repair biases rather than random pairing. Under this model, NS < 19 alleles

Figure 5. SHD$^{GFP/BFP}$ identifies genotypes that drive robust HbF induction and those that impair erythropoiesis after *HBB* editing

SHD$^{GFP/BFP}$ were edited with R-66S or R-02 RNP ± ssODN (allele and genotype frequencies quantified in Figure 3), differentiated, and assayed for HbF induction and viability. (A) Day-8 flow cytometry quantifies HbF^{+} cells. RNP treatment with either gRNA increases HbF relative to mock; RNP + ssODN also increases HbF, but to a lesser extent than RNP alone. HbF increase for R-66S RNP and R-02+ssODN relative to mock is statistically significant with $p = 0.0001$ and $p = 0.042$, respectively, using a 2-sided Welch *t* test (B). HPLC on day 11 shows near complete loss of HbS after RNP cutting at *HBB* with either gRNA, accompanied by significant HbF induction compared to mock. With ssODN, HDR restores HbA production and yields a more moderate HbF increase than RNP alone, mirroring the flow cytometry trend. (C and D) Genotypes underlying HbF induction. Heatmaps showing %HbF^{+} cells across 20 fluorescent-defined clusters from the same bulk-edited samples analyzed in (A): (C) R-66S RNP (left) and R-02 RNP (right) and (D) R-66S + ssODN (left) and R-02 + ssODN (right). Trends are consistent across gRNAs in both RNP and RNP + ssODN conditions. ssODN-treated samples show clearer separation, as reduced cluster crowding improved distinction and resolution of cell populations by flow cytometry. GFP^{high}BFP^{high} (biallelic in-frame) is comparable to mock, indicating in-frame alleles do not induce HbF. Overall, biallelic *HBB*-disruptive genotypes produce the strongest HbF induction, whereas genotypes with one in-frame allele show less. The GFP^{med}BFP^{med} cluster (biallelic NS < 19) shows attenuated induction relative to biallelic genotypes containing NS ≥ 19 allele, suggesting NS ≥ 19 contributes more strongly to HbF than NS < 19. However, when paired with LD on the other allele, GFP^{dim}BFP^{med} (LD/NS < 19) shows the highest %HbF^{+}, exceeding GFP^{dim}BFP^{low} (LD/NS ≥ 19). LOA clusters showed low HbF despite biallelic *HBB* disruption, consistent with HbF increases arising from the frameshifted allele rather than the LOA allele. GFP^{neg}/BFP^{neg} has the lowest HbF^{+} fraction, even below mock, suggesting that on-target *HBB* cleavage may cause collateral HBG disruption via large genomic alterations. (E) Trypan blue counts over the differentiation time course (to day 11) show a clear viability drop in RNP-treated groups by day 11, not observed in mock controls. The decline emerges post-maturation, consistent with impaired hemoglobin production and erythropoiesis. (F) Day 11 Annexin V^{+} (apoptotic) percentages were higher in RNP-treated cells than in mock controls, with a trend toward increased apoptosis for R-66S ($p = 0.068$) and a significant increase for R-02 ($p = 0.021$), based on a two-sided Welch *t* test. (G and H) Heatmaps showing %Annexin V^{+} cells across 20 fluorescence-defined clusters from the same bulk-edited samples as in (F). (G) R-66S-RNP- and (H) R-02-RNP-edited samples. Compared with mock and in-frame genotypes, which show similar low levels of apoptosis, biallelic *HBB* disruptive genotypes show increased apoptosis. Annexin V^{+} frequency inversely correlates with GFP/BFP MFI across genotypes: it is lowest in high MFI (in-frame) clusters and increases through med MFI (NS < 19), low MFI (NS ≥ 19), and dim MFI (LD). The GFP^{neg}/BFP^{neg} cluster shows the highest Annexin V^{+}, consistent with predominant LD/LOA and suggesting that loss of HbS without compensatory HbF induction creates a severe β-thalassemia-like state. For all heatmaps, individual data points and standard deviations are shown in Figure S21.

could preferentially co-occur with a subset of LD/LOA that yield unexpectedly high HbF in NS < 19/LOA clusters, even though NS < 19 or LOA alone is a weak HbF driver in other settings. The $SHD^{GFP/BFP}$ enables direct testing of this hypothesis by allowing cells to be sorted from NS < 19/LOA and NS ≥ 19/LOA clusters and comparing their LOA breakpoint spectra to determine whether specific lesions are enriched in HbF^{high} cells.

Collectively, these findings define a genotype-phenotype hierarchy: (1) preserved β-globin (in-frame) does not activate γ-globin; (2) biallelic frameshift/NMD and LD genotypes induce progressively stronger HbF in proportion to functional β-globin depletion; (3) LOA events uniquely fail to induce HbF due to likely disruption of γ-globin expression or maturation failure; and (4) specific allele combinations exhibit emergent, non-additive HbF behavior reflecting interactions between β-globin dosage, proteostasis burden, and locus structure. This allele-resolved framework provides mechanistic insight into how distinct non-HDR repair outcomes shape fetal globin compensation and identifies LOA as a quantitatively and qualitatively different class of lesions with important safety implications.

Genotype-dependent apoptosis highlights risks of LOA

To assess how distinct *HBB* genotypes influence erythropoiesis and cellular fitness, we examined viability and apoptosis across edited populations. Trypan blue staining showed similar percentages of live cells during early differentiation, but viability in both R-66S- and R-02-RNP-treated cultures began to decline relative to mock by day 9, consistent with globin-chain-imbalance-driven toxicity (Figure 5E). This loss of viability was accompanied by increased Annexin V^{+} cells (Figure 5F). On day 11 of differentiation, mapping Annexin V positivity onto the 20 fluorescence-defined clusters (Figures 5G and 5H; Figure S21) showed that cells with intact or in-frame alleles ($GFP^{high}BFP^{high}$) exhibited low apoptosis, similar to mock, whereas $GFP^{neg}BFP^{neg}$ cells, enriched for biallelic LD/LOA, displayed the highest Annexin V positivity, followed by $GFP^{dim}BFP^{neg}$ and $GFP^{neg}BFP^{low}$, showing clusters unable to induce HbF despite complete *HBB* disruption show the strongest apoptosis. Early erythroid precursor apoptosis due to α/β chain imbalance is a hallmark of β-thalassemia,[52] and similar mechanisms likely underlie the severe apoptosis observed in LOA-enriched clusters. Clinically, if biallelic LOA/LD-edited cells were to engraft, they could contribute little to mature red blood cell (RBC) production while provoking compensatory responses reminiscent of β-thalassemia syndromes, including marrow expansion, chronic anemia, excessive iron absorption, and skeletal abnormalities.[53]

In $SHD^{GFP/BFP}$, reduced GFP/BFP MFI is expected to reflect disruptive *HBB* genotypes, and we observe an inverse relationship between MFI and apoptosis, with lower MFI clusters highly enriched for Annexin V^{+} cells (Figures 5G and 5H). This pattern indicates that β-globin deficiency is a major contributor to apoptosis, even across clusters with differing levels of HbF induction (Figures 5C and 5D). Because mock-treated cells maintain high viability (Figure 5F), the excess apoptosis and loss of viability are attributable to gene editing. However, apoptotic cells can also downregulate or lose reporter signal, so some unhealthy cells without LOA may be mis-assigned to $GFP^{neg}BFP^{neg}$ as their fluorescence declines. As a result, our current measurements may overestimate apoptosis within the $GFP^{neg}BFP^{neg}$ population. Future work using this cell model will leverage GFP/BFP-based presorting of live Annexin V^{-} clusters, followed by longitudinal Annexin V profiling, to determine whether specific genotype-defined populations have an intrinsically higher apoptotic propensity. In parallel, sorting Annexin V^{-} versus Annexin V^{+} cells within the $GFP^{neg}BFP^{neg}$ cluster may help pinpoint which LOA subtypes most strongly impair maturation and drive cell death, illustrating how this $SHD^{GFP/BFP}$ can be used to mechanistically dissect high-risk editing outcomes beyond the scope of the present study.

$SHD^{GFP/BFP}$ recapitulates HSPC responses to DNA-repair modulators that enhance HDR but increase LOA

A growing class of HDR-boosting strategies, including pharmacologic DNA-repair modulators, is rapidly entering practice. While these agents can improve HDR, they may also shift repair outcomes in ways that impact safety (e.g., increased LDs, translocations, loss of heterozygosity (LOH)).[28,54–56] We therefore asked whether $SHD^{GFP/BFP}$ can predict how DNA-repair modulators reshape editing outcomes, benchmarking against primary SCD HSPCs. $SHD^{GFP/BFP}$ and SCD HSPCs were edited with R-66S or R-02 RNP ± ssODN in the presence of the DNA-dependent protein kinase (DNA-PKcs) NHEJ inhibitor M3814, the HDR enhancer protein (HEP), or the DNA polymerase theta (Polθ) MMEJ inhibitor ART558. Prior studies have shown that pharmacologic NHEJ inhibition can increase HDR as measured by NGS,[57,58] yet simultaneously inflate kilobase-scale deletions, chromosome-arm loss, and translocations, many of which escape detection due to allelic drop-out,[54,55] underscoring the need for assays beyond standard NGS. HEP is a recombinant engineered ubiquitin variant that selectively inhibits 53BP1, relieving its block on end resection and promoting HDR.[59] HEP has been reported to boost HDR with minimal acute cytotoxicity and reduced off-target effects,[60] but its impact on LD and LOA remains unknown. In parallel, DNA Pol θ-mediated end joining (TMEJ) has been implicated in the repair of Cas9-induced LDs, motivating Polθ inhibition (e.g., ART558/M4344) as a strategy to reduce these events,[61] whereas a more recent preprint reports high LOH levels after Polθ inhibition, raising safety concerns because LOH is difficult to detect or deplete.[55] Together, these data motivated a systematic evaluation of all three modulators using our $SHD^{GFP/BFP}$, which can quantify LD and LOA at single-allele resolution and in SCD HSPCs.

DNA-PK inhibition by M3814

Following R-66S RNP + ssODN electroporation, cells were exposed to 0, 0.5, 1, and 2 μM M3814 for 24 h, then cultured without drug. M3814 caused transient toxicity relative to untreated controls, but cell viability recovered by day 4 post-electroporation (Figure S23). M3814 induced a dose-dependent increase in HDR with a corresponding reduction in NHEJ (Figure 6A). Flow cytometry (Figure S24) showed that HDR

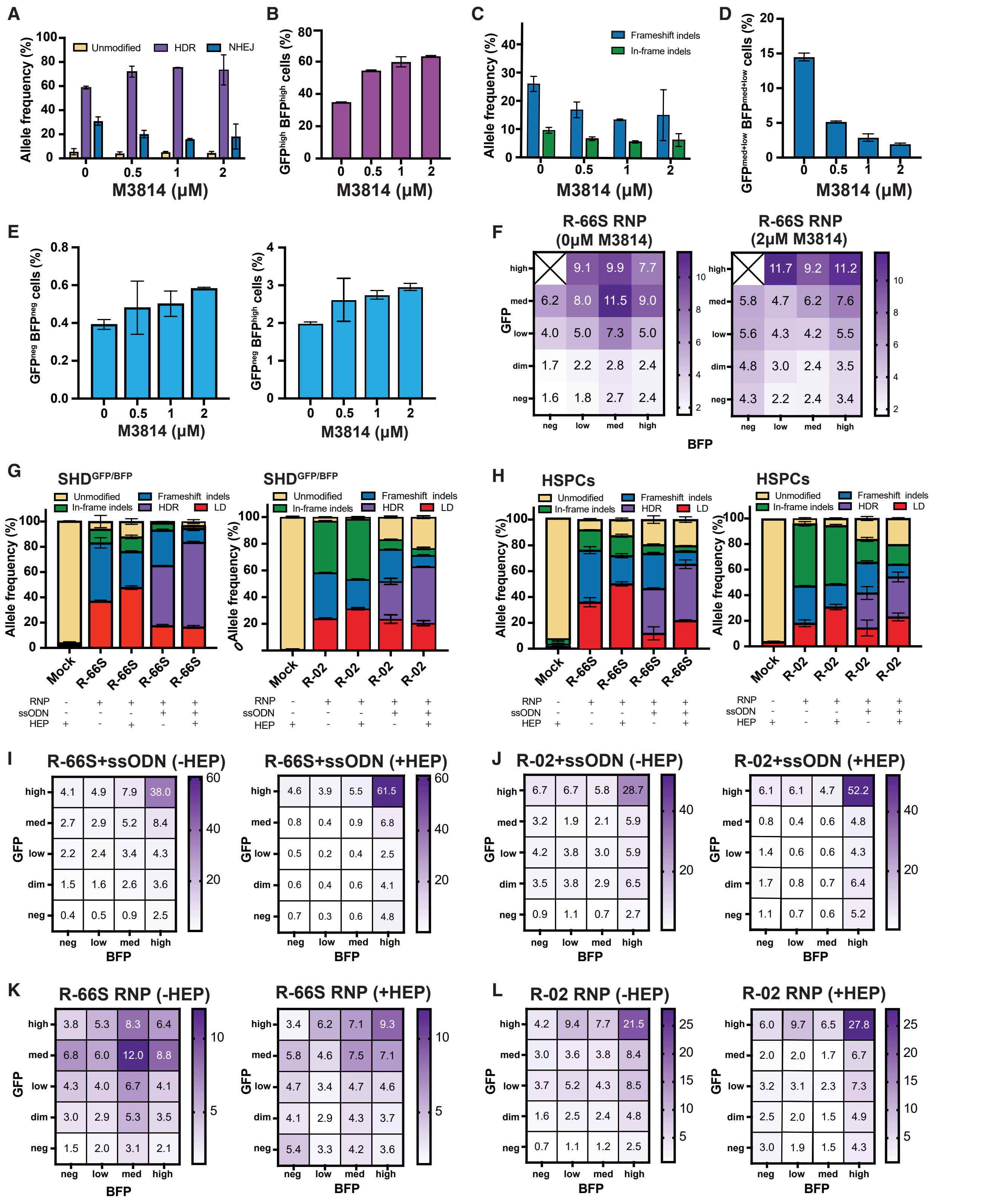

(legend on next page)

enhancement coincided with expansion of the GFP^{high}BFP^{high} population (Figure 6B), and NGS confirmed selective depletion of frameshift (NHEJ-repaired) indels while maintaining in-frame edits (Figure 6C). Consistent with these findings, intermediate-MFI clusters corresponding to biallelic frameshifts were reduced (Figure 6D), whereas LOA-associated populations (GFP^{neg}BFP^{neg} and GFP^{neg} BFP^{high}; Figures 6E and 6F) increased in a dose-dependent manner. These data indicate that although M3814 boosts HDR, it does not suppress it and may even increase LOA formation. Validation in SCD HSPCs showed similar responses for both gRNAs: 0–4 μM M3814 caused transient cytotoxicity, but viability recovered to near-mock levels (Figure S25). HDR increased with dose up to 2 μM and then plateaued, while frameshift indels decreased and in-frame edits were preserved (Figure S26). These parallels demonstrate that SHD$^{GFP/BFP}$ recapitulates NHEJ-inhibitor responses in SCD HSPCs, supporting its use as a surrogate platform for screening DNA repair modulators. Because LOA increased with M3814 in the RNP+ssODN condition, we next tested M3814 with RNP only to isolate its effects in the absence of HDR and to determine whether LD and LOA formation are NHEJ-dependent (Figure 6F; Figures S27 and S28). Fold change analysis for R-66S RNP (0 μM vs. 2 μM M3814) showed selective enrichment of clusters associated with LD/LOA (GFP^{neg} BFP^{neg} and GFP^{dim}BFP^{neg} clusters increased 2.7- to 2.8-fold, and GFP^{dim}BFP^{high} and GFP^{neg}BFP^{high} clusters increased 1.4- to 1.5-fold) (Figure S28A). Conversely, intermediate-MFI clusters composed primarily of frameshift indels were depleted.

HDR enhancer protein

To directly compare outcomes, we electroporated 25 μM HEP with R-66S or R-02 RNP ± ssODN in SHD$^{GFP/BFP}$ and SCD HSPCs. With RNP only, HEP increased LD and decreased frameshift indel for both gRNAs. With ssODN, HEP increased HDR and reduced small indels in SHD$^{GFP/BFP}$ for both gRNAs (Figure 6G; Figure S29). In SCD HSPCs, HEP produced comparable but smaller HDR gains and a marked increase in LDs under both RNP-only and ssODN conditions (Figure 6H), suggesting that enhanced end resection in primary cells favors LD formation over HDR. In SHD$^{GFP/BFP}$, HEP had concordant effects across both gRNAs. With ssODN, HEP increased the GFP^{high}BFP^{high} (HDR) population and reduced intermediate-MFI clusters but also expanded GFP^{neg}BFP^{neg} (1.7-fold for R-66S, 1.3-fold for R-02) and GFP^{neg}BFP^{high} (1.9-fold for R-66S, 2.0-fold for R-02) populations (Figures 6I and 6J; Figure S29A), mirroring the pattern observed with M3814+ssODN (Figure S23C). Without donor, HEP produced even stronger effects on LOA-enriched populations, with GFP^{neg}BFP^{neg} increasing 3.6-fold (R-66S) and 4.5-fold (R-02) (Figures 6K and 6L; Figure S30B), exceeding the 2.7-fold rise seen with 2 μM M3814 for R-66S RNP (Figure S28).

Polθ inhibition by ART558

In SHD$^{GFP/BFP}$ cells treated with R-66S RNP and ART558 (0, 2, 5 μM), we observed a dose-dependent decrease in GFP^{dim} clusters enriched for LD but a 1.2- to 1.5-fold increase in GFP^{neg} clusters enriched for LOA (Figure S31). Thus, Polθ/MMEJ inhibition reduces detectable LDs yet paradoxically increases LOA.

Implications for LOA mechanisms and detection

Across all three modulators, LOA consistently increased, raising important questions about the repair pathways that generate LOA. Because LOA alleles are not amplifiable by 6-kb SMRT-seq, their structures (e.g., multi-kb/MB deletions, chromosomal truncations, chromosome loss, or LOH) cannot yet be resolved. Additional assays, such as fluorescence *in situ* hybridization (FISH), long-read whole genome sequencing (WGS), or chromosomal aberrations analysis by single targeted linker-mediated PCR sequencing (CAST-seq),[62] will be required to resolve their architecture. As shown in Figure 4, 6-kb long-read sequencing misses LOA, and bulk ddPCR underestimates modest LOA increases, underscoring the risk of under-calling LOA with conventional methods and demonstrating the value of SHD$^{GFP/BFP}$ for detecting rare, large, and potentially deleterious outcomes.

Figure 6. SHD$^{GFP/BFP}$ model reports both HDR enhancement and LOA risk from DNA repair modulators, mirroring responses in SCD HSPCs
SHD$^{GFP/BFP}$ and SCD HSPCs were edited with R-66S or R-02 gRNAs ± the HDR enhancers M3814 and Alt-R HDR enhancer protein (HEP). Editing outcomes were quantified by flow cytometry and NGS/ddPCR in SHD$^{GFP/BFP}$ and by NGS/ddPCR in SCD HSPCs. In both systems, HDR enhancers increased HDR but also elevated LOA. (A–E) SHD$^{GFP/BFP}$ edited with R-66S RNP + ssODN and treated with M3814 at 0, 0.5, 1, or 2 μM. (A) NGS shows a dose-dependent HDR increase with a concomitant NHEJ decrease. (B) After 4 days of erythroid differentiation, flow cytometry shows a dose-dependent rise in GFP^{high}BFP^{high} cells, matching NGS trends and directly reflecting HDR enhancement. (C) M3814 reduces frameshift indels while maintaining in-frame indels by NGS, consistent with selective NHEJ inhibition. (D) In line with the NGS data, flow cytometry shows a dose-dependent reduction in intermediate MFI clusters (GFP^{med}BFP^{med}, GFP^{low}BFP^{low}, GFP^{med}BFP^{low}, and GFP^{low}BFP^{med}), which represent biallelic frameshift indels. (E) M3814 caused a dose-dependent increase in LOA populations, shown as GFP^{neg}BFP^{neg} cells (left panel) and GFP^{neg}BFP^{high} cells (right panel), although the increase was not statistically significant. (F) Heatmap of cluster percentages in SHD$^{GFP/BFP}$ edited with R-66S RNP and treated with M3814 at 0 μM (left panel) vs. 2 μM (right panel) shows a significant increase in GFP^{neg}BFP^{neg} and GFP^{dim}BFP^{neg}, indicating NHEJ inhibition does not suppress LOA or LD formation. (G–L) Effect of HEP in SHD$^{GFP/BFP}$ and SCD HSPCs edited with R-66S or R-02 RNP ± ssODN. (G) Allele frequencies in SHD$^{GFP/BFP}$ electroporated with R-66S (left panel) and R-02 (right panel) RNP ± ssODN, ±25 μM HEP, quantified by NGS/ddPCR. With RNP alone, HEP increased LD and decreased frameshift indels. With ssODN, HEP increased HDR while decreasing frameshift indels. (H) SCD HSPCs treated and analyzed as in (G) showed similar trends for both R-66S (left panel) and R-02 (right panel). With ssODN, HEP produced smaller HDR gains than in SHD$^{GFP/BFP}$ and increased LD under both RNP-only and ssODN conditions, indicating that enhanced end resection in HSPCs favors LD formation over HDR. (I–L) Heatmap shows the percentages of cells in each of 20 fluorescent-defined clusters in SHD$^{GFP/BFP}$ edited without (left panel) and with 25 μM HEP (right panel), measured by flow cytometry 4 days differentiation post editing: (I) R-66S RNP + ssODN, (J) R-02 RNP + ssODN, (K) R-66S RNP, and (L) R-02 RNP. With ssODN, HEP significantly increases HDR (more GFP^{high}BFP^{high} cells) and reduces frameshift indels (fewer intermediate MFI cells). As with M3814, HEP also increased GFP^{neg}BFP^{neg} and GFP^{neg}BFP^{high} populations, indicating enrichment of large genomic events. With RNP only, promoting end resection enriched LOA populations, as evidenced by increases in GFP^{neg}BFP$^{neg, low, med, high}$ and GFP^{dim}BFP^{neg} clusters. The same trend was observed for both gRNAs. (A–L) Data represent the mean of $n = 2$ independent editing reactions. For all heatmaps, individual data points and standard deviations are shown in Figures S27 and S29.

DISCUSSION

CRISPR/Cas9 editing has opened a path toward curative therapies for SCD, but DSBs at *HBB* can generate a wide spectrum of unintended outcomes, including frameshift indels, LDs, and LOA, whose biological consequences remain incompletely understood. Reflecting this concern, the FDA now emphasizes the need for systematic assessment of on-target genotoxicity in gene-editing products.[63] However, HSPCs are short-lived *ex vivo* and poorly suited for high-resolution genotype-phenotype analysis. To address this challenge, we developed an allele-resolved fluorescent reporter cell model (SHD$^{GFP/BFP}$) that converts *HBB* editing outcomes into ∼20 discrete GFP/BFP clusters in live cells, enabling systematic mapping of editing genotypes, transcriptional consequences, HbF induction, and erythroid cell fitness. Our results reveal a highly structured and predictable genotype-phenotype landscape at *HBB*, inducing genotypes that promote HbF induction and others that produce deleterious β-thalassemia-like phenotypes.

SHD$^{GFP/BFP}$ recapitulates key features of *HBB* DSB repair. In-frame alleles yield high GFP/BFP; NS < 19 alleles partially evade NMD and generate medium fluorescence; NS $\geq$ 19 alleles undergo stronger NMD and low fluorescence; LDs reduce fluorescence to dim levels; and LOA eliminates the signal entirely. Long-read-allele-specific sequencing and ddPCR validated these assignments, revealing that GFP^{neg} clusters are highly enriched for LDs extending hundreds to thousands of base pairs and for extensive LOA not detectable by 6-kb long-read sequencing. Thus, SHD$^{GFP/BFP}$ provides a sensitive live-cell platform for detecting and quantifying large on-target genomic alterations that are routinely under-called in standard NGS, SMRT-seq, and ddPCR.

CRISPR/Cas9 editing at *HBB* yields a wide range of non-HDR outcomes whose functional consequences are difficult to resolve in bulk. The SHD$^{GFP/BFP}$-allele-resolved reporter uncovers subtle, clinically relevant patterns hidden in bulk measurements and reveals a genotype-phenotype hierarchy that explains both therapeutic and deleterious behaviors. Going forward, this model can be leveraged to presort live, genotype-defined populations and longitudinally track outcomes such as apoptosis, HbF induction, and maturation, enabling mechanistic dissection of why some *HBB* lesions promote fetal-globin compensation, whereas others drive erythroid failure. These insights will be essential for the safe design and translation of *HBB*-targeted editing strategies.

Using SHD$^{GFP/BFP}$, we evaluated three classes of DNA-repair modulators: NHEJ inhibition (M3814), 53BP1 inhibition (HEP), and MMEJ inhibition (ART558) and found that all three increased LOA. These findings mirror recent reports that NHEJ or MMEJ inhibition can artificially inflate HDR in short-read assays while simultaneously generating kilobase- to megabase-scale deletions, terminal chromosomal truncations, or LOH events that escape detection.[27,64] SHD$^{GFP/BFP}$ directly visualizes these changes as expansion of GFP^{neg} clusters, providing a readout of genotoxicity that sequencing alone cannot reliably capture. These results underscore the need for caution when modulating DNA-repair pathways in HSC.

SHD$^{GFP/BFP}$ offers several advantages beyond sequencing-based assays. It is live-cell based, allowing prospective sorting of rare genotypes, including LOA-enriched clusters, that would otherwise be lost due to fitness disadvantages in bulk culture. The allele-specific GFP/BFP readout is stable over time, supporting longitudinal analysis of cell fitness, chromatin architecture, and transcriptional regulation. Once HbF-inducing genotypes are defined, their corresponding clusters can be isolated without fixation for HbF staining, preserving native DNA and chromatin for mechanistic studies. These capabilities are not feasible with single-cell sequencing, which captures terminal states but cannot prospectively recover live subclones for functional interrogation.

Several limitations should be acknowledged. GFP and BFP differ in brightness, constraining cluster symmetry; next-generation versions using GFP/mCherry could achieve balanced 5×5 resolution. HUDEP-2 cells do not enucleate, although complementary use of BEL-A cells could extend analysis to terminal maturation.[65] Our 6-kb SMRT-seq cannot resolve the full architecture of LOA alleles; whole-genome long-read sequencing or FISH will be required to define their structural complexity. Finally, while the current configuration cannot always distinguish LD from LOA on a single-cell basis, the genomic position of the reporter provides unique advantages: because *HBB* lies ∼21 kb telomeric to *HBG*, SHD$^{GFP/BFP}$ can function as a live sentinel for genotoxic outcomes arising from *HBG*-targeted editing using SpCas9,[66,67] AsCas12a,[68] and adenine base editor (ABE),[69,70] noting that even base editors can induce LDs, albeit at reduced frequencies.[61] Any >21-kb telomeric LDs or chromosomal truncation originating from the *HBG* locus would eliminate the reporter signal, enabling prospective enrichment and detailed analysis of affected cells.

The SHD$^{GFP/BFP}$ cell model provides a powerful, scalable, allele-resolved platform to quantify the full landscape of on-target editing outcomes, define their functional consequences, and evaluate DNA-repair modulators with a level of resolution not achievable in primary HSPCs. As such, it fills a critical gap for the safe translation of *HBB*-targeted editing strategies. More broadly, the principles we define for DSB repair architecture, the effects of DNA-repair pathway modulation, and genotype-phenotype relationships are generalizable to other therapeutic loci and will inform the wider gene-editing field.

MATERIALS AND METHODS

Cell culture

S-HUDEP2 and CD34+ cells were cultured at 37°C and 5% CO_2. In the expansion phase, S-HUDEP2 cells were cultured in SFEM (Stemcell, Cat No. 09650) supplemented with 50 ng/mL of hSCF (Peprotech), 20 ng/mL of EPO (Peprotech) 1 μg/mL of DOX (Sigma), 1 μM of DEX (Sigma), and 100 units/mL of pen/strep (Gibco). In the differentiation phase, cells were cultured in IMDM supplemented with 100 ng/mL of hSCF, 20 ng/mL of EPO, 331.25 μg/mL of Holo-HTF, 4 μg/mL of Heparin, 10 μg/mL of Insulin, 5% hPlasma, 200 units/mL Pen/strep, and 1 μg/mL of DOX. Fresh medium was added every 1–2 days, and cells were cultured at a density of under

10^6 live cells/mL for up to 14 days and analyzed throughout differentiation.[20] DOX was removed on day 7 of differentiation, and cells were cultured for additional days to continue differentiation, as described for each experiment.

Peripheral blood CD34+ cells were obtained from patients with SCD undergoing therapeutic red cell exchange at the Texas Children's Hospital Cancer & Hematology Centers (Houston, TX), under the approved IRB protocol H-33997. CD34+ cells were extracted from the mononuclear fraction by immunomagnetic separation using CD34 Microbeads Kit (Miltenyi Biotech, CD34 MicroBead Kit UltraPure, human) according to the manufacturer's instructions. CD34+ purity was assessed at 48 and 72 h after extraction by flow cytometry, and cells with purity above 90% were used for experiments. These patients' derived CD34+ HSPCs were cultured and differentiated to erythroblasts using a two-phase primary erythroid culture system. In the expansion phase, cells were cultured in SFEMII (Stemcell Cat. No. 09655) supplemented with 300 ng/mL hSCF (Peprotech), 100 ng/mL TPO (Peprotech), 300 ng/mL Flt3 ligand (Peprotech), and 60 ng/mL IL3 (Peprotech). Cells were then differentiated using two phase erythroid differentiation culture. In phase 1, HSPCs were differentiated in IMDM supplemented with 10 ng/mL SCF, 20 ng/mL EPO, 1 ng/mL IL-3 (PeproTech), 200 μg/mL holo-transferrin human, 10 μg/mL insulin human, 3% human serum (Sigma-Aldrich), 6 μg/mL heparin (Stem Cell Technologies), 2% human plasma (Innovative Research), and 1% penicillin-streptomycin-glutamine (Gibco) for 7 days. Cells were then moved to phase 2 for an additional 7 days, where interleukin-3 (IL-3) was removed. Cells were incubated at 37°C and 5% CO_2. CD34+ cells were cultured for 3 days in the expansion phase before electroporation. Seventy-two h after electroporation, cells were transferred to differentiation media. Fresh medium was added every 1–2 days, and cells were cultured at a density of under 10^6 live cells/mL for 5 days before analysis. Cell count and viability were measured using a 0.4% Trypan Blue solution (Bio-Rad) and a T20 Automated Cell Counter (Bio-Rad).

RNP and ssODN delivery using electroporation

According to the manufacturer's instructions, $1–2 \times 10^5$ S-HUDEP2 and CD34+ cells (program CA-137, solution P3) were electroporated on a Lonza Nucleofector 4D. In S-HUDEP2 and CD34+ cells, 5 μg of HiFi *Sp*Cas9 protein (Integrated DNA Technologies) complexed with 2.5 μg of chemically synthesized gRNAs (Integrated DNA Technologies) as RNP with or without 100 pmol of ssODN were electroporated. For mock-treated S-HUDEP2 and CD34+ cells, the same number of cells was electroporated without RNP or ssODN.

S-HUDEP2 and CD34+ cell drug treatment

HEP: S-HUDEP2 and patient-derived CD34+ were electroporated with RNP + ssODN corrective donor with either 0 μM or 25 μM of HEP (Integrated DNA Technologies Cat # 10029790), based on the manufacturer's recommendations. After electroporation, cells were maintained in expansion culture for 72 h until editing was complete, before transitioning to erythroid differentiation media.

M3814 and ART 558: both M3814 (Selleckchem #S8586) and ART 558 (TargetMol #T9275) were resuspended in DMSO according to the manufacturers' recommendations and solubility guidelines. S-HUDEP2 and patient-derived CD34+ cells were then electroporated with RNP or RNP+ssODN donor, then plated into media containing either M3814 or ART558 at the indicated concentrations. Vehicle control cultures received an equivalent final concentration of DMSO to match the drug-treated conditions. Drug-containing media were removed after 24 h, and cells were maintained in expansion culture for an additional 48 h to allow editing to complete before DNA harvest for downstream analyses.

Flow cytometry analysis

SONY MA900 and BD FACSMelody instruments were used for flow cytometry analysis and cell sorting. For all analyses performed on the SONY MA900, compensation was set using unstained controls, single-color controls, and fluorescence-minus-one (FMO) controls, when applicable. This approach optimized separation of GFP and BFP signals in SHDGFP/BFP cells and enabled sorting of 20 distinct fluorescence-defined clusters. Twenty-way SHDGFP/BFP sorting based on GFP and BFP was performed on the SONY MA900 on days 4 and 5 of erythroid differentiation. Following sorting, cells were maintained in differentiation culture for an additional 24–48 h to allow expansion and ensure adequate DNA yield for downstream analyses. For flow cytometry analysis of erythroid markers in CD34+ cells, the following antibodies were used for analysis: FITC Mouse Anti-Human CD36 (BD 555454), APC-H7 Mouse Anti-Human CD71 (BD Biosciences 563671), and APC Mouse Anti-Human CD235a (BD Biosciences 561775). Erythroid differentiation was assessed between days 10 and 15, with exact time points varying by donor as described in the main text. This window was chosen based on prior time course staining, which showed that differentiation differences based on cell-surface markers were not apparent before day 10 and that cellular health declined substantially after day 15, limiting reliable analysis. The following antibodies were used to assess cellular health, fetal hemoglobin, and erythroid surface markers in S-HUDEP2: APC Mouse Anti-Human Cd36 (BD Biosciences 550956), BV786 Mouse Anti-Human CD235a (BD Biosciences 740984), APC-Fetal Hemoglobin Monoclonal antibody (HBF-1) (Thermo Fisher Scientific MHFH05), and Alexa Fluor 647 Annexin V Apoptosis Detection (Thermo Fisher Scientific A23204). Fetal hemoglobin analysis in S-HUDEP2 was performed on day 8 of erythroid differentiation, based on prior time course data showing that differences in HbF induction across treatments are most pronounced at this time point, while GFP/BFP-defined clusters remain well separated and cell viability is high. Annexin V analysis was conducted on day 10 of erythroid differentiation, as this was the earliest time point at which treatment-dependent differences in Annexin V staining became evident.

Library preparation for targeted amplicon next-generation sequencing

The first PCR (PCR1) amplified a ~300 bp region surrounding the Cas9 cut site. A second PCR (PCR2) was performed to append

dual-index barcodes and Illumina P5/P7 adapters. Equimolar amounts of PCR2 products from each sample were pooled and sequenced on the Illumina NextSeq 2000 using a NextSeq1000/2000 P1 Reagents (600cycles). Demultiplexed FASTQ files were analyzed using CRISPResso2.[71]

LongAmp-seq

LongAmp-seq was performed as previously described.[15] In short, 100 ng of L-R PCR products were used for LongAmp-seq library preparation, which consists of on-bead tagmentation, posttagmentation clean up, 5-cycle PCR to add index adaptors, double-sided bead purification, library pooling, and quantification according to the Nextera DNA Flex Library Prep Reference Guide (Nextera DNA Flex Library Prep Kit [Illumina, 20018704] and Nextera DNA CD Indexes [Illumina, 20018707]). Equimolar amounts of each sample were pooled and sequenced on the Illumina NextSeq 2000 using a NextSeq1000/2000 P1 Reagents (600 cycles) and analyzed using LV_caller[15] and CRISPResso2.[71]

SMRT-seq

The first PCR reaction (PCR1) was used to target a 6-kb region around the Cas9 cut site and tag the template molecule with an adapter using a tailed primer pair. The PCR1 reaction contained 100 ng of gDNA, 200 nM of each tailed primer in 50 μL of reaction (LongAmp Hot Start Taq 2× Master Mix, NEB). The PCR1 program consisted of initial denaturation (2 min at 94°C) and 25 cycles of denaturation (30 s at 94°C), annealing (30 s at 60°C), and extension (6 min at 65°C). After completion of PCR1, the PCR1 product was purified using SPRIselect (Beckman Coulter, B23317) and eluted in 30 μL of water. In second PCR reaction (PCR2), barcodes are incorporated by using universal sequences tailed with 16-bp PacBio barcode sequences (Sequal_RSII_96_barcodes_v1). The PCR2 program consisted of initial denaturation (2 min at 94°C), 5 to 10 cycles of denaturation (15 s at 94°C), annealing (30 s at 60°C), and extension (6 min at 65°C) followed by the final extension (5 min at 65°C). The minimum cycle number (10 cycles) was used to obtain sufficient PCR product (>100 ng) for library preparation. The PCR2 product was purified using SPRIselect and eluted in 30 μL of water. One hundred nanograms of barcoded amplicon from PCR2 was pooled and used for PacBio library preparation, which consists of DNA damage repair, end repair/A-tail, SMRTbell adaptor ligation (SMRTbell Express Template Prep Kit 2.0), nuclease treatment (SMRTbell Enzyme Clean Up Kit), and AMPure bead purification following the standard protocol. The SMRTbell library was sequenced on a PacBio Sequel II 8M flow cell in CCS mode following the standard protocol with 1 h of preextension and 30 h of collection time (PacBio). The PacBio subreads were converted to HiFi reads, and Q20 CCS reads were used for analysis. GFP and BFP allele specific editing rate was quantified by a custom python script (available upon request).

Droplet digital PCR

Probe-based ddPCR assays quantifying allelic drop-off at *HBB* (ROX) relative to the diploid reference gene RPP30 (VIC) were used. Reactions contained 15 ng genomic DNA, 1× ddPCR Supermix for Probes (Bio-Rad), 900 nM target primers, 250 nM target probes (Eurofins Genomics), and 10 U HindIII-HF restriction enzyme in a 20 μL reaction. Thermal cycling was performed using the standard ddPCR protocol recommended by the manufacturer.

qRT-PCR

RNA extraction was done using Qiagen RNeasy Mini Kit, followed by cDNA synthesis using Bio-Rad iScript cDNA Synthesis Kit. GFP- and BFP-specific primers were designed and used to quantify relative levels of GFP and BFP mRNA according to the manufacturer's protocol (Bio-Rad, iTaq Universal SYBR Green Supermix).

Western blot

Protein analysis of individual globin chains (alpha sub-unit, beta sub-unit, and gamma sub-unit), GFP/BFP, and P2a peptide was done using specific antibodies. The 7-day differentiated cells were lysed in cold RIPA buffer supplemented with 1× protease and phosphatase inhibitor cocktail. Samples were vortexed and incubated on ice for 5 min before centrifugation of cell lysates at 10,000 r.p.m. at 4°C for 20 min. Supernatants were then collected. Samples were mixed with cold running buffer and loaded onto 10% SDS-PAGE, and proteins were separated at 60 V for 30 min followed by 110 V for 1.5–2 h. Gel was then transferred to a PVDF membrane (25 V, 1.3 A, 4 min), followed by 30 min incubation at RT in 0.4% PFA/PBS. Blocked with 5% nonfat dry milk powder (NFDM) in 0.1% TBST for 1 h at RT then probed with desired antibody overnight at 4C. Primary antibodies used were GFP Polyclonal Antibody, Rabbit (GenScript, A01388-40), P Histone H2A, Rabbit (Cell Signaling Technologies, 9718S), Hemoglobin Alpha Mouse monoclonal IgG1 (SantaCruz Biotechnology, sc-514378), Hemoglobin Gamma Mouse monoclonal IgG1 (SantaCruz Biotechnology, sc-21756), and Hemoglobin Beta mouse monoclonal IgG1 (SantaCruz Biotechnology, sc-21757). Membranes were washed in 0.1% TBST 3× at room temperature and secondary antibody Goat pAB to Rabbit IgG (HRP) (Abcam, ab6721) or Rabbit pAB to mouse IgG (HRP) (Abcam, ab6728) in 1:10,000 dilution in 5% NFDM. Membranes were washed 3× in 0.1% TBST, then treated with West Pico PLUS Chemiluminescent substrate (Thermo Fisher Scientific, cat. 34577) for 5 min prior to imaging on a Chemidoc MP imaging system (Bio-Rad). Relative expressions were normalized to values of hemoglobin alpha.

HPLC

Cell pellets were collected after *in vitro* erythroid differentiation of S-HUDEP2 and SCD HSPCs. For S-HUDEP2, pellets for HPLC were harvested on day 11 of erythroid differentiation. For SCD HSPCs, HPLC was performed on day 14, at the end of phase 2 of the erythroid differentiation protocol. Cells were lysed in water containing 1× phosphatase inhibitor by three freeze-thaw cycles. Lysates were cleared by centrifugation at 10,000 r.p.m. for 30 min at 4°C to remove cell membranes. Native hemoglobins were analyzed on a SmartLifeLC portable high-performance liquid chromatography system (POLYLC Inc.) using a cation-exchange PolyCAT A column. Analyte retention times were compared with AFSC hemo control (Helena Laboratories). Areas under the peaks were used to quantify

hemoglobin fractions, and the summed areas of HbA, HbF, and HbS were used for ratio comparisons.

DATA AVAILABILITY

PacBio and Illumina sequencing data have been deposited in the NCBI Sequence Read Archive (SRA) under BioProject accession number: PRJNA1260711. All other original data are available from the corresponding author upon request.

ACKNOWLEDGMENTS

This work was supported by the National Institutes of Health (R01HL169761 to G.B. and R225HL106365 to Pace B.S. with C.L.K. as a Trainee). We are grateful to the patients with sickle cell disease for permitting the use of discarded red cell exchange samples for HSPC isolation. Patients were enrolled on a protocol reviewed and approved by the Institutional Review Board at Baylor College of Medicine. NextSeq2000 sequencing was performed by the Genetic Design and Engineering Center (GDEC) at Rice University, funded by the Cancer Prevention & Research Institute of Texas (RP210116 to G.B.).

AUTHOR CONTRIBUTIONS

G.B. and S.P. conceived and directed the project; C.L.K. and S.P. planned and performed the experiments; D.B. helped with PCR, and Q.K.P. helped with flow cytometry; M.C. processed SMRT-seq data; C.L.K., S.P., and G.B. wrote the manuscript.

DECLARATION OF INTERESTS

The authors declare that they have no competing interests.

SUPPLEMENTAL INFORMATION

Supplemental information can be found online at https://doi.org/10.1016/j.omtn.2026.102854.

REFERENCES

1. Thomson, A.M., McHugh, T.A., Oron, A.P., Teply, C., Lonberg, N., Vilchis Tella, V., Wilner, L.B., Fuller, K., Hagins, H., Aboagye, R.G., et al. (2023). Global, regional, and national prevalence and mortality burden of sickle cell disease, 2000–2021: a systematic analysis from the Global Burden of Disease Study 2021. Lancet Haematol. *10*, e585–e599. https://doi.org/10.1016/s2352-3026(23)00118-7.

2. Furstenau, D.K., and Tisdale, J.F. (2021). Allogenic hematopoietic stem cell transplantation in sickle cell disease. Transfus. Apher. Sci. *60*, 103057. https://doi.org/10.1016/j.transci.2021.103057.

3. Walters, M.C., Patience, M., Leisenring, W., Eckman, J.R., Scott, J.P., Mentzer, W.C., Davies, S.C., Ohene-Frempong, K., Bernaudin, F., Matthews, D.C., et al. (1996). Bone Marrow Transplantation for Sickle Cell Disease. N. Engl. J. Med. *335*, 369–376. https://doi.org/10.1056/nejm199608083350601.

4. Leonard, A., and Tisdale, J.F. (2024). A new frontier: FDA approvals for gene therapy in sickle cell disease. Mol. Ther. *32*, 264–267. https://doi.org/10.1016/j.ymthe.2024.01.015.

5. Frangoul, H., Altshuler, D., Cappellini, M.D., Chen, Y.S., Domm, J., Eustace, B.K., Foell, J., de la Fuente, J., Grupp, S., Handgretinger, R., et al. (2021). CRISPR-Cas9 Gene Editing for Sickle Cell Disease and β-Thalassemia. N. Engl. J. Med. *384*, 252–260. https://doi.org/10.1056/NEJMoa2031054.

6. Frangoul, H., Bobruff, Y., Cappellini, M.D., Corbacioglu, S., Fernandez, C.M., de la Fuente, J., Grupp, S.A., Handgretinger, R., Ho, T.W., Imren, S., et al. (2020). Safety and Efficacy of CTX001 in Patients with Transfusion-Dependent β-Thalassemia and Sickle Cell Disease: Early Results from the Climb THAL-111 and Climb SCD-121 Studies of Autologous CRISPR-CAS9-Modified CD34+ Hematopoietic Stem and Progenitor Cells. Blood *136*, 3–4. https://doi.org/10.1182/blood-2020-139575.

7. Park, S.H., and Bao, G. (2021). CRISPR/Cas9 gene editing for curing sickle cell disease. Transfus. Apher. Sci. *60*, 103060. https://doi.org/10.1016/j.transci.2021.103060.

8. Kanter, J., DiPersio, J.F., Leavey, P., Shyr, D.C., Thompson, A.A., Porteus, M.H., Intondi, A., Lahiri, P., Dever, D.P., Petrusich, A., and Lehrer-Graiwer, J. (2021). Cedar Trial in Progress: A First in Human, Phase 1/2 Study of the Correction of a Single Nucleotide Mutation in Autologous HSCs (GPH101) to Convert HbS to HbA for Treating Severe SCD. Blood *138*, 1864. https://doi.org/10.1182/blood-2021-152892.

9. Magis, W., DeWitt, M.A., Wyman, S.K., Vu, J.T., Heo, S.J., Shao, S.J., Hennig, F., Romero, Z.G., Campo-Fernandez, B., Said, S., et al. (2022). High-level correction of the sickle mutation is amplified in vivo during erythroid differentiation. iScience *25*, 104374. https://doi.org/10.1016/j.isci.2022.104374.

10. Park, S.H., Lee, C.M., Dever, D.P., Davis, T.H., Camarena, J., Srifa, W., Zhang, Y., Paikari, A., Chang, A.K., Porteus, M.H., et al. (2019). Highly efficient editing of the β-globin gene in patient-derived hematopoietic stem and progenitor cells to treat sickle cell disease. Nucleic Acids Res. *47*, 7955–7972. https://doi.org/10.1093/nar/gkz475.

11. Lattanzi, A., Camarena, J., Lahiri, P., Segal, H., Srifa, W., Vakulskas, C.A., Frock, R.L., Kenrick, J., Lee, C., Talbott, N., et al. (2021). Development of β-globin gene correction in human hematopoietic stem cells as a potential durable treatment for sickle cell disease. Sci. Transl. Med. *13*, eabf2444. https://doi.org/10.1126/scitranslmed.abf2444.

12. Cradick, T.J., Fine, E.J., Antico, C.J., and Bao, G. (2013). CRISPR/Cas9 systems targeting β-globin and CCR5 genes have substantial off-target activity. Nucleic Acids Res. *41*, 9584–9592. https://doi.org/10.1093/nar/gkt714.

13. Yuan, B., Bi, C., Tian, Y., Wang, J., Jin, Y., Alsayegh, K., Tehseen, M., Yi, G., Zhou, X., Shao, Y., et al. (2024). Modulation of the microhomology-mediated end joining pathway suppresses large deletions and enhances homology-directed repair following CRISPR-Cas9-induced DNA breaks. BMC Biol. *22*, 101. https://doi.org/10.1186/s12915-024-01896-z.

14. Fu, Y.W., Dai, X.Y., Wang, W.T., Yang, Z.X., Zhao, J.J., Zhang, J.P., Wen, W., Zhang, F., Oberg, K.C., Zhang, L., et al. (2021). Dynamics and competition of CRISPR-Cas9 ribonucleoproteins and AAV donor-mediated NHEJ, MMEJ and HDR editing. Nucleic Acids Res. *49*, 969–985. https://doi.org/10.1093/nar/gkaa1251.

15. Park, S.H., Cao, M., Pan, Y., Davis, T.H., Saxena, L., Deshmukh, H., Fu, Y., Treangen, T., Sheehan, V.A., and Bao, G. (2022). Comprehensive analysis and accurate quantification of unintended large gene modifications induced by CRISPR-Cas9 gene editing. Sci. Adv. *8*, eabo7676. https://doi.org/10.1126/sciadv.abo7676.

16. Cazzola, M. (2022). Ineffective erythropoiesis and its treatment. Blood *139*, 2460–2470. https://doi.org/10.1182/blood.2021011045.

17. Shyr, D.C., Lowsky, R., Miller, W., Schroeder, M.A., Buchholz, T., Dougall, K., Intondi, A., Charles, A., Lehrer, J., Bouge, A., et al. (2023). One Year Follow-up on the First Patient Treated with Nula-Cel: An Autologous CRISPR/Cas9 Gene Corrected CD34+ Cell Product to Treat Sickle Cell Disease. Blood *142*, 5000. https://doi.org/10.1182/blood-2023-188963.

18. Chang, A., Park, S.H., Lee, C.M., Paikari, A., Bao, G., and Sheehan, V.A. (2018). Sickle Human Umbilical Cord Derived Erythroid Progenitor Cells (S-HUDEP2): An Ideal in-Vitro System for Screening Anti-Sickling Compounds for Sickle Cell Disease. Blood *132*, 3675. https://doi.org/10.1182/blood-2018-99-117158.

19. Deborah, E.D., Damien, J.D., Ivan, F.-V., Daniel, C.J.F., Belinda, K.S., Marieangela, C.W., Kongtana, T., Ryo, K., Yukio, N., David, J.A., et al. (2020). Comparing the two leading erythroid lines BEL-A and HUDEP-2. Haematologica *105*, e389–e394. https://doi.org/10.3324/haematol.2019.229211.

20. Kurita, R., Suda, N., Sudo, K., Miharada, K., Hiroyama, T., Miyoshi, H., Tani, K., and Nakamura, Y. (2013). Establishment of Immortalized Human Erythroid Progenitor Cell Lines Able to Produce Enucleated Red Blood Cells. PLoS One *8*, e59890. https://doi.org/10.1371/journal.pone.0059890.

21. Dewitt, M.A., Magis, W., Bray, N.L., Wang, T., Berman, J.R., Urbinati, F., Heo, S.-J., Mitros, T., Muñoz, D.P., Boffelli, D., et al. (2016). Selection-free genome editing of the sickle mutation in human adult hematopoietic stem/progenitor cells. Sci. Transl. Med. *8*, 360ra134. https://doi.org/10.1126/scitranslmed.aaf9336.

22. Hoban, M.D., Cost, G.J., Mendel, M.C., Romero, Z., Kaufman, M.L., Joglekar, A.V., Ho, M., Lumaquin, D., Gray, D., Lill, G.R., et al. (2015). Correction of the sickle cell disease mutation in human hematopoietic stem/progenitor cells. Blood *125*, 2597–2604. https://doi.org/10.1182/blood-2014-12-615948.

23. Mohrin, M., Bourke, E., Alexander, D., Warr, M.R., Barry-Holson, K., Le Beau, M.M., Morrison, C.G., and Passegué, E. (2010). Hematopoietic stem cell quiescence promotes error-prone DNA repair and mutagenesis. Cell Stem Cell *7*, 174–185. https://doi.org/10.1016/j.stem.2010.06.014.

24. Boutin, J., Rosier, J., Cappellen, D., Prat, F., Toutain, J., Pennamen, P., Bouron, J., Rooryck, C., Merlio, J.P., Lamrissi-Garcia, I., et al. (2021). CRISPR-Cas9 globin editing can induce megabase-scale copy-neutral losses of heterozygosity in hematopoietic cells. Nat. Commun. *12*, 4922. https://doi.org/10.1038/s41467-021-25190-6.

25. Dever, D.P., Bak, R.O., Reinisch, A., Camarena, J., Washington, G., Nicolas, C.E., Pavel-Dinu, M., Saxena, N., Wilkens, A.B., Mantri, S., et al. (2016). CRISPR/Cas9 β-globin gene targeting in human haematopoietic stem cells. Nature *539*, 384–389. https://doi.org/10.1038/nature20134.

26. Vakulskas, C.A., Dever, D.P., Rettig, G.R., Turk, R., Jacobi, A.M., Collingwood, M.A., Bode, N.M., McNeill, M.S., Yan, S., Camarena, J., et al. (2018). A high-fidelity Cas9 mutant delivered as a ribonucleoprotein complex enables efficient gene editing in human hematopoietic stem and progenitor cells. Nat. Med. *24*, 1216–1224. https://doi.org/10.1038/s41591-018-0137-0.

27. Kosicki, M., Allen, F., Steward, F., Tomberg, K., Pan, Y., and Bradley, A. (2022). Cas9-induced large deletions and small indels are controlled in a convergent fashion. Nat. Commun. *13*, 3422. https://doi.org/10.1038/s41467-022-30480-8.

28. Wen, W., Quan, Z.-J., Li, S.-A., Yang, Z.-X., Fu, Y.-W., Zhang, F., Li, G.-H., Zhao, M., Yin, M.-D., Xu, J., et al. (2021). Effective control of large deletions after double-strand breaks by homology-directed repair and dsODN insertion. Genome Biol. *22*, 236. https://doi.org/10.1186/s13059-021-02462-4.

29. Fu, Y.-W., Dai, X.-Y., Wang, W.-T., Yang, Z.-X., Zhao, J.-J., Zhang, J.-P., Wen, W., Zhang, F., Oberg, K.C., Zhang, L., et al. (2021). Dynamics and competition of CRISPR–Cas9 ribonucleoproteins and AAV donor-mediated NHEJ, MMEJ and HDR editing. Nucleic Acids Res. *49*, 969–985. https://doi.org/10.1093/nar/gkaa1251.

30. Schimmel, J., Muñoz-Subirana, N., Kool, H., Van Schendel, R., Van Der Vlies, S., Kamp, J.A., De Vrij, F.M.S., Kushner, S.A., Smith, G.C.M., Boulton, S.J., and Tijsterman, M. (2023). Modulating mutational outcomes and improving precise gene editing at CRISPR-Cas9-induced breaks by chemical inhibition of end-joining pathways. Cell Rep. *42*, 112019. https://doi.org/10.1016/j.celrep.2023.112019.

31. Caulier, A.L., and Sankaran, V.G. (2022). Molecular and cellular mechanisms that regulate human erythropoiesis. Blood *139*, 2450–2459. https://doi.org/10.1182/blood.2021011044.

32. Antoniani, C., Meneghini, V., Lattanzi, A., Felix, T., Romano, O., Magrin, E., Weber, L., Pavani, G., El Hoss, S., Kurita, R., et al. (2018). Induction of fetal hemoglobin synthesis by CRISPR/Cas9-mediated editing of the human β-globin locus. Blood *131*, 1960–1973. https://doi.org/10.1182/blood-2017-10-811505.

33. Pavani, G., Fabiano, A., Laurent, M., Amor, F., Cantelli, E., Chalumeau, A., Maule, G., Tachtsidi, A., Concordet, J.-P., Cereseto, A., et al. (2021). Correction of β-thalassemia by CRISPR/Cas9 editing of the α-globin locus in human hematopoietic stem cells. Blood Adv. *5*, 1137–1153. https://doi.org/10.1182/bloodadvances.2020001996.

34. Canver, M.C., Smith, E.C., Sher, F., Pinello, L., Sanjana, N.E., Shalem, O., Chen, D.D., Schupp, P.G., Vinjamur, D.S., Garcia, S.P., et al. (2015). BCL11A enhancer dissection by Cas9-mediated in situ saturating mutagenesis. Nature *527*, 192–197. https://doi.org/10.1038/nature15521.

35. Masuda, T., Wang, X., Maeda, M., Canver, M.C., Sher, F., Funnell, A.P.W., Fisher, C., Suciu, M., Martyn, G.E., Norton, L.J., et al. (2016). Transcription factors LRF and BCL11A independently repress expression of fetal hemoglobin. Science *351*, 285–289. https://doi.org/10.1126/science.aad3312.

36. Verheul, T.C.J., Gillemans, N., Putzker, K., Majied, R., Li, T., Vasiliou, M., Eussen, B., De Klein, A., Van Ijcken, W.F.J., Van Den Akker, E., et al. (2024). A cellular reporter system to evaluate endogenous fetal hemoglobin induction and screen for therapeutic compounds. HemaSphere *8*, e139. https://doi.org/10.1002/hem3.139.

37. Khandros, E., and Blobel, G.A. (2024). Elevating fetal hemoglobin: recently discovered regulators and mechanisms. Blood *144*, 845–852. https://doi.org/10.1182/blood.2023022190.

38. Glaser, A., McColl, B., and Vadolas, J. (2016). GFP to BFP Conversion: A Versatile Assay for the Quantification of CRISPR/Cas9-mediated Genome Editing. Mol. Ther. Nucleic Acids *5*, e334. https://doi.org/10.1038/mtna.2016.48.

39. Neu-Yilik, G., Amthor, B., Gehring, N.H., Bahri, S., Paidassi, H., Hentze, M.W., and Kulozik, A.E. (2011). Mechanism of escape from nonsense-mediated mRNA decay of human β-globin transcripts with nonsense mutations in the first exon. RNA *17*, 843–854. https://doi.org/10.1261/rna.2401811.

40. Romão, L., Inácio, A., Santos, S., Avila, M., Faustino, P., Pacheco, P., and Lavinha, J. (2000). Nonsense mutations in the human beta-globin gene lead to unexpected levels of cytoplasmic mRNA accumulation. Blood *96*, 2895–2901.

41. Neu-Yilik, G., Amthor, B., Gehring, N.H., Bahri, S., Paidassi, H., Hentze, M.W., and Kulozik, A.E. (2011). Mechanism of escape from nonsense-mediated mRNA decay of human beta-globin transcripts with nonsense mutations in the first exon. RNA *17*, 843–854. https://doi.org/10.1261/rna.2401811.

42. Joly, E. (2007). Optimising Blue Fluorescent Protein (BFP) for use as a mammalian reporter gene in parallel with Green Fluorescent Protein (GFP). Nat. Prec. https://doi.org/10.1038/npre.2007.1259.1.

43. Eid, J., Fehr, A., Gray, J., Luong, K., Lyle, J., Otto, G., Peluso, P., Rank, D., Baybayan, P., Bettman, B., et al. (2009). Real-Time DNA Sequencing from Single Polymerase Molecules. Science *323*, 133–138. https://doi.org/10.1126/science.1162986.

44. Huang, P., Keller, C.A., Giardine, B., Grevet, J.D., Davies, J.O.J., Hughes, J.R., Kurita, R., Nakamura, Y., Hardison, R.C., and Blobel, G.A. (2017). Comparative analysis of three-dimensional chromosomal architecture identifies a novel fetal hemoglobin regulatory element. Genes Dev. *31*, 1704–1713. https://doi.org/10.1101/gad.303461.117.

45. Chung, J.E., Magis, W., Vu, J., Heo, S.-J., Wartiovaara, K., Walters, M.C., Kurita, R., Nakamura, Y., Boffelli, D., Martin, D.I.K., et al. (2019). CRISPR-Cas9 interrogation of a putative fetal globin repressor in human erythroid cells. PLoS One *14*, e0208237. https://doi.org/10.1371/journal.pone.0208237.

46. Traxler, E.A., Yao, Y., Wang, Y.-D., Woodard, K.J., Kurita, R., Nakamura, Y., Hughes, J.R., Hardison, R.C., Blobel, G.A., Li, C., and Weiss, M.J. (2016). A genome-editing strategy to treat β-hemoglobinopathies that recapitulates a mutation associated with a benign genetic condition. Nat. Med. *22*, 987–990. https://doi.org/10.1038/nm.4170.

47. Huang, P., Peslak, S.A., Ren, R., Khandros, E., Qin, K., Keller, C.A., Giardine, B., Bell, H.W., Lan, X., Sharma, M., et al. (2022). HIC2 controls developmental hemoglobin switching by repressing BCL11A transcription. Nat. Genet. *54*, 1417–1426. https://doi.org/10.1038/s41588-022-01152-6.

48. Topfer, S.K., Feng, R., Huang, P., Ly, L.C., Martyn, G.E., Blobel, G.A., Weiss, M.J., Quinlan, K.G.R., and Crossley, M. (2022). Disrupting the adult globin promoter alleviates promoter competition and reactivates fetal globin gene expression. Blood *139*, 2107–2118. https://doi.org/10.1182/blood.2021014205.

49. Peixeiro, I., Silva, A.L., and Romão, L. (2011). Control of human beta-globin mRNA stability and its impact on beta-thalassemia phenotype. Haematologica *96*, 905–913. https://doi.org/10.3324/haematol.2010.039206.

50. Chakalova, L., Osborne, C.S., Dai, Y.F., Goyenechea, B., Metaxotou-Mavromati, A., Kattamis, A., Kattamis, C., and Fraser, P. (2005). The Corfu deltabeta thalassemia deletion disrupts gamma-globin gene silencing and reveals post-transcriptional regulation of HbF expression. Blood *105*, 2154–2160. https://doi.org/10.1182/blood-2003-11-4069.

51. Mabaera, R., West, R.J., Conine, S.J., Macari, E.R., Boyd, C.D., Engman, C.A., and Lowrey, C.H. (2008). A cell stress signaling model of fetal hemoglobin induction: what doesn't kill red blood cells may make them stronger. Exp. Hematol. *36*, 1057–1072. https://doi.org/10.1016/j.exphem.2008.06.014.

52. Ribeil, J.-A., Arlet, J.-B., Dussiot, M., Moura, I.C., Courtois, G., and Hermine, O. (2013). Ineffective Erythropoiesis in *β*-Thalassemia. Sci. World J. *2013*, 394295. https://doi.org/10.1155/2013/394295.

53. Rivella, S. (2009). Ineffective erythropoiesis and thalassemias. Curr. Opin. Hematol. *16*, 187–194. https://doi.org/10.1097/moh.0b013e32832990a4.

54. Cullot, G., Aird, E.J., Schlapansky, M.F., Yeh, C.D., Van De Venn, L., Vykhlyantseva, I., Kreutzer, S., Mailänder, D., Lewków, B., Klermund, J., et al. (2025). Genome editing with the HDR-enhancing DNA-PKcs inhibitor AZD7648 causes large-scale genomic alterations. Nat. Biotechnol. *43*, 1778–1782. https://doi.org/10.1038/s41587-024-02488-6.

55. Regan, S.B., Medhi, D., White, T.B., Jiang, Y.Z., Jia, S., Deng, Q., and Jasin, M. (2025). Megabase-scale loss of heterozygosity provoked by CRISPR-Cas9 DNA double-strand breaks. Mol. Cell *22*, 4119–4137. https://doi.org/10.1016/j.molcel.2025.10.015.

56. Wang, J., Sadeghi, C.A., and Frock, R.L. (2024). DNA-PKcs suppresses illegitimate chromosome rearrangements. Nucleic Acids Res. *52*, 5048–5066. https://doi.org/10.1093/nar/gkae140.

57. Jin, Y.-Y., Zhang, P., Liu, L.-L., Zhao, X., Hu, X.-Q., Liu, S.-Z., Li, Z.-K., Liu, Q., Wang, J.-Q., Hao, D.-L., et al. (2024). Enhancing homology-directed repair efficiency with HDR-boosting modular ssDNA donor. Nat. Commun. *15*, 6843. https://doi.org/10.1038/s41467-024-50788-x.

58. Riesenberg, S., Chintalapati, M., Macak, D., Kanis, P., Maricic, T., and Pääbo, S. (2019). Simultaneous precise editing of multiple genes in human cells. Nucleic Acids Res. *47*, e116. https://doi.org/10.1093/nar/gkz669.

59. Panier, S., and Boulton, S.J. (2014). Double-strand break repair: 53BP1 comes into focus. Nat. Rev. Mol. Cell Biol. *15*, 7–18. https://doi.org/10.1038/nrm3719.

60. Glenn S. Alt-R HDR Enhancer Protein: Improving homology-directed repair from bench to bedside Integrated DNA Technologies2025. Available from: https://www.idtdna.com/page/support-and-education/decoded-plus/alt-r-hdr-enhancer-protein-improving-homology-directed-repair-from-bench-to-bedside/

61. Hwang, G.-H., Lee, S.-H., Oh, M., Kim, S., Habib, O., Jang, H.-K., Kim, H.S., Kim, Y., Kim, C.H., Kim, S., and Bae, S. (2025). Large DNA deletions occur during DNA repair at 20-fold lower frequency for base editors and prime editors than for Cas9 nucleases. Nat. Biomed. Eng. *9*, 79–92. https://doi.org/10.1038/s41551-024-01277-5.

62. Turchiano, G., Andrieux, G., Klermund, J., Blattner, G., Pennucci, V., El Gaz, M., Monaco, G., Poddar, S., Mussolino, C., Cornu, T.I., et al. (2021). Quantitative evaluation of chromosomal rearrangements in gene-edited human stem cells by CAST-Seq. Cell Stem Cell *28*, 1136–1147.e5. https://doi.org/10.1016/j.stem.2021.02.002.

63. FDA (2024). Human Gene Therapy Products Incorporating Human Genome Editing: Guidance for Industry (Maryland: FDA). https://www.fda.gov/regulatory-information/search-fda-guidance-documents/human-gene-therapy-products-incorporating-human-genome-editing.

64. Kosicki, M., Rajan, S.S., Lorenzetti, F.C., Wandall, H.H., Narimatsu, Y., Metzakopian, E., and Bennett, E.P. (2017). Dynamics of Indel Profiles Induced by Various CRISPR/Cas9 Delivery Methods. Prog. Mol. Biol. Transl. Sci. *152*, 49–67. https://doi.org/10.1016/bs.pmbts.2017.09.003.

65. Trakarnsanga, K., Griffiths, R.E., Wilson, M.C., Blair, A., Satchwell, T.J., Meinders, M., Cogan, N., Kupzig, S., Kurita, R., Nakamura, Y., et al. (2017). An immortalized adult human erythroid line facilitates sustainable and scalable generation of functional red cells. Nat. Commun. *8*, 14750. https://doi.org/10.1038/ncomms14750.

66. Sharma, A., Boelens, J.J., Cancio, M., Hankins, J.S., Bhad, P., Azizy, M., Lewandowski, A., Zhao, X., Chitnis, S., Peddinti, R., et al. (2023). CRISPR-Cas9 Editing of the HBG1 and HBG2 Promoters to Treat Sickle Cell Disease. N. Engl. J. Med. *389*, 820–832. https://doi.org/10.1056/NEJMoa2215643.

67. Métais, J.Y., Doerfler, P.A., Mayuranathan, T., Bauer, D.E., Fowler, S.C., Hsieh, M.M., Katta, V., Keriwala, S., Lazzarotto, C.R., Luk, K., et al. (2019). Genome editing of HBG1 and HBG2 to induce fetal hemoglobin. Blood Adv. *3*, 3379–3392. https://doi.org/10.1182/bloodadvances.2019000820.

68. Hanna, R., Frangoul, H., McKinney, C., Pineiro, L., Mapara, M., Dalal, J., Rangarajan, H., Atkins, H., Chang, K.-H., Mei, B., et al. (2024). Reni-Cel, an Investigational AsCas12a Gene-Edited Cell Medicine, Led to Sustained Hemoglobin Normalization and Increased Fetal Hemoglobin in Patients with Severe Sickle Cell Disease Treated in the RUBY Trial. Blood *144*, 4955. https://doi.org/10.1182/blood-2024-210343.

69. Liao, J., Chen, S., Hsiao, S., Jiang, Y., Yang, Y., Zhang, Y., Wang, X., Lai, Y., Bauer, D.E., and Wu, Y. (2023). Therapeutic adenine base editing of human hematopoietic stem cells. Nat. Commun. *14*, 207. https://doi.org/10.1038/s41467-022-35508-7.

70. Xu, S., Liang, D., Wang, Q., Cheng, Y., Xie, D., Gui, Y., Zhang, H., Feng, C., Zhao, F., Ren, W., et al. (2025). In vivo genome editing of human haematopoietic stem cells for treatment of blood disorders using mRNA delivery. Nat. Biomed. Eng. https://doi.org/10.1038/s41551-025-01480-y.

71. Pinello, L., Canver, M.C., Hoban, M.D., Orkin, S.H., Kohn, D.B., Bauer, D.E., and Yuan, G.-C. (2016). Analyzing CRISPR genome-editing experiments with CRISPResso. Nat. Biotechnol. *34*, 695–697. https://doi.org/10.1038/nbt.3583.

OMTN, Volume *37*

Supplemental information

Complex HBB gene editing outcomes revealed by a fluorescent reporter cell model

Cecile L. Karsenty, Daniel Betancourth, Mingming Cao, Quoc-Khanh Pham, So Hyun Park, and Gang Bao

SUPPLEMENTAL INFORMATION

Supplemental Tables

Table S1. Oligonucleotide Sequences:

**sgRNA protospacer sequences**		**Relevant figures**
R-66S sgRNA	GUAACGGCAGACUUCUCCAC	Figure 1-6, S1, S5, S9, S14, S24, S26, S27, S28, S30, S31
R-02 sgRNA	CUUGCCCCACAGGGCAGUAA	Figure 1-6, S1, S5, S9, S14, S26, S30
R-62 sgRNA	CGUGGAUGAAGUUGGUGGUG	Figure 2, S9, S10
MET-55 sgRNA	UCCACUCCUGAUGCUGUUAU	Figure 2, S9, S10
HBB_c terminus sgRNA	AGCGAGCUUAGUGAUACUUG	Figure 2
GFP sgRNA	GCUGAAGCACUGCACGCCGU	Figure 2
**Donors**		
GFP	CAGGAAACAGCTATGACAAGCTTTTAATATACTTTTTTGTTTATCTTATTTCTAATACTTTCCCTAAT<br>CTCTTTCTTTCAGGGCAATAATGATACAATGTATCATGCCTCTTTGCACCATTCTAAAGAATAACAG<br>TGATAATTTCTGGGTTAAGGCAATAGCAATATCTCTGCATATAAATATTTCTGCATATAAATTGTAAC<br>TGATGTAAGAGGTTTCATATTGCTAATAGCAGCTACAATCCAGCTACCATTCTGCTTTTATTTTATG<br>GTTGGGATAAGGCTGGATTATTCTGAGTCCAAGCTAGGCCCTTTTGCTAATCATGTTCATACCTCT<br>TATCTTCCTCCCACAGCTCCTGGGCAACGTGCTGGTCTGTGTGCTGGCCCATCACTTTGGCAAAG<br>AATTCACCCCACCAGTGCAGGCTGCCTATCAGAAAGTGGTGGCTGGTGTGGCTAATGCCCTGGC<br>CCACAAGTATCACGGATCCGGCGCAACAAACTTCTCTCTGCTGAAACAAGCCGGAGATGTCGAA<br>GAGAATCCTGGACCGATGGTGTCTAAGGGCGAGGAGCTGTTCACCGGGGTGGTGCCCATCCTG<br>GTCGAGCTGGACGGCGACGTAAACGGCCACAAGTTCAGCGTGTCCGGCGAGGGCGAGGGCG<br>ATGCCACCTACGGCAAGCTGACCCTGAAGTTCATCTGCACCACCGGCAAGCTGCCCGTGCCCTG<br>GCCTACCCTGGTGACCACCCTGACCTACGGCGTGCAGTGCTTCAGCCGCTACCCCGACCACATG<br>AAGCAGCACGACTTCTTCAAGTCCGCCATGCCCGAAGGCTACGTCCAGGAGCGCACCATCTTCT<br>TCAAGGACGACGGCAACTACAAGACCCGCGCCGAGGTGAAGTTCGAGGGCGACACCCTGGTG<br>AACCGCATCGAGCTGAAGGGCATCGACTTCAAGGAGGACGGCAACATCCTGGGGCACAAGCT<br>GGAGTACAACTACAACAGCCACAACGTCTATATCATGGCCGACAAGCAGAAGAACGGCATCAA<br>GGTGAACTTCAAGATCCGCCACAACATCGAGGACGGCAGCGTGCAGCTCGCCGACCACTACCA<br>GCAGAACACCCCCATCGGCGACGGCCCCGTGCTGCTGCCCGACAACCACTACCTGAGCACCCA<br>GTCCGCCCTGAGCAAAGACCCCAACGAGAAGCGCGATCACATGGTCCTGCTGGAGTTCGTGAC<br>CGCCGCCGGGATCACTCTCGGCATGGACGAGCTGTACAAGTAACCTGAGGATCCGATCTTTTTC<br>CCTCTGCCAAAAATTATGGGGACATCATGAAGCCCCTTGAGCATCTGACTTCTGGCTAATAAAGG<br>AAATTTATTTTCATTGCAATAGTGTGTTGGAATTTTTTGTGTCTCTCAGTATCACTAAGCTCGCTTT<br>CTTGCTGTCCAATTTCTATTAAAGGTTCCTTTGTTCCCTAAGTCCAACTACTAAACTGGGGGATAT<br>TATGAAGGGCCTTGAGCATCTGGATTCTGCCTAATAAAAAACATTTATTTTCATTGCAATGATGTA<br>TTTAAATGGTACCACTGGCCGTCGTTTTACA	Figure 2
GFP>BFP ssODN	AAGCTGCCCGTGCCCTGGCCTACCCTGGTGACCACCCTGAGCCATGGGGTGCAGTGCTTCAGCC<br>GCTACCCCGACCACATGAAGCAGC	Figure 2
R-66S ssODN (for SCD HSPCs)	TCTGACACAACTGTGTTCACTAGCAACCTCAAACAGACACCATGGTGCACCTGACTCCTGAAGA<br>GAAGTCTGCCGTTACTGCCCTGTGGGGCAAGGTGAACGTGGATGAAGTTGGTGGTGA	Figure 1,6, S5, S26
R-66S ssODN (for S-HUDEP2)	TCTGACACAACTGTGTTCACTAGCAACCTCAAACAGACACCATGGTGCATCTGACTCCTGAAGA<br>GAAGTCTGCCGTTACTGCCCTGTGGGGCAAGGTGAACGTGGATGAAGTTGGTGGTGA	Figure 2,3,5,6, S14, S24, S30
R-02 ssODN (for SCD HSPCs)	TCAGGGCAGAGCCATCTATTGCTTACATTTGCTTCTGACACAACTGTGTTCACTAGCAACCTCA<br>AACAGACACCATGGTGCACCTGACTCCTGAAGAGAAGTCTGCGGTTACTGCCCTGTGGGGCA<br>AGGTGAACGTGGATGAAGTTGGTGGTGAGGCCCTGGGCAGGT	Figure 1,6, S5, S26
R-02 ssODN (for S-HUDEP2)	TCAGGGCAGAGCCATCTATTGCTTACATTTGCTTCTGACACAACTGTGTTCACTAGCAACCTCAA<br>ACAGACACCATGGTGCATCTGACTCCTGAAGAGAAGTCTGCGGTTACTGCCCTGTGGGGCAAG<br>GTGAACGTGGATGAAGTTGGTGGTGAGGCCCTGGGCAGGT	Figure 2,3,5,6, S14, S30

**Primers and probes**		
NGS HBB	F- TGGCCAATCTACTCCCAGGA<br>R- CTGTCTCCACATGCCCAGTT	Figure 1, 3, 6 S7, S26, S27, S31
NGS Met 55	F- TGGGCATGTGGAGACAGAGA<br>R- AGCTCACTCAGTGTGGCAAA	Figure S7
ddPCR	F- CTCCACATGCCCAGTTTCTA<br>R- GCAACCTCAAACAGACACCA<br>Probe sequences<br>HBB ref (ROX)- ACGTGGATGAAGTTGGTGGTGAGG<br>RPP30 reference (VIC)- CTGACCTGAAGGCTCT	Figure 1, 3, 6, S22
qPCR	F (GFP/BFP)- ATGGTGTCTAAGGGCGAGGA<br>R (GFP)- GAAGCACTGCACCCCGTAGG<br>R (BFP)- GAAGCACTGCACCCCATGGC<br>F (HBB)- GTCCACTCCTGATGCTGTTATG<br>R (HBB)- GGCACCGAGCACTTTCTTG	Figure S19
cDNA NGS	F (HBB)- GGACCCAGAGGTTCTTTG<br>R (HBB)- GGGGTGAATTCTTTGCC<br>F (GFP/BFP)- CGTAAACGGCCACAAGTTCA<br>R (GFP/BFP)- CTTGTAGTTGCCGTCGTCCT	Figure S19
SMRT-seq PCR1	F- CAAGCAGAAGACGGCATACGAGATCACGTGTGCCTAGATCCTCA<br>R- AATGATACGGCGACCACCGAGATCCTGATTCTCCCACCCCCAAC	Figure 4, S16, S17

Supplemental Figures

A. R-66S RNP

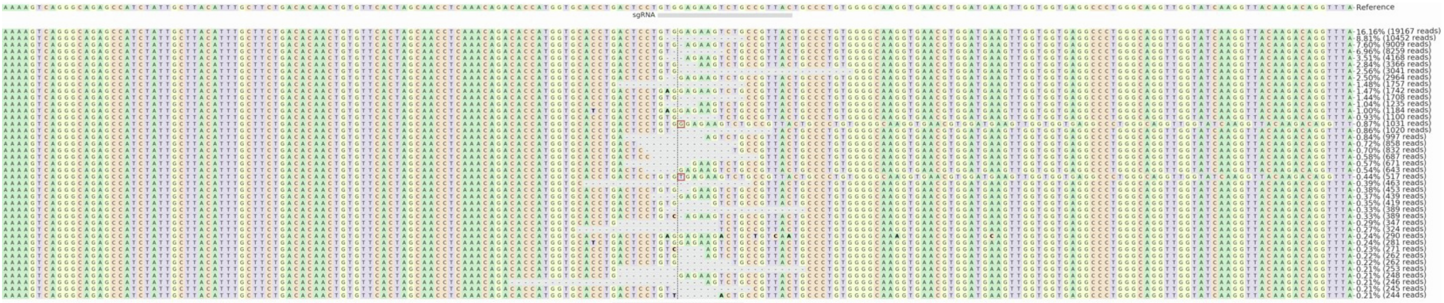

B. R-02 RNP

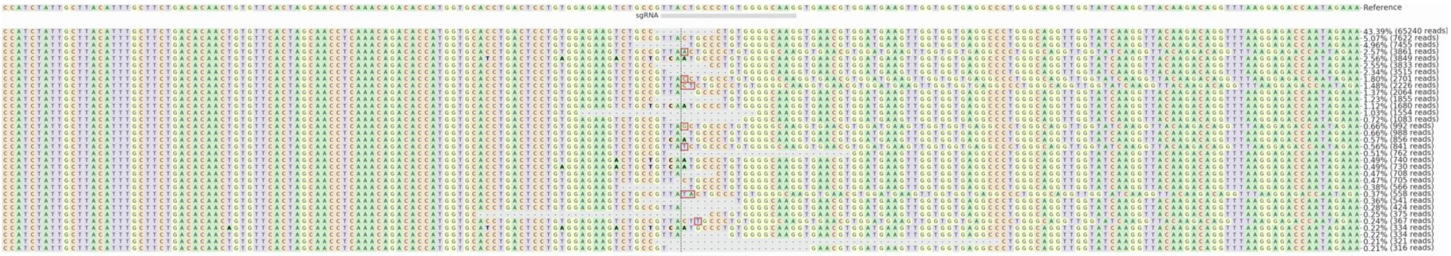

Figure S1. gRNA specific indel profiles seen after R-66S RNP and R-02 RNP electroporation of patient derived HSPCs. Summary of major indels generated by RNP electroporation of donor 1 HSPCs generated by NGS and CRISPResso2 analysis. gRNA specific indel profile seen when comparing (**A**) R-66S gRNA and (**B**) R-02 gRNA.

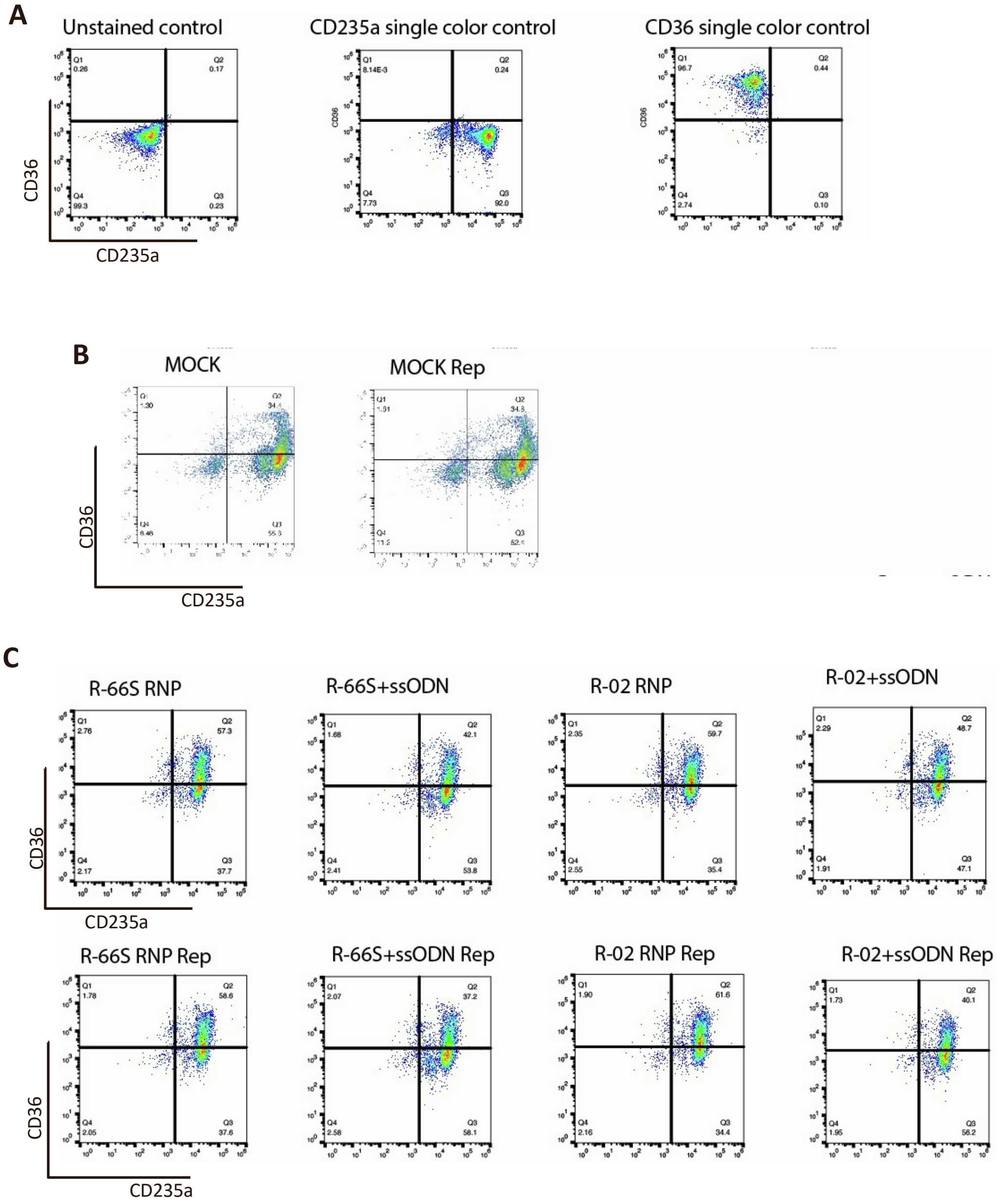

Figure S2. Therapeutic gene editing targeting the sickle mutation in vitro affects erythroid differentiation. Using SCD HSPCs from donor 1, R-66S RNP, R-02 RNP, R-66S+ssODN, R-02+ssODN, R-62 RNP, and Met-55 RNP were delivered via electroporation and followed by in vitro erythroid differentiation with n=2 biological replicates. On day 15 of differentiation, the impact of gene editing on maturation was assessed using erythroid-specific markers (CD36 and CD235a) and flow cytometry. Results demonstrate delayed erythropoiesis after RNP treatment based on increased percentage of double positive (CD36+/CD345a+) cells. (**A**) A gating strategy using unstained control and single-color controls for CD36 and CD235a staining on the Sony MA900 flow cytometer. (**B**) Day15, CD36 and CD235a staining for mock edited control (**C**) CD36 and CD235a positivity on day 15 of differentiation for R-66S RNP, R-66S RNP + ssODN, R-02 RNP and R-02 RNP + ssODN. The RNP-only-treated group showed delayed maturation compared with the mock- and RNP + ssODN-treated groups, with a higher proportion of immature ($CD36^+/CD235a^+$) cells. The addition of a corrective ssODN donor restores maturation.

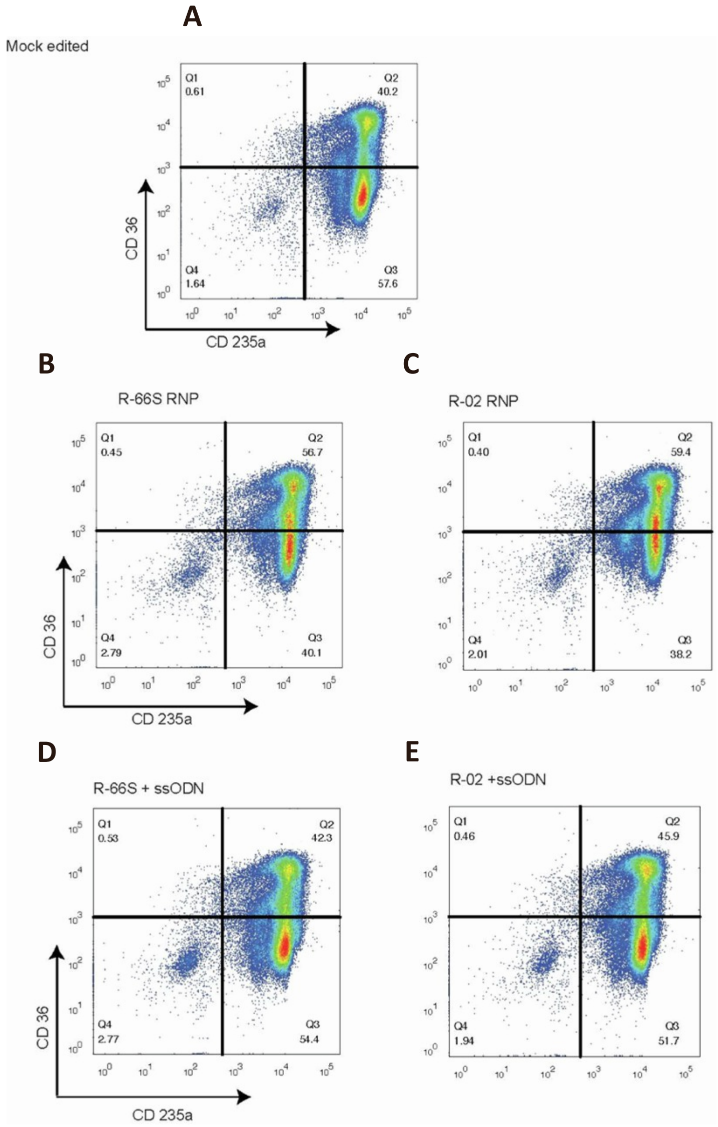

Figure S3. Therapeutic gene editing targeting the sickle mutation in vitro affects erythroid differentiation. *In vitro* erythroid maturation (13-day) of donor 2 SCD HSPCs showed delayed erythropoiesis after RNP treatment, with a higher proportion of immature (CD36+/CD235a+) cells compared to mature (CD36-/CD235a+). **(A)** Day 13, CD36 and CD235a staining for mock-edited control. CD36 and CD235a positivity on day 13 of differentiation for **(B)** R-66S RNP treated and **(C)** R-02 RNP treated groups, demonstrating a delay in maturation when compared to mock. and CD36 and CD235a positivity on day 13 of erythroid differentiation for **(D)** R-66S RNP + ssODN and **(E)** R-02 RNP + ssODN treated samples. Addition of corrective ssODN donor rescues maturation.

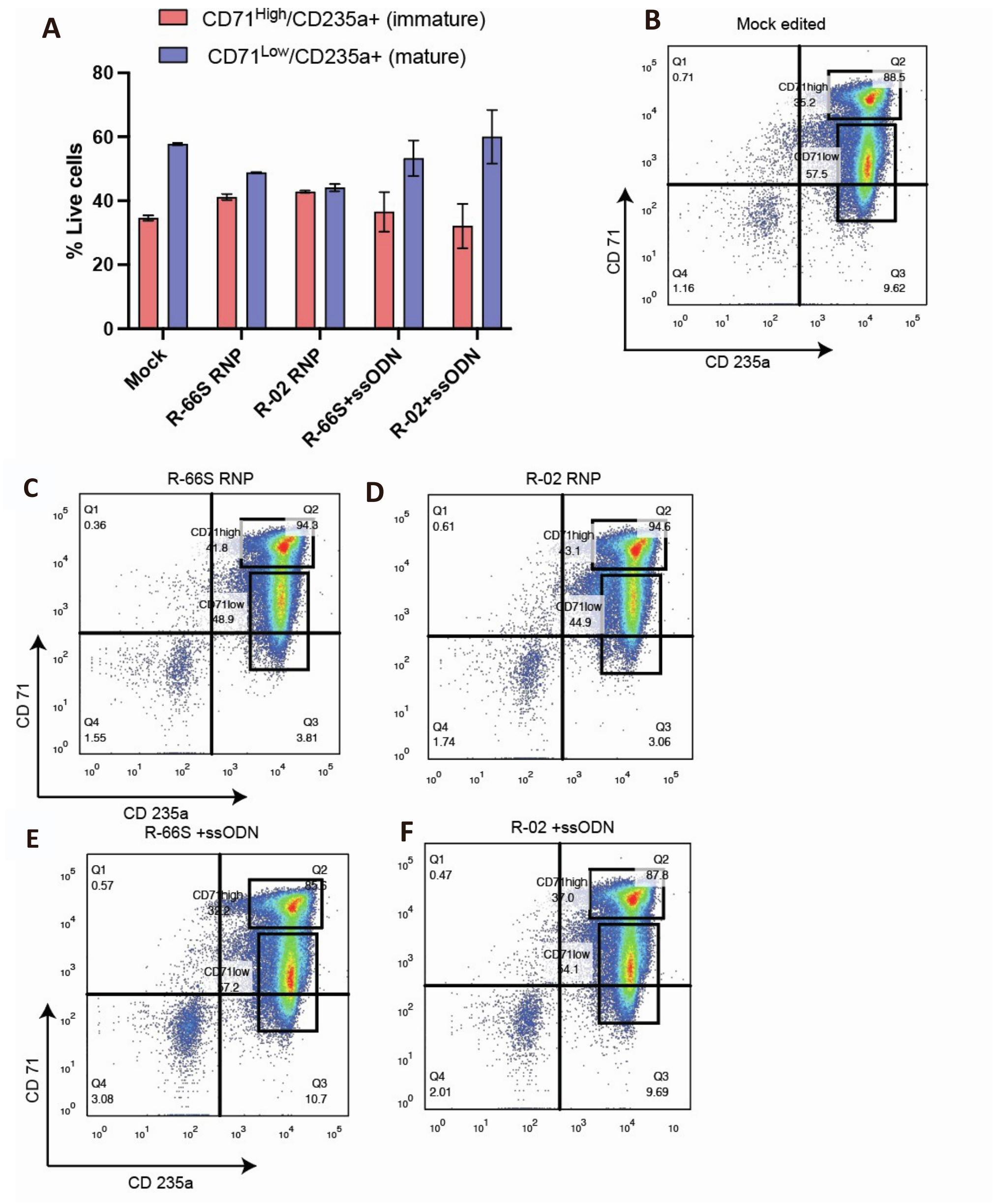

Figure S4. Therapeutic gene editing targeting SCD mutation *in vitro* impacts erythroid differentiation, as assessed by CD71 and CD235a expression. (**A**) *In vitro* erythroid maturation (13-day) of donor 2 SCD HSPCs showed delayed erythropoiesis after RNP treatment, with a higher proportion of immature (CD71+/CD235a+) cells compared to mature (CD71-/CD235a+); n=2 biological replicates. (**B-F**) Flow cytometry plots assessing CD71 and CD235a positivity after *in vitro* differentiation (13 days) of (**B**) mock treated control. (**C**) R-66S RNP treated cells, (**D**) R-02 RNP treated cells. (**E**) R-66S RNP + ssODN treated cells. (**F**) R-02 RNP + ssODN treated cells. Delayed maturation is observed with RNP treatment and is rescued by the addition of a corrective ssODN donor, compared with mock-treated cells.

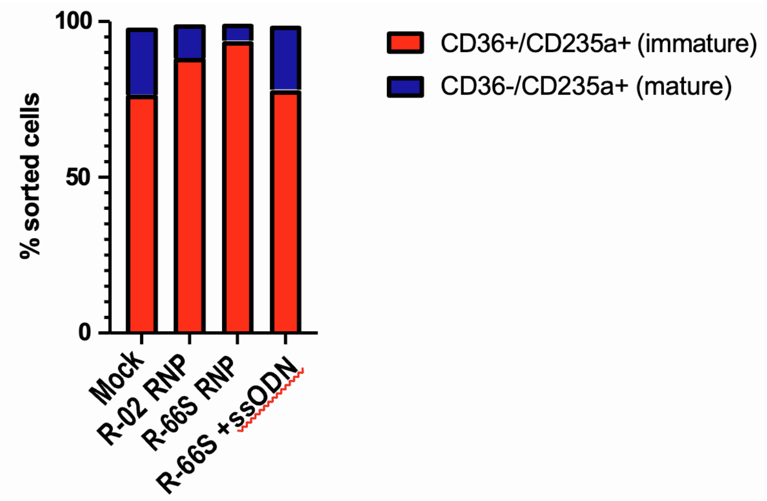

Figure S5. RNP delivery and efficient CRISPR editing result in a higher percentage of cells with an immature phenotype, as assessed by flow cytometry, due to the underlying genotypes. In HSPCs derived from donor 3, RNP and RNP+ssODN were delivered via electroporation using R-66s and R-02 targeting the sickle mutation. After 10 days of *in vitro* erythroid differentiation, cells were stained for CD36 and CD235a. As in donors 1 and 2, there is a clear increase in the percentage of cells with an immature phenotype in the RNP-treated groups compared to the mock-edited control. Using FACS, immature (CD36^{+}/CD235a$^{+)}$ and mature (CD36^{-}/CD235a^{+}) SCD HSPCs from donor 3 were bulk sorted for LongAmp-seq analysis.

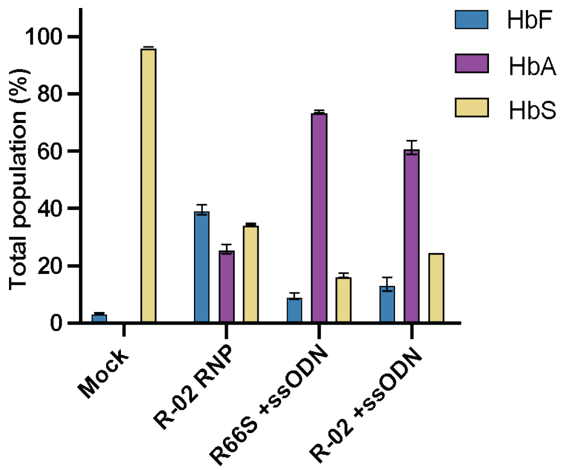

Figure S6. HPLC analysis and quantification of HbF, HbA, and HbS after therapeutic gene editing in patient-derived HSPCs. HPLC analysis and quantification of measurable HbF, HbA, and HbS demonstrate near-100 % HbS production in mock-treated controls. Loss of HbS is seen with R-02 RNP delivery, with apparent mild HbA induction due to HBD conversion occurring with RNP delivery alone. HbF induction is seen with RNP delivery alone. Additionally, RNP delivery resulted in several nonspecific peaks within the HPLC window, which are not shown here. Addition of corrective donor results in a significant increase in HbA with minimal residual HbS. N=2 technical replicates.

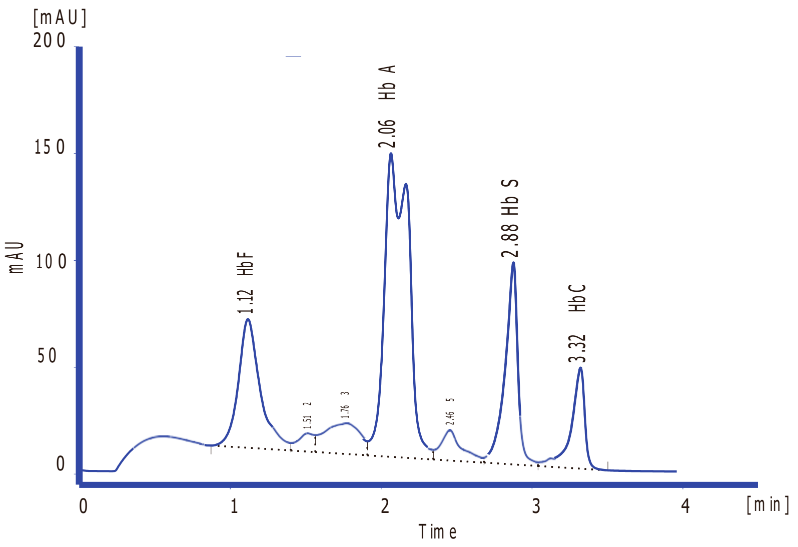

Figure S7. HPLC reference control demonstrating reference migration patterns and retention times for hemoglobin F, A, S, and C.

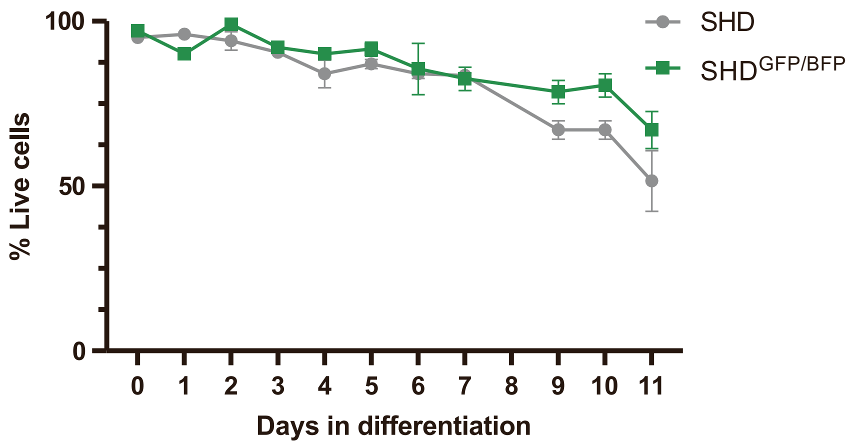

Figure S8. SHD gene tagging does not affect viability or the ability to undergo in vitro erythroid differentiation. The viability of parental S-HUDEP2 and SHD$^{GFP/BFP}$ models is compared throughout in vitro differentiation using trypan blue staining and shows a similar trend. No significant differences were observed; SHD$^{GFP/BFP}$ maintained viability compared to parental S-HUDEP2.

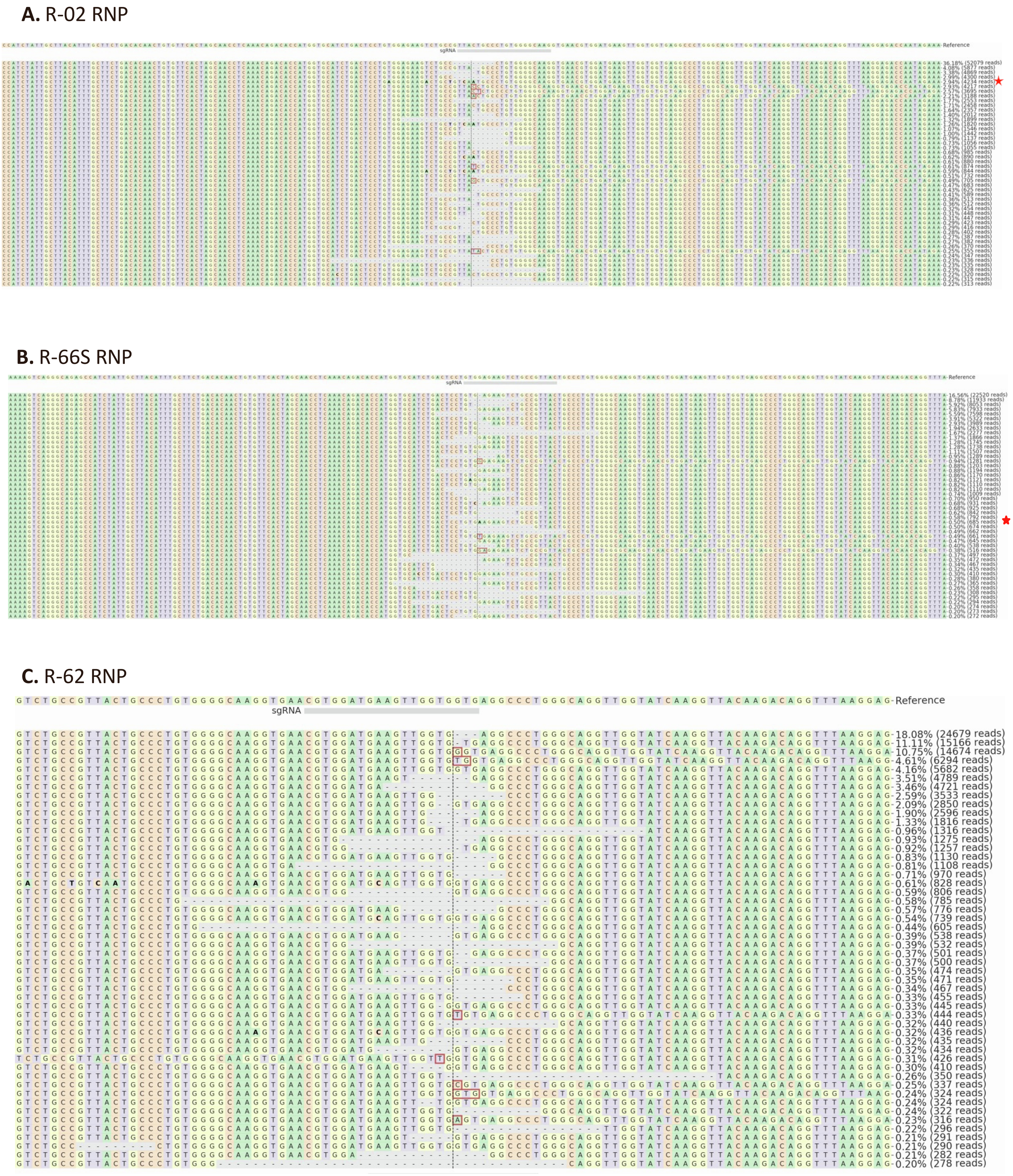
A. R-02 RNP
B. R-66S RNP
C. R-62 RNP
Reference
sgRNA

D. Met-55 RNP

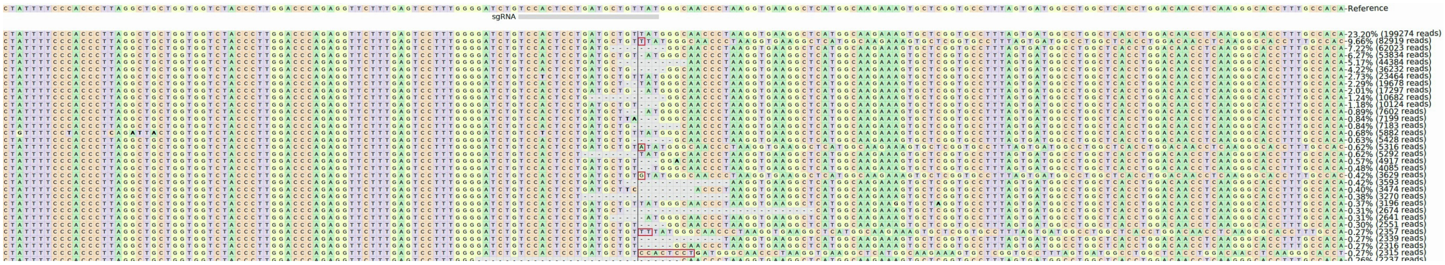

Figure S9. Unique indel patterns after R-66S RNP, R-02 RNP, R-62 RNP, and Met-55 RNP mediated editing in SHD$^{GFP/BFP}$. CRISPResso2 NGS analysis for each gRNA demonstrates unique indel patterns at the on-target cut site. Summary of the most frequent indels in the bulk population for each gRNA is shown. Red stars indicate alleles that have undergone HBD conversion due to R-66S and R-02 editing. This results in a T-to-A correction, which can repair the SCD mutation in the chimeric HBB-HBD gene.

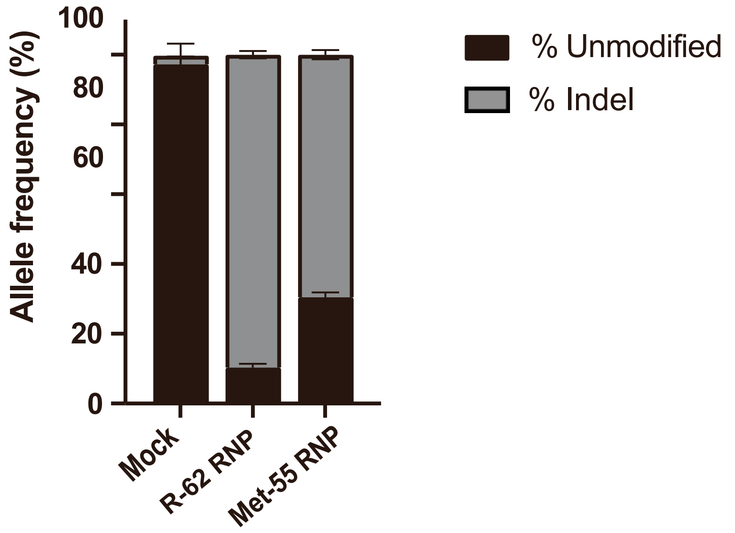

Figure S10. High bulk editing rates for R-62 and Met-55 gRNA in SHD$^{GFP/BFP}$. NGS editing quantification demonstrating high editing rates in SHD$^{GFP/BFP}$ for both gRNAs.

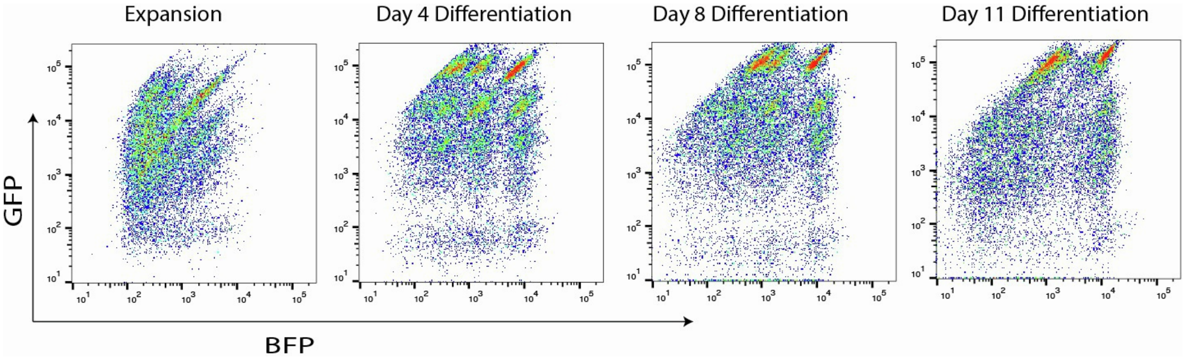

Figure S11. Beta-globin expression and cellular viability drive changes in GFP and BFP MFI. GFP and BFP MFI were measured throughout the in vitro culture of the R-66S RNP-treated SHDGFP/BFP model, demonstrating changes in MFI with differentiation. A clear distinction between the unique clusters is apparent only during erythroid differentiation after culture, with induction of β-globin transcription and translation. A clear pattern was observed as early as 2 days after the initiation of differentiation. However, late in differentiation (day 11), clusters based on GFP and BFP MFI are less distinct due to decreased cell viability and health.

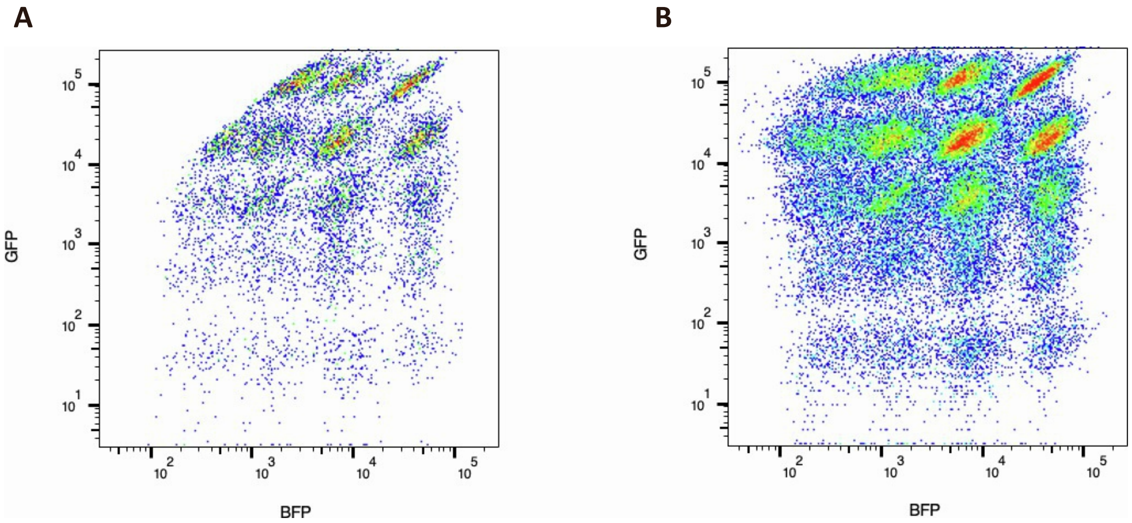

Figure S12. Improved separation of clusters after R-66S RNP editing based on GFP and BFP MFI. BFP MFI is lower than GFP MFI, resulting in poor separation, particularly when GFP MFI is high. We improved cluster separation after R-66S editing by generating a clone from the initial SHD$^{GFP/BFP}$ model and updating the experiment-specific compensation on the BD Melody flow cytometer. This resulted in improved separation after R-66S RNP editing. On day 4 of differentiation, we compared separation using standard BD Melody FC bead compensation with experiment-specific compensation using GFP- and BFP-single-fluorophore controls. **(A)** Separation seen after R-66S RNP gene editing on day 4 of erythroid differentiation with standard flow cytometry compensation. **(B)** Separation was observed using experiment-specific, updated compensation, resulting in improved distinction among clusters. Improved separation of GFP and BFP was observed in clusters with high GFP and varying BFP levels, allowing us to distinguish GFP^{high}BFP^{low} from GFP^{high}BFP^{neg}, resulting in higher resolution. The resolution was further improved by using the SONY MA900 cell sorter for subsequent experiments, as described in the main text.

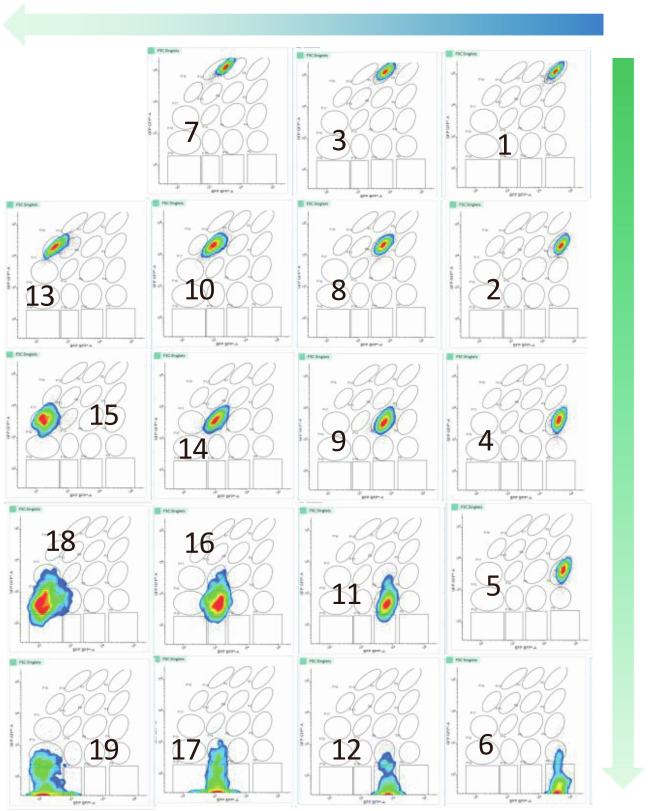

Figure S13. Unique clusters based on GFP and BFP MFI due to underlying genotypes. Bulk sorted 19 clusters defined based on GFP+BFP MFI remain within the initial gating used for sorting after further culture *in vitro,* confirming distinct MFI changes are due to unique genotypes in each cluster rather than *HBB* promoter strength and cell status. Clusters were numbered according to the sum of GFP and BFP MFI, ordered from highest (Cluster 1) to lowest (Cluster 19).

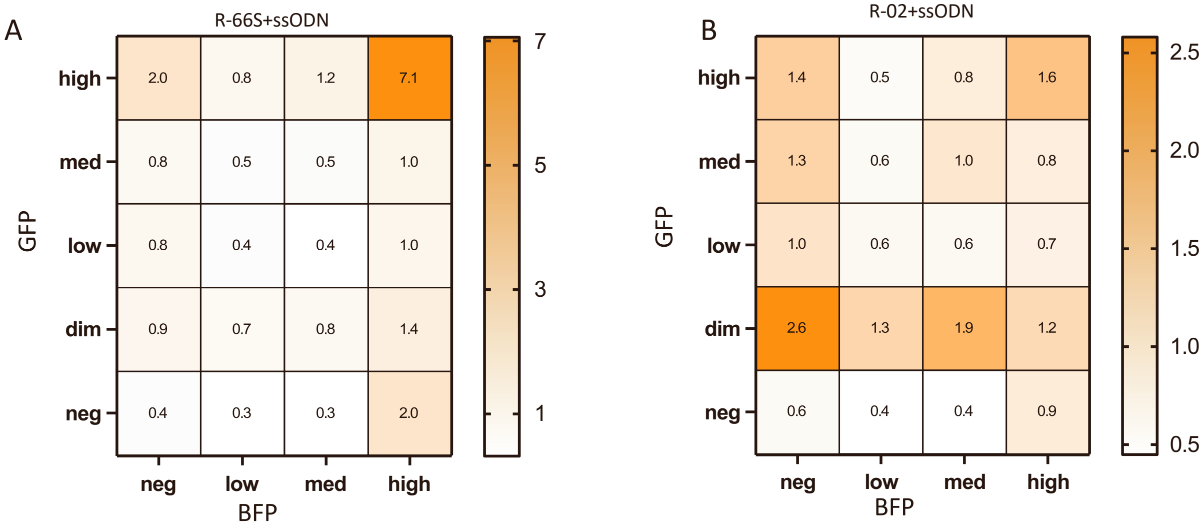

Figure S14. Addition of an ssODN corrective donor, delivered as an RNP, with both R-02 and R-66S results in a decrease in the percentage of cells with small indels and large deletions. Fold change in percentage of cells shown comparing **(A)** R-66S+ssODN to R-66S RNP and **(B)** R-02+ssODN and R-02 RNP alone. With both gRNAs, an overall decrease in clusters enriched in small indels and large deletions is seen. Heatmaps display means from n= 2 electroporations.

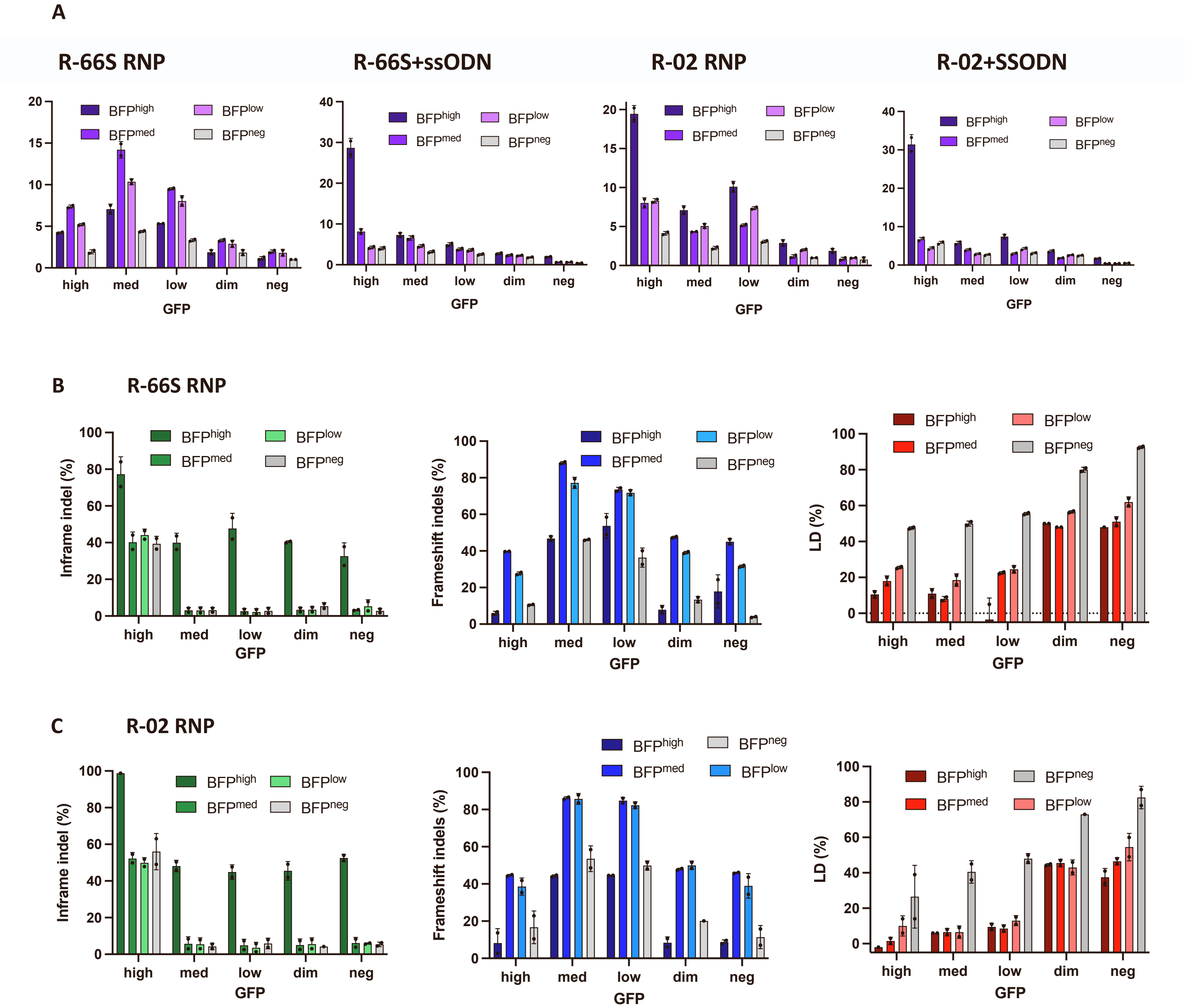

Figure S15. Cell number quantification and indel profiles across each cluster based on GFP and BFP MFI after R-66S and R-02 gRNA editing. (A) After efficient editing, the percentage of cells in each cluster reflects gRNA-specific editing outcomes across replicates, with per-replicate values and SD error bars. **(B)** Percentage of in-frame indels, frameshift indels, and LD across two independent replicates after R-66S RNP electroporation, replicate values and SD error bars shown. **(C)** Percentage of in-frame indels, frameshift indels, and LD across two independent replicates after R-02 RNP electroporation, individual replicates and SD error bars shown.

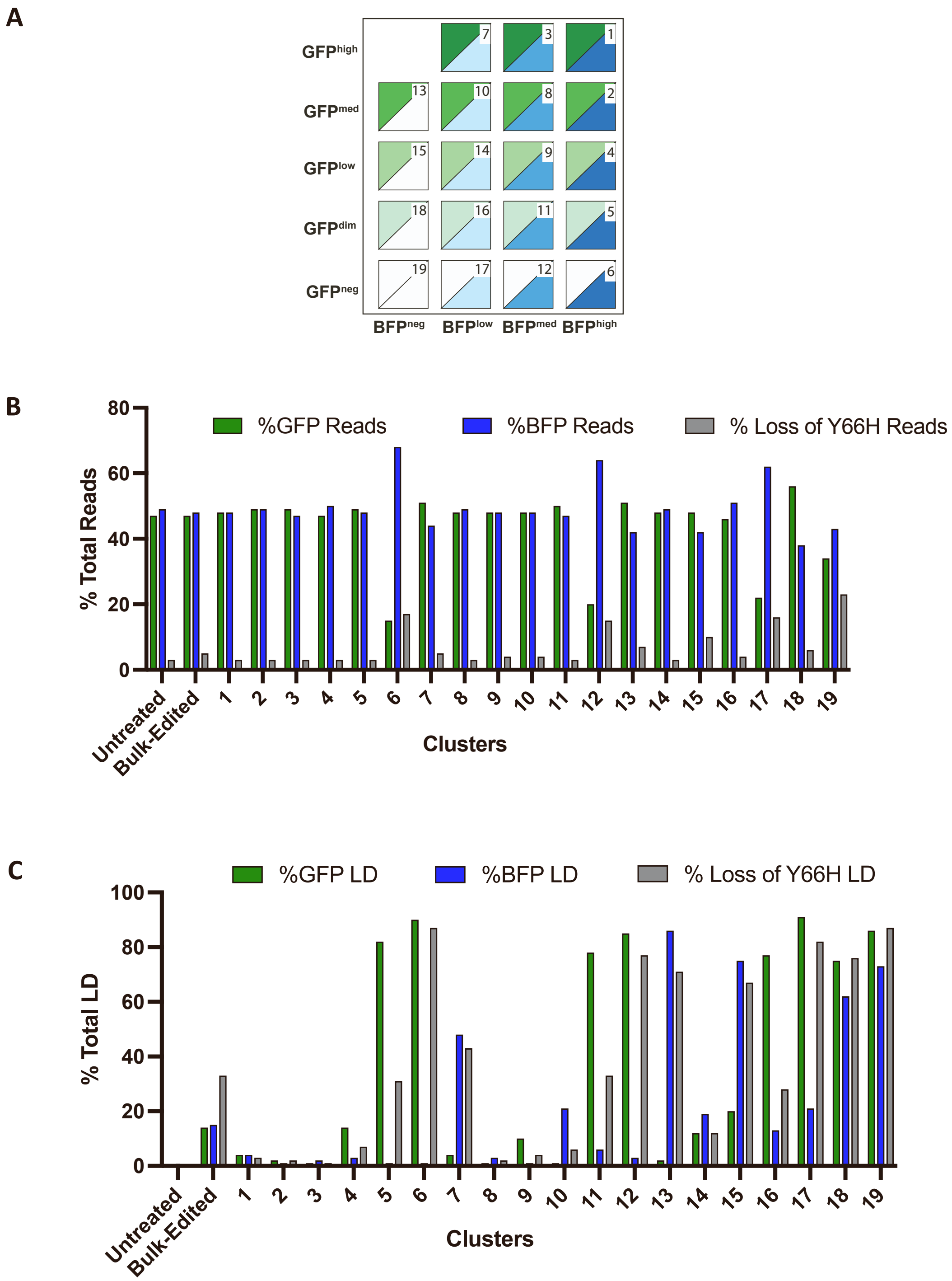

Figure S16. SMRT-Seq analysis enables accurate quantification of allele-specific editing. SMRT-seq analysis on R-66S RNP edited SHD$^{GFP/BFP}$ sorted into 19 clusters as shown in Figure S13 and applied to long-range PCR encompassing R-66S cut site, and Y66H mutation site, and reads were mapped to the corresponding GFP and BFP allele. Additionally, "loss of Y66H" alleles were identified, corresponding to reads with large deletions extending beyond the SNP, making it impossible to map the read to a specific GFP or BFP allele. **(A)** Previously defined cluster numbering 1-19 based on MFI **(B)** Allele-specific reads mapped based on GFP and BFP for unedited control, bulk edited and sorted clusters (1-19). **(C)** Large deletion rates across sequenced reads for each cluster demonstrate enrichment of large deletions with loss of GFP or BFP in addition to high rates of large deletions or large gene modification resulting in loss of the Y66H mutation site.

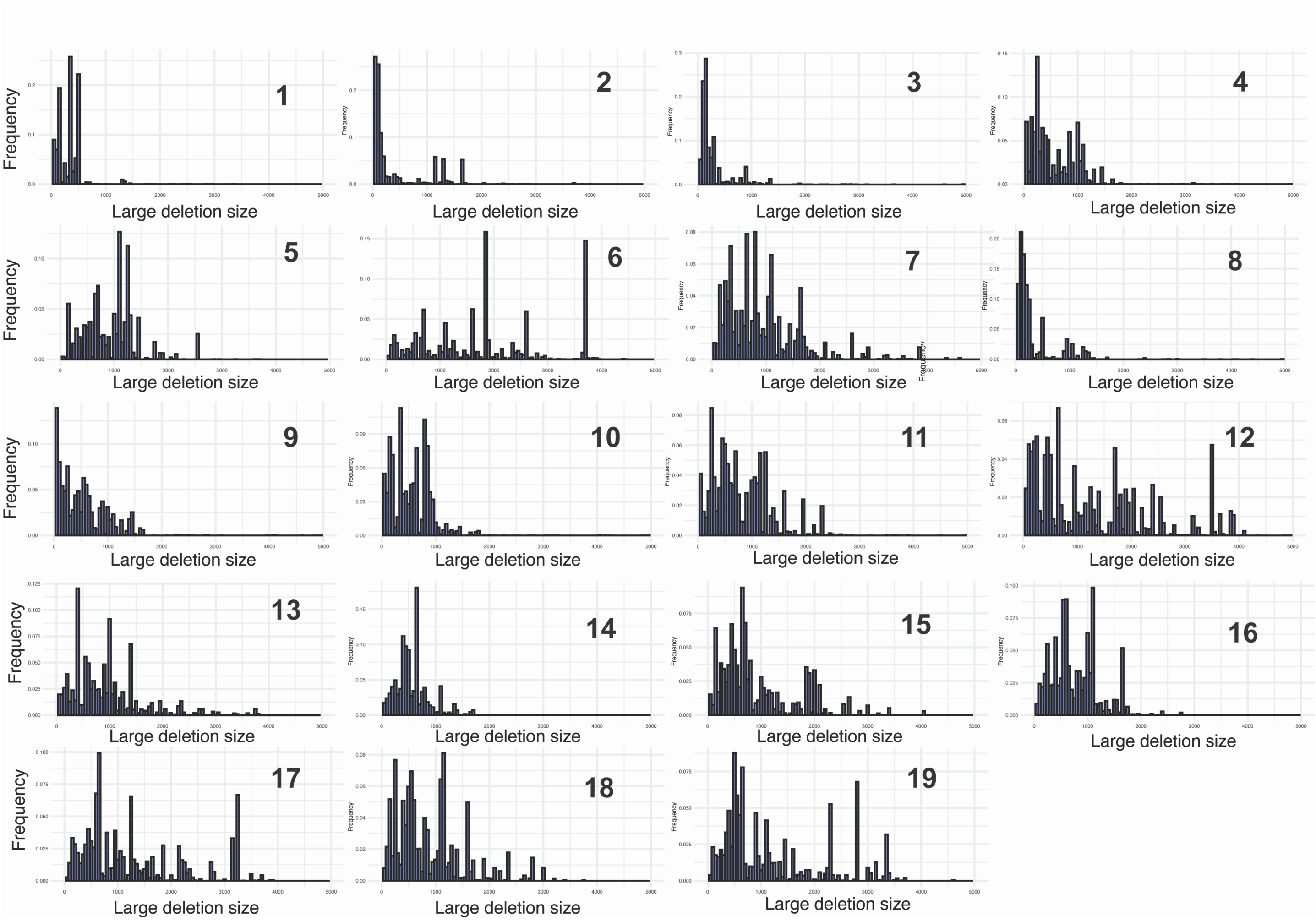

Figure S17. SMRT-Seq analysis enables the identification of large deletions that are missed in bulk cellular analysis after R-66S RNP editing. SMRT-seq enables size distribution analysis of large deletions in each of the predefined 19 clusters after R-66S RNP editing. High rates of very large deletions are seen in GFP^{neg} and/or BFP^{neg} clusters.

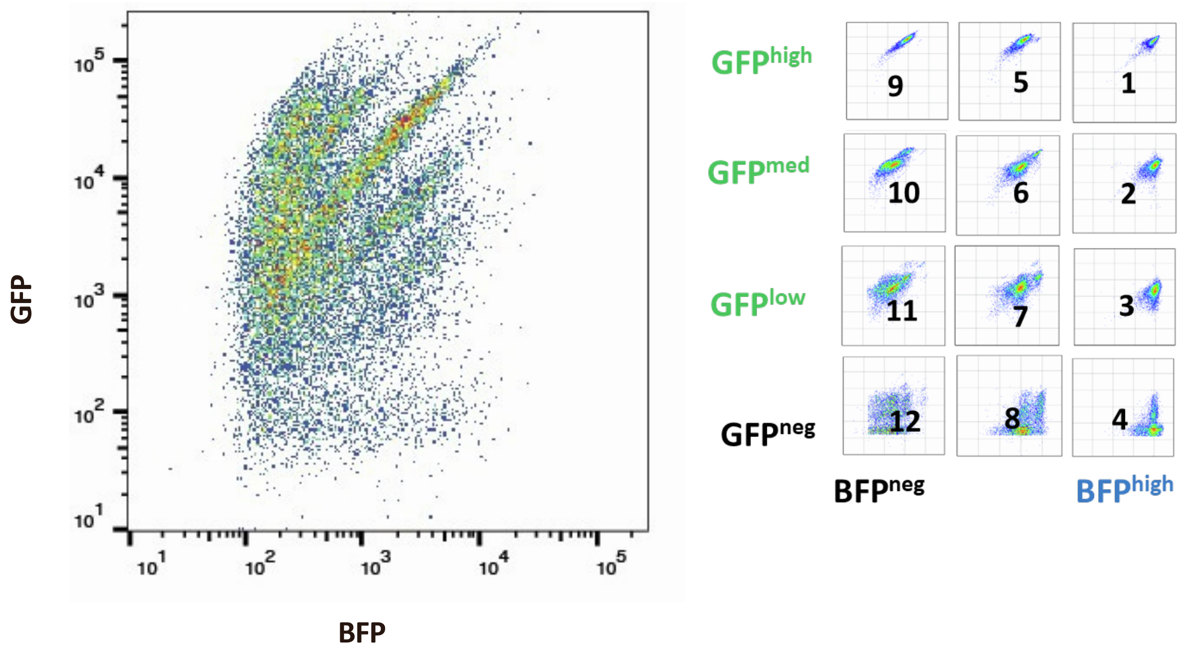

Figure S18. Unique clusters can be identified based on GFP and BFP MFI in bulk R-66S RNP *in vitro* expansion culture. GFP and BFP MFI changes were observed in expansion culture after R-66S RNP delivery, indicating that 12 unique clusters were used for mRNA and protein analyses due to a short lifespan after differentiation and poor expansion. Despite less distinct separation due to lower beta-globin promoter activity during expansion culture, 12 sorted clusters remained within the initial gate after *in vitro* expansion.

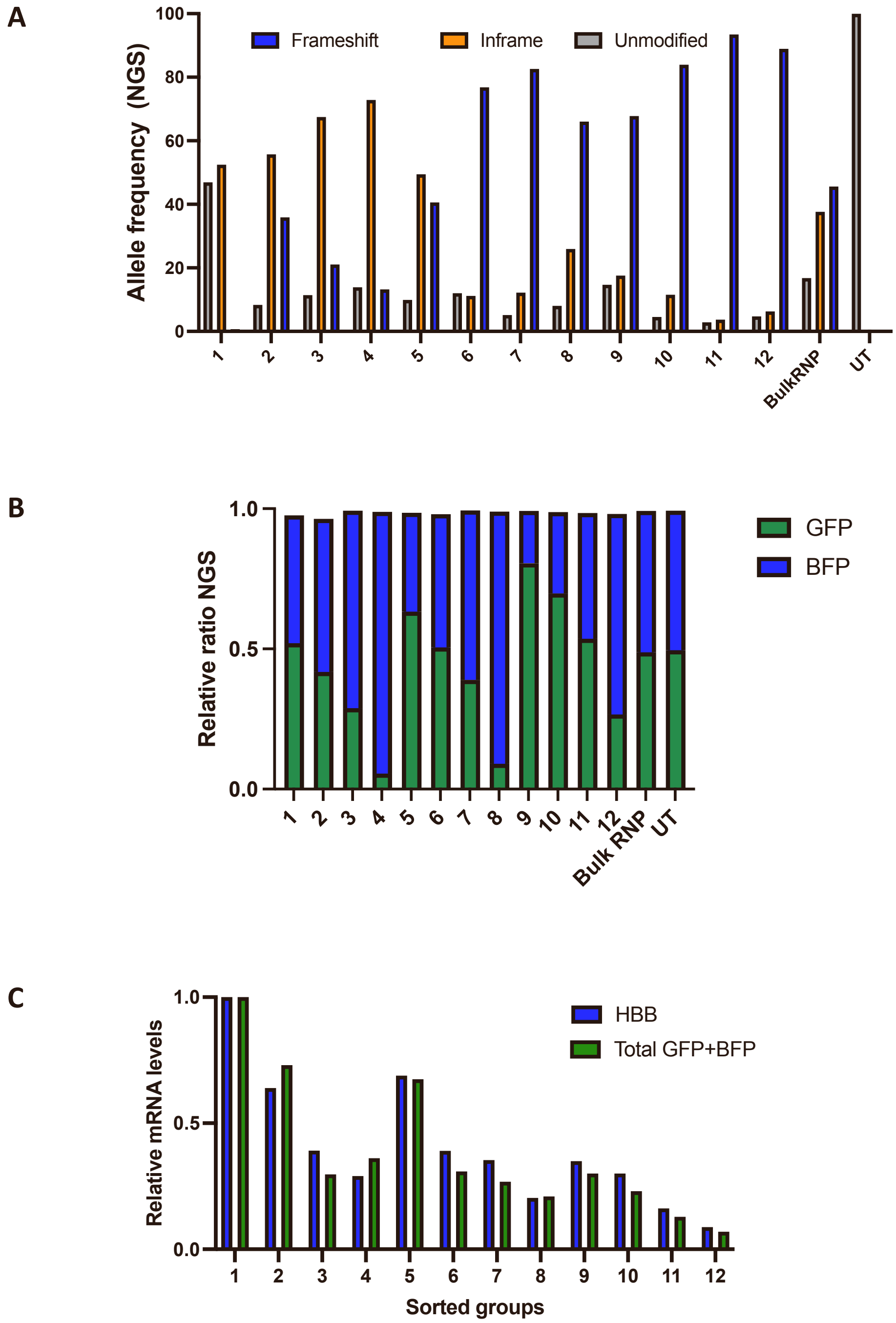

Figure S19. GFP and BFP expressions correlate clearly with HBB expressions across 12 sorted clusters in expansion culture after R-66S RNP delivery based on cDNA and mRNA levels. (A) cDNA NGS performed on clusters 1-12, demonstrating similar incidence in frameshift indels as seen in gDNA NGS, confirming that certain frameshift indels allow for transcription. **(B)** cDNA NGS showing relative ratio of GFP to BFP in clusters 1-12, bulk RNP treated and UT samples, a clear 1:1 ratio of GFP to BFP seen in untreated control, and changes in clusters 1-12 correlate with changes seen in MFI. **(C)** qPCR showing relative mRNA levels of HBB and GFP+BFP demonstrating a clear correlation of GFP/BFP expression with HBB expression.

A

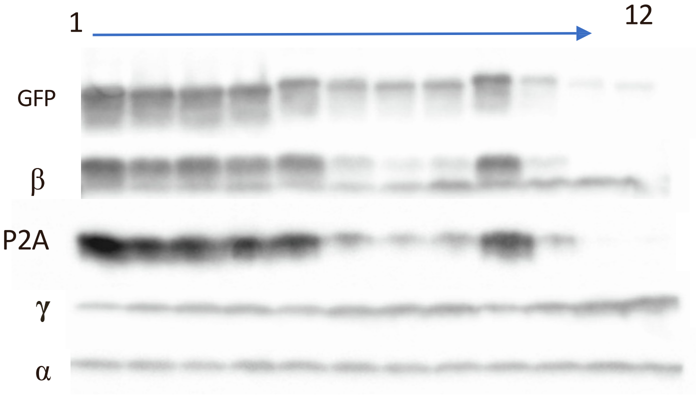

B

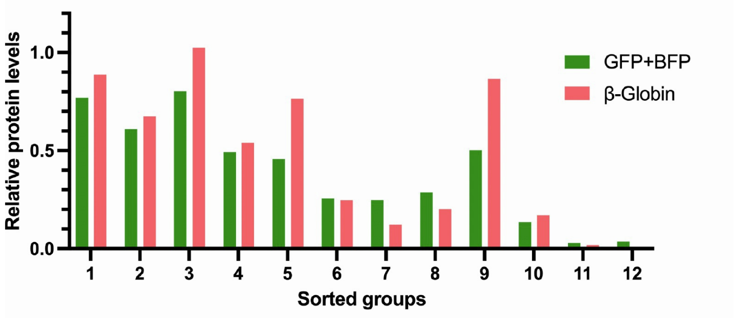

Figure S20. Protein analysis demonstrates a clear correlation between GFP/BFP translation and HBB translation (A) Western blot imaging of each 1-12 sorted cluster in expansion culture for GFP+BFP, β-globin, P2A, γ-globin, and α-globin. A clear correlation between loss of GFP+BFP protein production and β-globin production, with stable γ- and α-globin production, was observed. **(B)** Western blot relative quantification analysis using ImageJ of GFP+BFP and β-globin normalized to α-globin for each sorted cluster, demonstrating a strong correlation between GFP/BFP expression and β-globin production.

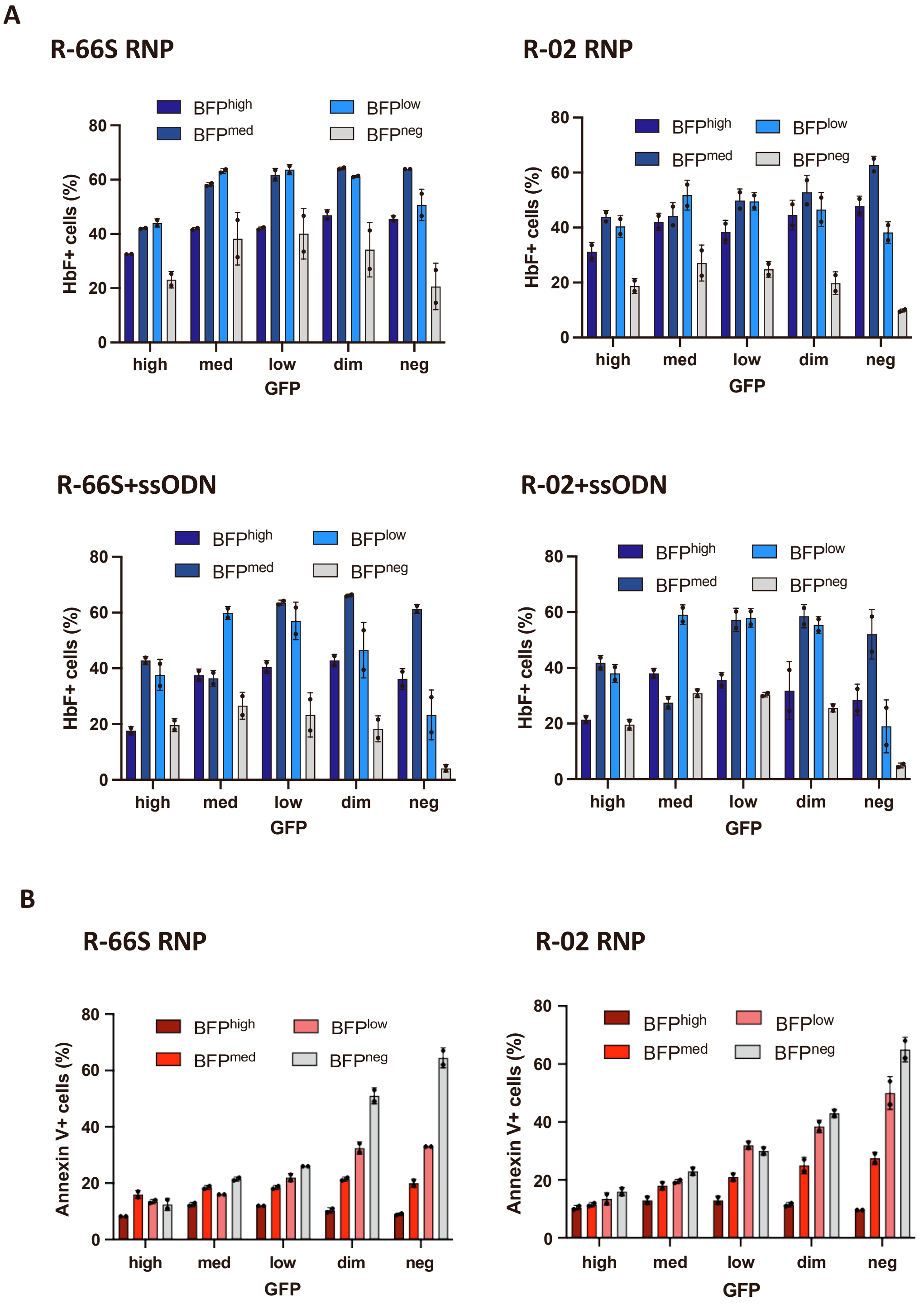

Figure S21. HbF induction and Annexin V+ vary significantly across clusters after R-66S and R-02 therapeutic gene editing, attributable to distinct genotypes. (A) After efficient editing, the percentage of HbF+ cells in each cluster reflects gRNA-specific editing outcomes with and without ssODN correction, shown across replicates with per-replicate values and SD error bars. **(B)** Percentage Annexin V+ cells in each cluster across two independent replicates after R-66S RNP and R-02 RNP electroporation, replicate values and SD error bars shown.

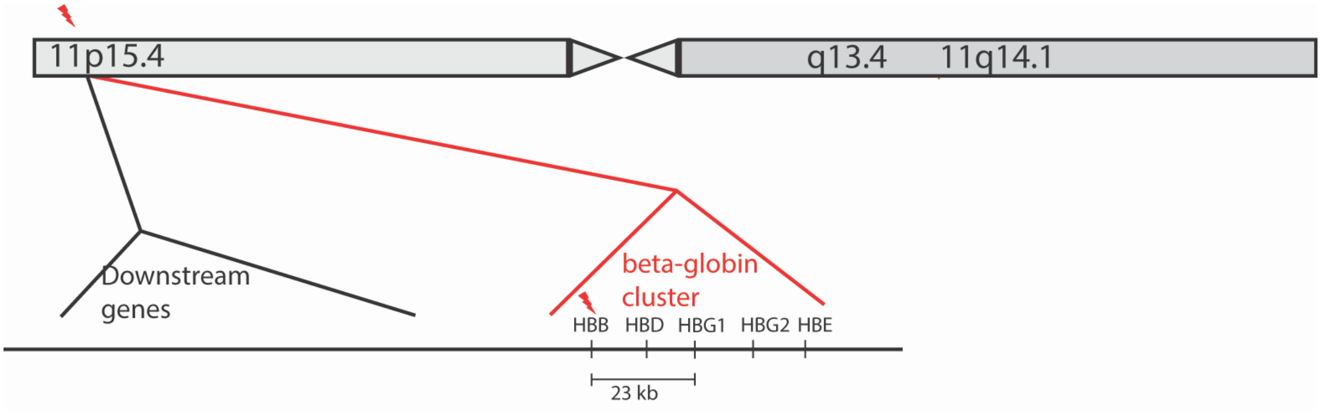

Figure S22. Schematic of the globin locus. HBG is located ~21 kb upstream of HBB, and large deletions extending beyond HBB could disrupt the HBG region.

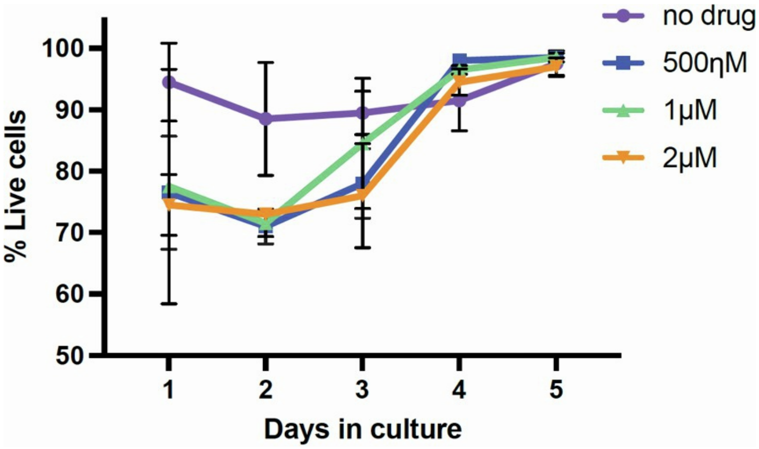

Figure S23. M3814 was well-tolerated by SHD$^{GFP/BFP}$. M3814 dose curve well tolerated by SHD$^{GFP/BFP}$ based on cell viability measured via trypan blue. Initial toxicity was seen with drug incubation across all doses; however, recovery was seen within 4 days of editing based on viability. N=2, technical replicates.

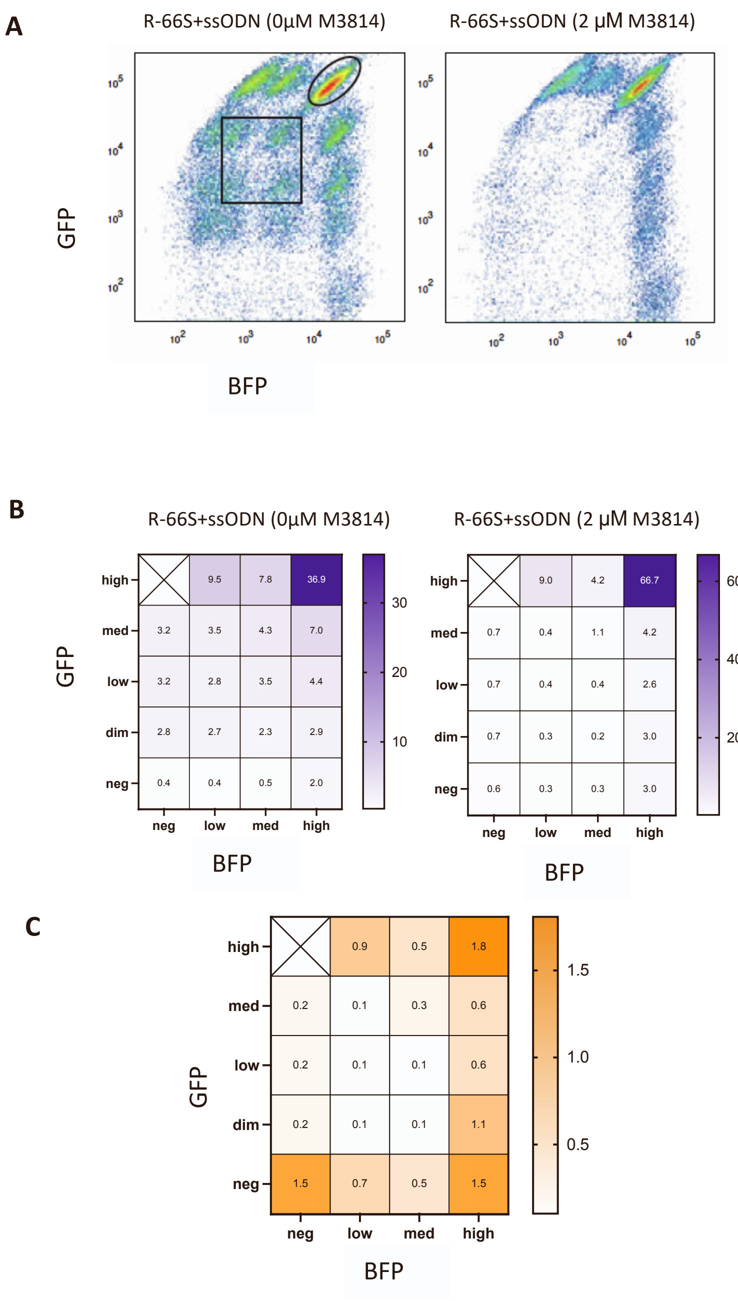

Figure S24. Validated platform for evaluating the impact of HDR enhancer M3814 on HDR rates and DSB cellular repair mechanisms. R-66S RNP and ssODN delivered via electroporation to SHD$^{GFP/BFP}$ followed by treatment with M3814, a potent DNA-dependent protein kinase (DNA-PK) inhibitor. Dose curve from 0.5 to 2 μM; M3814 was added immediately post-editing, and cells were incubated with M3814 for 24 hours, after which M3814 was removed. **(A)** Inhibition of NHEJ resulted in a dose-dependent increase in GFP^{high}BFP^{high} cells on day 4 of erythroid differentiation, indicating enhanced HDR. **(B)** The percentage of cells across 20 previously defined clusters was quantified after R-66S+ssODN editing, with and without M3814 incubation. Again, demonstrating an increase in the percentage of GFP^{high}BFP^{high} cells with the addition of M3814. Additionally, blocking NHEJ with M3814 reduces the percentage of cells enriched for bi-allelic frameshift indels, as evidenced by fewer cells in clusters with intermediate GFP and BFP. However, an increase in GFP^{neg}BFP^{neg} and GFP^{neg}BFP^{high} clusters raises concern about higher rates of large deletions and LOA with NHEJ inhibition, although this is not statistically significant. N=2, technical replicates**.** **(C)** Fold change in percentage of cells in each cluster after R-66S+ssODN editing with and without M3814 incubation.

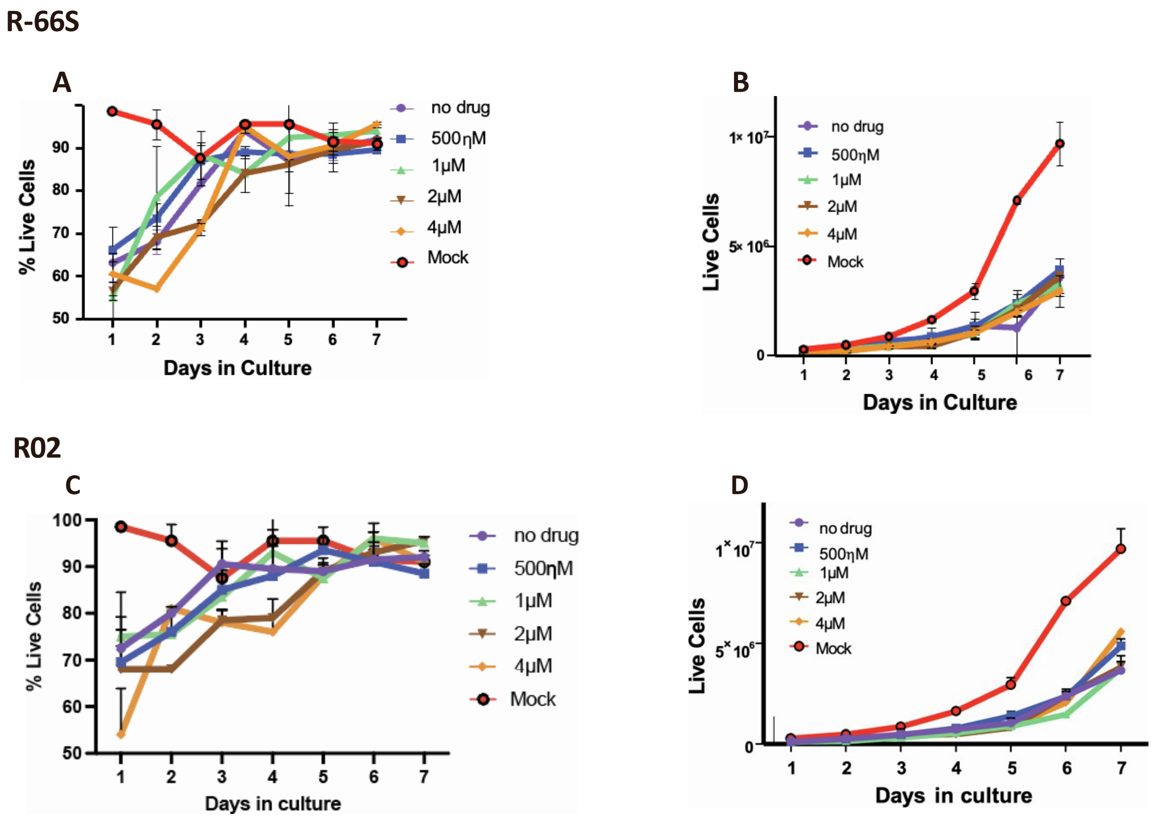

Figure S25. M3814 was well tolerated by patient-derived HSPCs after R-66S+ ssODN and R-02 ssODN gene editing *in vitro*. Cell viability was measured by trypan blue staining, and expansion was assessed daily for 7 days after electroporation and M3814 drug treatment. **(A)** Impact of M3814 on cell viability following R-66S+ssODN *in vitro* editing based on trypan blue staining. Initial toxicity was seen with electroporation and drug treatment, with the most significant toxicity seen in the highest drug concentration; however, recovery was seen across all conditions. **(B)** Preserved cellular expansion with M3814 treatment and R-66S +ssODN electroporation. Among edited samples, no significant difference in expansion was observed across groups, regardless of M3814 dose. Slow expansion, as expected, was seen within the initial 72-96 hours of electroporation, followed by a more rapid expansion rate. Mock-treated control showed expected robust expansion throughout. **(C)** Impact of M3814 on cell viability following R-02 + ssODN *in vitro* editing based on trypan blue staining. Dose-dependent toxicity seen with initial M3814 incubation followed by complete recovery by day 7 of *in vitro* culture. **(D)** Preserved cellular expansion with M3814 treatment after electroporation with R-02+ssODN. Slowed expansion with electroporation and drug treatment, like results seen with R-66S +ssODN. No significant difference was observed in the M3814 dose curve. N=2, technical replicates.

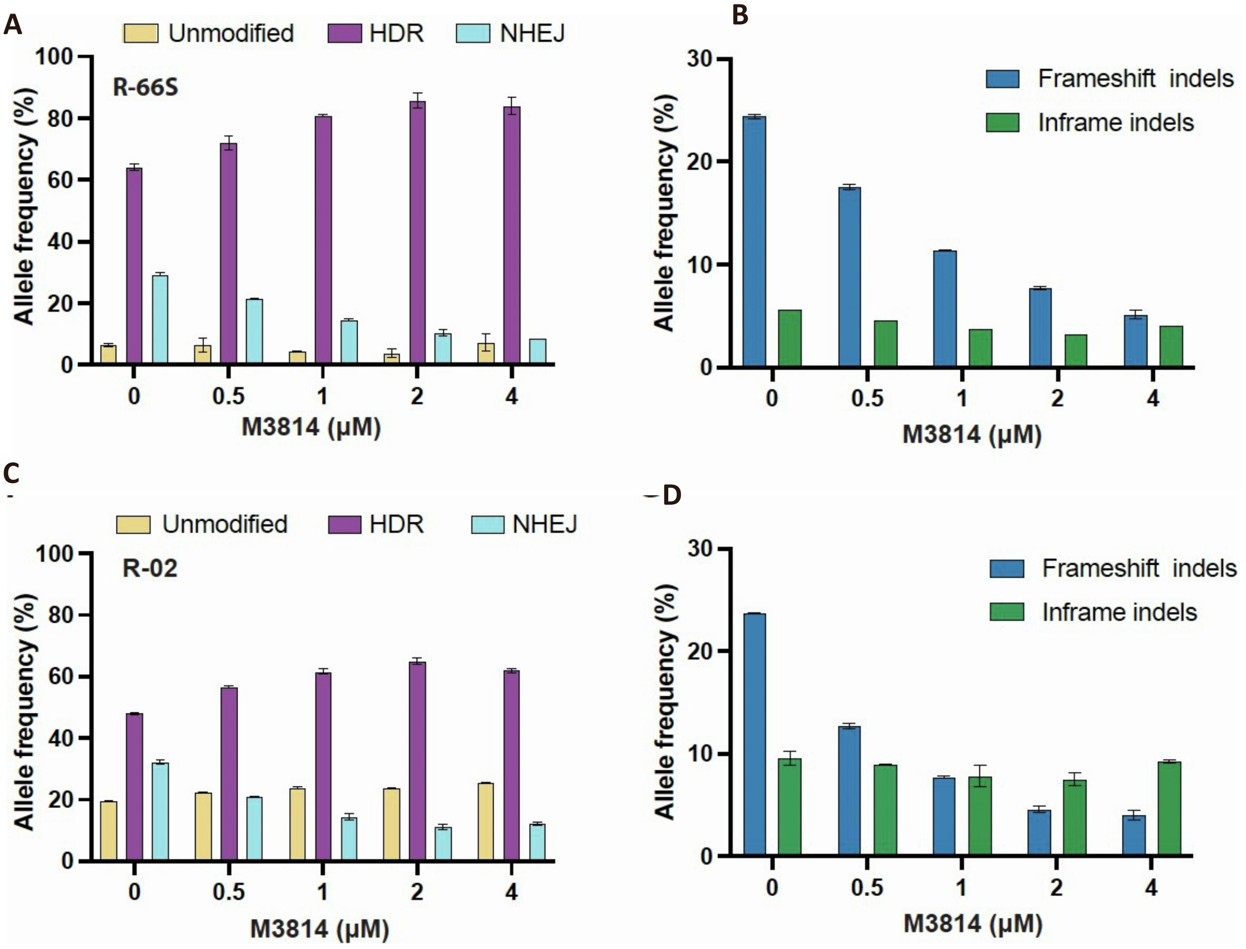

Figure S26. M3814 results in a dose-dependent increase in HDR in patient-derived HSPCs. (A) NGS analysis of SCD HSPCs edited with R-66S RNP + ssODN and treated with M3814 confirmed a dose-dependent increase in HDR and a decrease in NHEJ. After delivering R-66S + ssODN and incubating the cells with M3814 for 24 hours, HDR increased dose-dependently from 64.2 $\pm$ 1% without M3814 to 84 $\pm$ 2.9% with 4 µM M3814. However, no significant difference in HDR was observed between 2 and 4 µM, likely because initial toxicity at the higher dose affected cell cycle and repair. **(B)** A dose-dependent decrease in frameshift indels was observed in SCD HSPCs after R-66S+ssODN editing. The frequency of in-frame indels remained relatively steady, likely due to MMEJ-mediated repair of major in-frame indels, such as the 12bp deletion. **(C, D)** A similar dose-dependent increase in HDR and a reduction in frameshift indels were observed with R-02 + ssODN in SCD HSPCs. The R-02 gRNA generates MMEJ-mediated 9bp deletion as a major indel and a higher percentage of in-frame indels compared to R-66S.

A

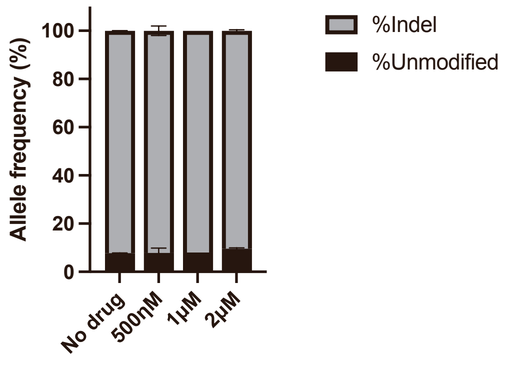

B

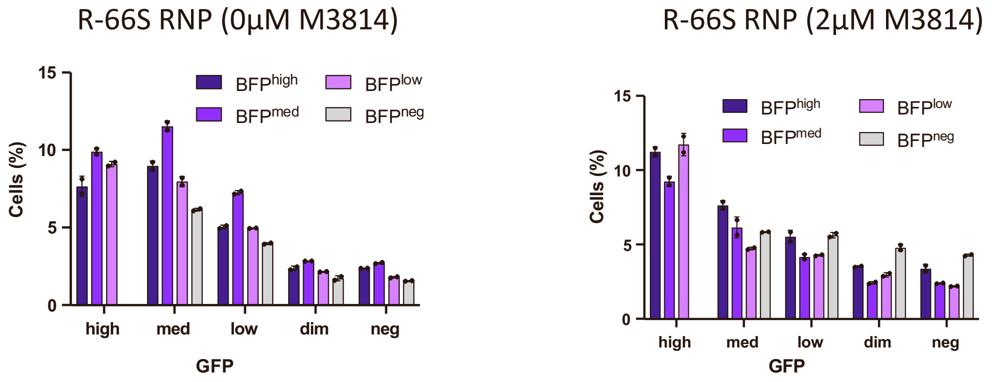

Figure S27. Percentage of cells in each cluster based on GFP and BFP MFI after R-66S RNP editing with and without M3814 incubation for each independent replicate. (A) Efficient editing seen SHD$^{GFP/BFP.}$ Cells were electroporated with R-66S RNP and immediately placed in media containing varying concentrations of M3814. After 24 hours of exposure, the drug was removed, and the cells were cultured for an additional 72 hours until editing was complete. **(B)** Cell distribution for two replicates is shown (independent electroporation) with SD error bars for cells treated with or without 2μM of M3814 after R-66S RNP editing.

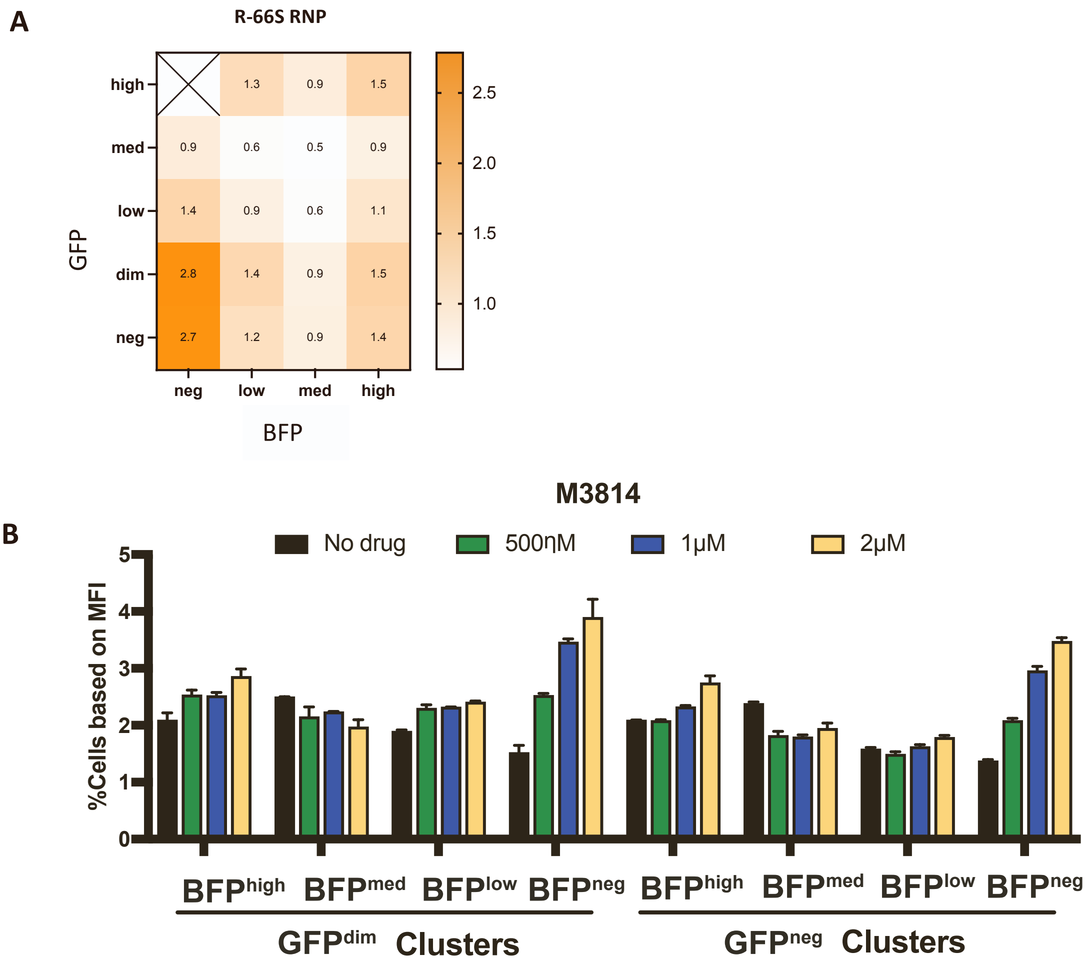

Figure S28 M3814 HDR enhancer results in a dose-dependent increase in large deletions in RNP-treated SHD$^{GFP/BFP}$. (A) The percentage of cells in each previously defined cluster based on GFP and BFP MFI was determined, and the fold difference between RNP-only edited cells incubated with or without 2μM of M3814 was calculated. M3814 resulted in a dose-dependent decrease in intermediate clusters, enriched in frameshift alleles mediated by NHEJ, similar to results seen in R-66S+ssODN conditions. However, a significant increase in GFP^{dim}/BFP^{neg} (p=0.0062) and GFP^{neg}BFP^{neg} cells (p=0.0049) was seen. **(B)** M3814 treatment also resulted in a dose-dependent relative increase in clusters enriched for large deletions, mediated via MMEJ and larger gene modifications or LOA, as evidenced by a significant dose-dependent increase in the percentage of GFP^{dim}/BFP^{neg} and GFP^{neg}/BFP^{neg} cells.

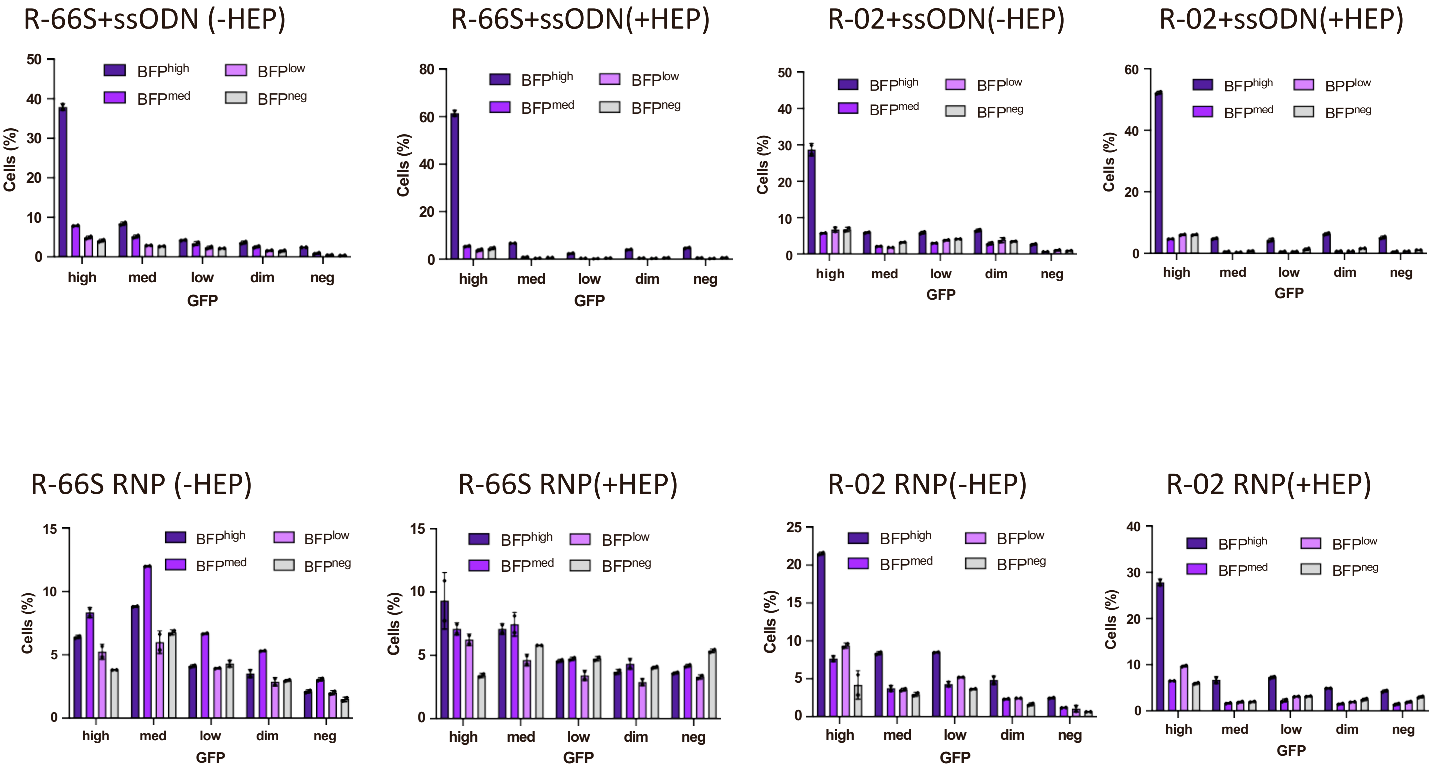

Figure S29. Percentages of cells in each individual cluster after therapeutic R-02 and R-66S editing in SHD$^{GFP/BFP}$ with or without the addition of 25 µM of HEP. Percentage of cells in each previously defined 20 clusters determined after 4 days of erythroid differentiation for each condition: n=2 individual replicates shown with SD error bars.

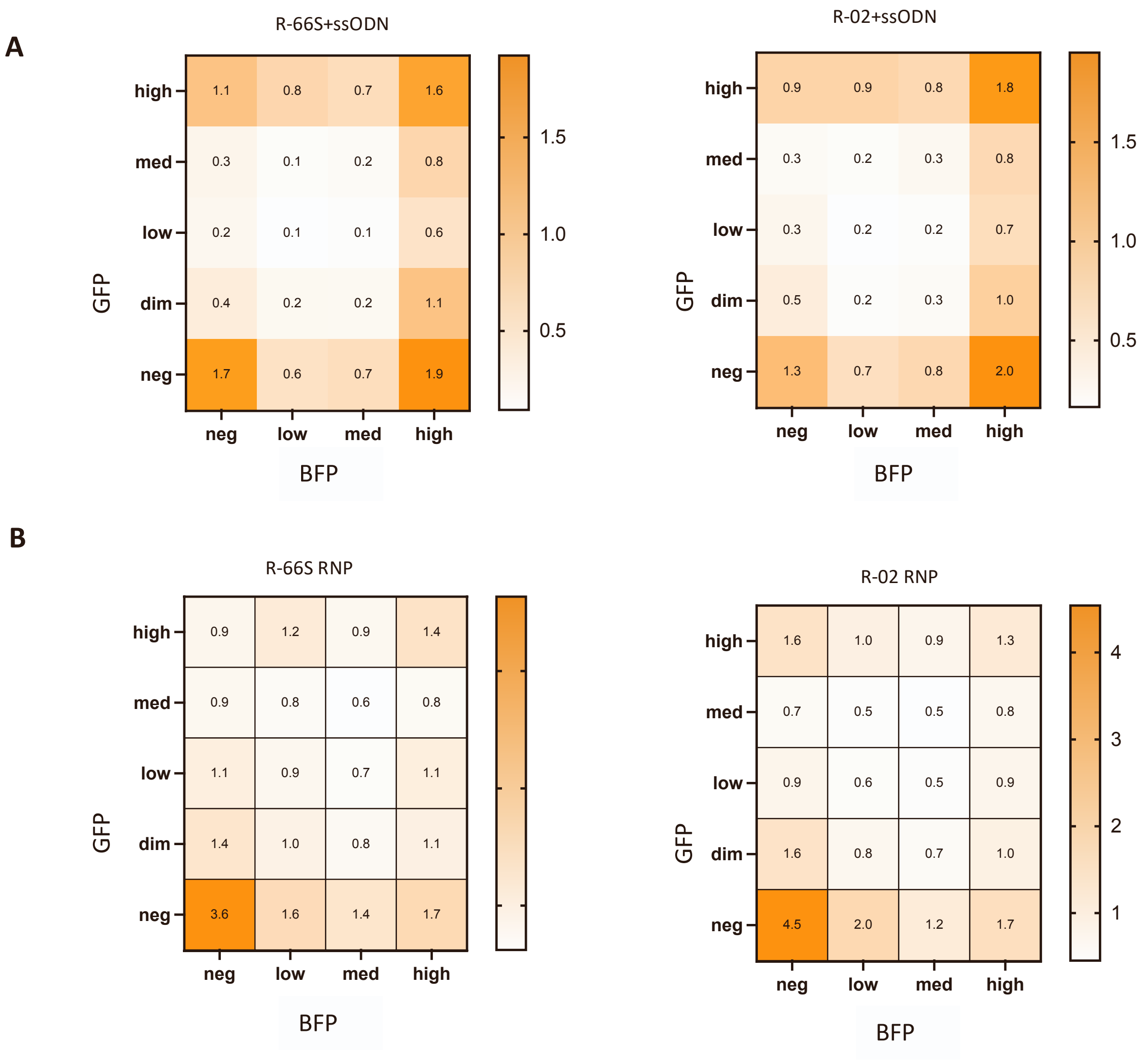

Figure S30. Promoting end resection with HEP results in an increase in HDR in the presence of a corrective donor, but also a significant increase in LOA rates in RNP only treated SHD$^{GFP/BFP}$. (A) The percentage of cells in each previously defined cluster based on GFP and BFP MFI was determined, and the fold difference between RNP+ssODN with or without the presence of 25 µM of HEP was calculated. An increase in the percentage of GFP^{high}BFP^{high} cells was seen with HEP treatment, consistent with an increase in HDR rates. However, although there was no significant increase in the percentages of GFP^{neg}BFP^{neg} and GFP^{neg}BFP^{high} in RNP+ssODN-edited cells, this raises concern that an increase in LD and LOA events is observed alongside HDR enhancement with HEP treatment. **(B)** The percentage of cells in each cluster with and without HEP treatment after RNP-only editing was then examined. Without the addition of the ssODN corrective donor, a significant fold increase in GFP^{neg}BFP^{neg} cells was observed for both R-66S and R-02 RNP (p=0.002, p=0.04, respectively).

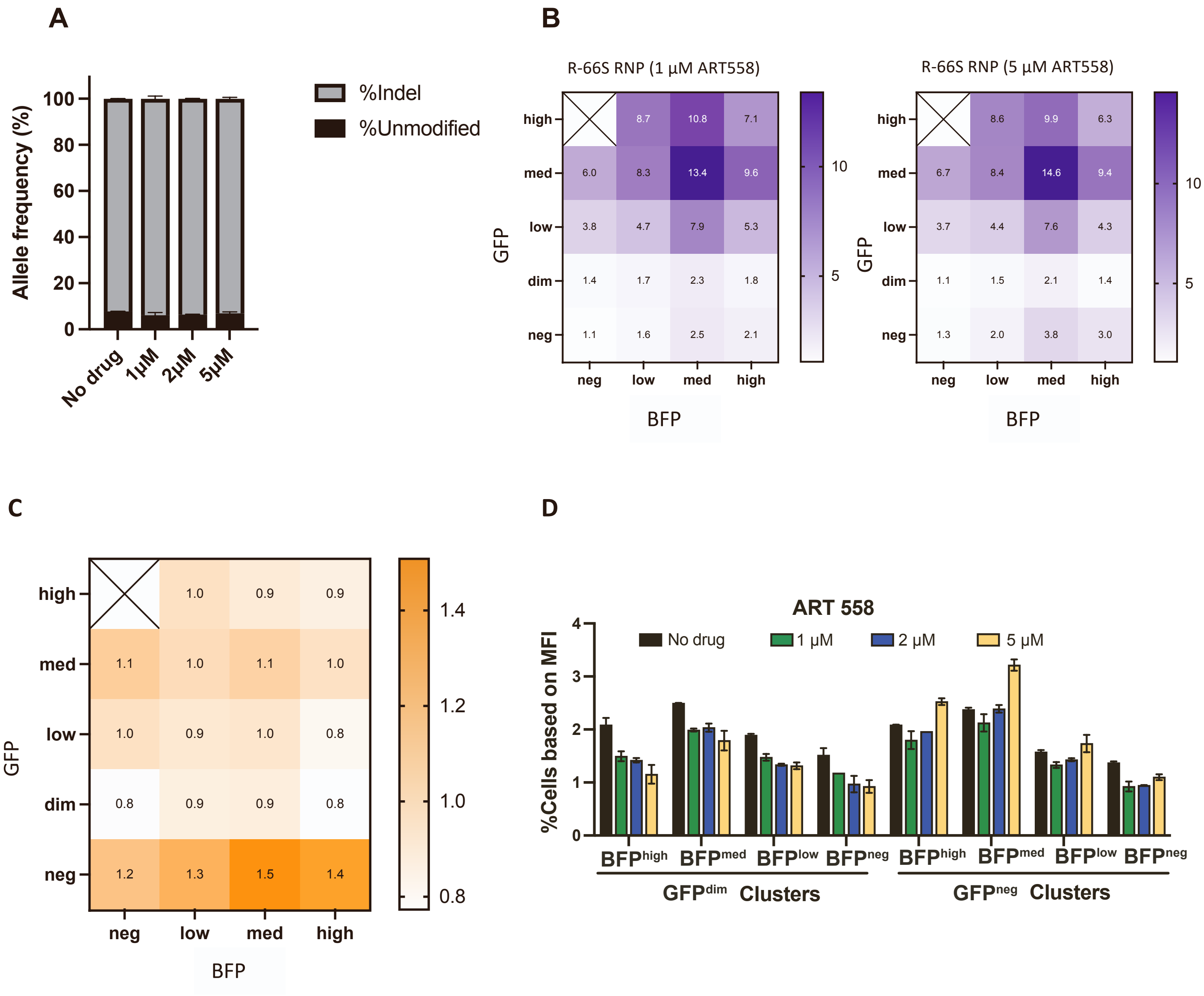

Figure S31. ART558, a polymerase theta inhibitor, inhibits MMEJ and reduces large deletions in RNP-treated SHDGFP/BFP, but increases the rate of LOA. **(A)** Efficient editing seen SHD$^{GFP/BFP.}$ Cells were electroporated with R-66S RNP and immediately placed in media containing varying concentrations of ART558. After 24 hours of exposure, the drug was removed, and the cells were cultured for an additional 72 hours until editing was complete. **(B)** The percentage of cells in the previously defined 20 clusters was analyzed by flow cytometry, using changes in GFP and BFP MFI. Treatment with ART558 results in a dose-dependent reduction in GFP^{dim} clusters, where large deletions are repaired via MMEJ. Conversely, a dose-dependent increase in GFP^{neg} clusters indicates that MMEJ does not mediate LOA or large gene modification events. **(C)** Relative fold change in % of cells in cells treated with 1 µM of vs 5µM of ART is shown, and a clear increase is seen in the percentage of GFP^{neg} cells. **(D)** MMEJ inhibition (ART 558) increases the percentage of cells carrying larger gene modifications. Treatment with ART558 results in a dose-dependent reduction in GFP^{dim} clusters, where large deletions are repaired via MMEJ. Conversely, a dose-dependent increase in GFP^{neg} clusters indicates MMEJ does not mediate LOA events.
